# What Random Splits Measure in Polymer Glass-Transition Prediction: Chemistry Holdouts, Applicability Domains, and Prior Integration

**DOI:** 10.3390/polym18192445

**Published:** 2026-10-07

**Authors:** Deao Han, Yangkun Shao, Xueru Zhong, Zhanzhao Song, Quanzhong Zhang, Chuansheng Wang, Deshang Han, Lin Wang

**Affiliations:** 1 College of Transportation, Ludong University, Yantai 264025, China; 2 Analysis and Testing Center, Ludong University, Yantai 264025, China; 3 Graduate School of Convergence Science and Technology, Seoul National University, Seoul 08826, Republic of Korea; 4 National Engineering Laboratory for Advanced Tire Equipment and Key Materials, Qingdao University of Science and Technology, Qingdao 266061, China

**Keywords:** glass-transition temperature, polymer informatics, model validation, applicability domain, group contribution

## Abstract

Predicting the glass-transition temperature (Tg) helps select polymers for a service-temperature window, but accuracy on familiar structures may not transfer to a new family. We compare validation protocols on 7367 curated homopolymers. Histogram gradient boosting gives R^2^ = 0.899 and mean absolute error (MAE) = 23.9 K under random cross-validation, 0.857 and 28.5 K under scaffold holdout, a class-macro mean of 0.355 and 43.3 K under curator-class holdout, and −3.79 and 79.5 K under high-Tg extrapolation. Similarity explains only 6–11% of within-protocol absolute-error variance in the first three settings. A separate density functional theory (DFT) band-gap dataset shows a moderate fingerprint-cluster holdout penalty and poor property-range extrapolation for the tested baselines. Random-forest split-conformal empirical coverage falls from 0.899 under random splitting to 0.683 and 0.214 under class and property holdout; the tested prospective similarity gate provides little protection. A fold-fitted group-contribution prior is more sensitive to uniform additive bias in a squared-loss penalty than as a neural-network input, but this comparison is architecture-dependent. Class-holdout intervals and tests are descriptive or nominal because training sets overlap. The 279 multiply reported polymers support only the between-source variability analysis. Validation should match the intended polymer family and property range.

## 1. Introduction

The glass-transition temperature (Tg) helps determine whether the amorphous part of a polymer is glassy or rubbery at its intended use temperature. For a polymer scientist screening new repeat units, an error of several tens of kelvin can change which candidate is selected. Chain flexibility, bulky or rigid groups and intermolecular interactions make chemical structure informative, while molar mass, tacticity and thermal history also influence reported Tg. Classical group-contribution methods connect structural groups to an empirical estimate of Tg [1,2], alongside physical descriptions of segmental mobility [3] and established molecular-weight and time–temperature relations [4,5]. Machine learning offers another way to use accumulated structure–property measurements [6,7,8].

Existing work spans broad homopolymer prediction [9,10,11,12], systematic comparisons of representations and algorithms [13], and models tailored to particular polymer families. Jiang et al. studied Tg in polyhydroxyalkanoate homo- and copolymers using structural fingerprints together with composition, molecular weight and dispersity [14]. Sotiropoulos et al. modelled thermal properties of PHB/PHBV-based materials using an integrated database and material-level variables [15]. These studies address related but different prediction tasks: success within a specified family or with additional measured inputs is not automatically evidence for repeat-unit-only prediction of an unmeasured family. PoLyInfo [16] and informatics resources such as the PI1M structure library [17] have expanded the data infrastructure, but a useful accuracy statement still needs to specify which polymers are being predicted.

Consider two design decisions: choosing a new side group within a well-studied polyester series, and predicting a family with a backbone absent from the training collection. A random train–test split usually leaves close analogues of the first candidate in training. Holding out a whole named family makes the second decision more explicit. Random splitting is a valid test of interpolation in the sampled collection; it does not by itself validate transfer to a new family. This distinction is established in cheminformatics and materials informatics [18,19,20,21,22,23,24]. The present question is its practical size for polymer Tg screening, including systematic temperature offsets, candidate ranking and the reliability of uncertainty intervals.

The PolyMetriX ecosystem [25] provides an openly licenced curated Tg dataset [26] and polymer-aware representations and splitters. We use this infrastructure to compare random, scaffold, curator-class and property-range holdouts while keeping each model and feature stream fixed. Our contribution is a controlled, polymer-specific evaluation of these choices, rather than the invention of non-random validation. The comparisons ask which structural families account for the degradation and whether the resulting errors would change a screening decision.

A second question concerns how to use an approximate group-contribution estimate alongside machine learning. Here the group increments are fitted only to each training fold; we do not test published handbook increments. We compare an additional input feature with a quadratic penalty pulling predictions towards that empirical prior. Although prior penalties occur in physics-informed learning [27,28], this penalty is not a differential-equation residual or a governing physical law. Its mathematical effect is target shrinkage, and disagreement between its gradients and the data gradients [29,30] must be interpreted in that limited setting. We test sensitivity to a uniform additive shift, for which the network architecture itself may favour the input-feature formulation.

The study uses transparent baselines to make the evaluation choices easy to inspect. The analyses address the following questions relevant to polymer selection:

1. How does accuracy change for familiar repeat units, withheld structural groups, withheld curator-defined families and temperatures above the training range? Two feature streams share the same partitions, and structural-identity checks accompany the comparison.
2. Which families show systematic temperature offsets, and how much information does structural similarity provide about error? Similarity is evaluated as a population-level diagnostic, not a guarantee for an individual candidate [31].
3. Do the models identify high-Tg candidates? Balanced accuracy and top-decile enrichment connect numerical errors to screening decisions, with uncertainty summaries tied to the sampling design.
4. How large are errors relative to disagreement between literature reports? The 279 polymers with multiple reports provide a variability reference, whose selection bias is stated explicitly.
5. Do uncertainty intervals and a prospectively chosen similarity threshold provide protection under a changed candidate population? These are stress tests of operational methods and include negative results.
6. How sensitive is an empirical prior to its integration rule and calibration, and which protocol effects also appear for another property? A controlled neural-network comparison examines prior integration; a separate dataset of 4127 density functional theory (DFT) polymer band gaps [32] tests matched holdout constructions.

The results are bounded to the dataset, descriptor families and transparent baselines studied here, and are stated as such in Section 4.5.

## 2. Materials and Methods

### 2.1. Open Dataset

All data are taken from the curated glass-transition temperature set released with the PolyMetriX ecosystem [25] and archived on Zenodo under CC-BY-4.0 [26]. The file LAMALAB_CURATED_Tg_structured_polymerclass.csv (SHA-256 b3b7e08a…a1079, recorded in full in the reproduction log) contains 7367 homopolymer records. Each record carries a polymer simplified molecular-input line-entry system (PSMILES) string for the repeat unit with two explicit polymerisation points, an experimental $T_{g}$ in kelvin (mean 417.1 K, standard deviation 112.7 K, range 134–768 K), and provenance metadata.

The curators assembled the table from nine literature sources and, where a polymer appears in more than one source, report the median of the reported values together with the individual source values, the number of contributing points and their standard deviation. One source (the GREA compilation) dominates the record count; the multi-source subset is small but is exactly what makes the reproducibility analysis of Section 2.7 possible. Each record also carries a curator-assigned polymer class drawn from a 22-term vocabulary (polyimides, polyoxides, polyesters, …), and 100 precomputed polymer-aware descriptors at the side-chain, backbone and full-repeat-unit levels. No new experimental measurement was performed for this work.

### 2.2. Curation and Leakage Audit Procedures

Records were required to parse under RDKit [33], to carry exactly two polymerisation points, and to have a finite $T_{g}$ inside 100–900 K. Structures were then reduced to three progressively looser identity keys:

1. The canonical isomeric simplified molecular-input line-entry system (SMILES) of the starred repeat unit;
2. The same string with stereochemistry removed;
3. The standard InChIKey of the *methyl-capped* repeat unit.

The third key requires comment. InChI cannot process wildcard atoms, so the two polymerisation points must be replaced before a standard identifier can be computed. Deleting them is not acceptable, because it merges constitutionally distinct repeat units—for example, a head-to-tail and a head-to-head linkage of the same composition collapse to the same fragment. Capping with methyl groups preserves the attachment topology and was used throughout. Records were deduplicated on the first key, taking the median $T_{g}$ of any duplicate group; stereoisomer pairs identified by the second key were assigned to a common scaffold grouping key for P2. The random, class and property partitions follow their respective constructions and do not enforce methyl-capped InChIKey grouping. The counts produced by each key are reported in Section 3.1. A SHA-256 digest of every canonical structure is stored so that the absence of overlap between training and test partitions can be verified by content hash rather than by row index; the realised canonical-structure hash overlap is zero in all 38 folds of all four protocols (audit_preprocessing_leakage.csv). This does not establish zero overlap under the looser identity keys. The 38 counts cover every partition that was materialised: 15 for P1 (a five-fold split repeated with three seeds), 5 for P2, 17 for P3 and 1 for P4. The fold-level *statistical* analyses of Section 3.4 use 28 of these, for the reason given there.

### 2.3. Two Feature Streams and Fold-Internal Preprocessing

Two independent representations share every split.

**Stream A** is the published polymer-aware descriptor set distributed with the dataset: side-chain counts and lengths, star-to-side-chain distances, backbone-level topological and ring descriptors, and full-repeat-unit physicochemical descriptors.

**Stream B** was computed here from the canonical structures alone and shares no code path with stream A: a count-based Morgan fingerprint of radius 2 folded to 1024 bits [34], concatenated with ten interpretable whole-repeat-unit descriptors (molar mass, logP, topological polar surface area, rotatable bonds, total and aromatic ring counts, hydrogen-bond donors and acceptors, fraction of sp3-hybridised carbon, heteroatom count).

Agreement between the streams would show that the observed ordering is not confined to either of these two representations; it would not establish representation independence in general.

**Preprocessing is fitted inside the fold.** Missing-value imputation, constant-feature removal and scaling were fitted exclusively on the training partition of each outer fold and then applied unchanged to the held-out partition, implemented as a scikit-learn Pipeline (median SimpleImputer → VarianceThreshold → StandardScaler → estimator; tree models use the same pipeline without the scaler). The same pipeline is nested inside the inner loop used for hyperparameter selection, so no preprocessing statistic is ever estimated on data that later serve as a test fold. No full-table imputation, variance filtering, scaling, feature selection or target-correlation filtering is performed at any point.

A retrospective audit confirms that this fold-internal construction changes nothing numerically on this dataset, which is itself worth reporting: stream A contains no missing and no infinite cells, so median imputation never alters a single value, and the in-fold variance filter removes exactly the same one constant column in every fold that a full-table filter would. Against a full-table variant, the largest absolute change in $R^{2}$ is 0.0000 and the largest in MAE is 0.0000 K across all four protocols, both feature streams and the fused tuned booster. The correct procedure is the one described above; the numbers in this paper are unchanged by adopting it.

### 2.4. Evaluation Protocols

Each split represents a different use case. P1 predicts another member of the sampled collection; P2 predicts a withheld structural grouping; P3 predicts a curator-defined family absent from training; P4 tests candidates above the training Tg range.

**P1, random**k**-fold (sanity check).** Five-fold cross-validation with shuffling, repeated three times with different seeds. This is the protocol used in most of the $T_{g}$ literature and is retained here as an optimistic interpolation reference.

**P2, scaffold-group holdout.** Repeat units were assigned a grouping key and partitioned with grouped five-fold cross-validation, so that no group is split across the boundary. The key is the Bemis–Murcko ring scaffold [20] of the star-deleted repeat unit when the structure contains a ring system. For acyclic repeat units, for which the Murcko scaffold is empty, a polymer-specific fallback is used: the element- and aromaticity-annotated skeleton of the shortest path connecting the two polymerisation points, that is, the topological backbone of the repeat unit. This fallback is the polymer analogue of a scaffold and prevents all acyclic structures from collapsing into a single degenerate group.

**P3, leave-one-polymer-class-out**. Each curator-assigned polymer class with at least 40 members is held out entirely, and the model is trained on all remaining classes, giving 17 folds. This protocol operationalises the deployment question posed in the Introduction—prediction for a polymer family withheld as a curator-defined class—rather than reproducing it exactly. The heterogeneous Other class bin was excluded because it is not a chemistry. Some class boundaries in the source vocabulary are not disjoint in a strict chemical sense (polyoxides and polyethers, for instance), so a class holdout does not necessarily remove all related bonding motifs. Its difficulty is not a mathematical lower bound on every possible chemistry holdout.

**P4, high-**$T_{g}$ **extrapolation.** The model is trained on the lower 80 % of the $T_{g}$ distribution ($T_{g}<524.1$ K) and tested on the upper quintile (n=1480), which reproduces the search for heat-resistant candidates. This is conceptually the property-based extrapolation split distributed with PolyMetriX [25]; it is included so that chemistry-based and property-based extrapolation can be compared on one axis.

An unsupervised alternative to P3 is reported in Section 3.8: whole-cluster five-fold holdout on Butina clusters [35] of the 2048-bit Morgan fingerprints at Tanimoto-distance cutoffs 0.5, 0.65 and 0.8, with greedy size balancing across folds. This is the same construction used for the external band-gap set, and it separates the effect of holding out a region of structure space from the effect of holding out a curator-named class.

### 2.5. Models and Nested Tuning

Five transparent baselines were used unchanged across all protocols and both streams: a mean predictor, ridge regression with generalised cross-validated regularisation, distance-weighted k-nearest neighbours (k=5), a random forest (300 trees), and histogram gradient boosting (400 iterations), all from scikit-learn [36]. Ridge and kNN operate on features standardised within the fold. No hyperparameter of these five was tuned per protocol; the intent is that any difference between protocols is attributable to the partition rather than to model selection. The mean predictor is excluded from all reported averages and retained only as a degeneracy check.

The capacity control of Section 3.3 does tune, and does so in a nested loop: a gradient booster is selected over a three-configuration grid (differing in maximum leaf nodes, learning rate, minimum leaf size and $l_{2}$ regularisation) on an inner split of each *training* fold, using the fused stream A ⊕ B feature set. The outer test fold is never seen by the inner loop, the preprocessing pipeline or the model-selection criterion.

### 2.6. Reliability and Decision Read-Outs

For every fold we record $R^{2}$, MAE, root mean square error (RMSE), Spearman ρ and the signed bias. Because a $T_{g}$ model is normally used to select candidates rather than to report a number, two decision-level read-outs are added:

- **Screening balanced accuracy.** Predictions are thresholded at $T_{g}\geq 450$ K, a representative cut for a heat-resistant candidate, and the balanced accuracy of the resulting binary decision is reported. Balanced accuracy is used because the positive rate varies strongly between held-out classes.
- **Top-decile enrichment.** For a test fold of size n, let k=max(1,round(0.10 n)), let the observed top decile be the k repeat units with the highest measured $T_{g}$ in that fold, let m be the size of that set, and let H be the number of the k highest-predicted repeat units that fall inside it. Enrichment is
  $$EF=\frac{H/k}{m/n}$$

The observed top set is taken by rank, not by a value threshold: it is exactly the k repeat units with the highest measured $T_{g}$, with ties broken deterministically by index, so m=k in this implementation and $EF=nH/k^{2}$. Normalising by the realised prevalence m/n rather than by the nominal 0.10 is what makes the expected enrichment of a random ranking equal to 1 exactly in finite samples: k is an integer, so k/n only approximates 0.10, and a fixed denominator would leave the no-skill level at (k/n)/0.10 instead. An enrichment of 1 is therefore the exact no-skill expectation. Because k is small for the smaller held-out classes (k≈4 at n=40), enrichment is reported with a percentile bootstrap 95 % confidence interval (5000 resamples) and is accompanied by precision@k, recall@k, the hit count and average precision, all of which are in protocol_scores.csv. For P1–P3 the resampling unit matches the design. Under P1 the fold labels have no identity across the three shuffled five-fold repetitions: averaged over the 15 same-numbered cross-repetition pairs, folds carrying the same number share a mean Jaccard of 0.107 of their members, which is what two unrelated random fifths would share (0.111). The bootstrap unit is therefore the polymer: polymers are resampled and enrichment is recomputed within every realised test partition before aggregation. P2 resamples its 5 scaffold folds and P3 its 17 held-out classes. P4 consists of a single deterministic property-extrapolation test set, so there is no fold to resample; its interval resamples the 1480 candidates within that held-out set. Every interval recomputes the four-baseline mean enrichment on each resample. Fold accounting is set out in full in fold_accounting_audit.md. For example, with N = 103 test polymers and k = 10, the realised top-set prevalence is 10/103. If 4 of the 10 selected candidates are true top-set members, EF = (4/10)/(10/103) = 4.12. Random selection has an expected 100/103 hits, giving expected EF = 1 despite the prevalence being slightly below 0.10.

Two aggregations of a heterogeneous holdout are reported and are not interchangeable. The *macro* score is the unweighted mean over the 17 class-holdout folds; the *pooled* score concatenates all held-out predictions and computes one score. Pooling restores the large between-class variance of $T_{g}$ to the denominator of $R^{2}$, so it answers a different question, as Section 3.2 quantifies. Unless stated otherwise, class-holdout numbers in this paper are macro.

The applicability-domain diagnostic is the maximum Tanimoto similarity, on 2048-bit Morgan fingerprints, between each held-out repeat unit and the training partition of its own fold.

### 2.7. Between-Source Variability Reference

For polymers reported by two or more independent sources, the curated table carries the individual source values. We use them to quantify how far independent literature reports of the same repeat unit disagree.

Let $v_{i1},\ldots ,v_{in_{i}}$ be the reported values for polymer i. We report the mean absolute pairwise difference, and—because the curated label is the median over sources—the leave-one-source-out discrepancy $n_{i}^{-1}\sum_{j}\left|v_{ij}-m_{i,-j}\right|$, where $m_{i,-j}$ is the median of the reports for polymer i with report j removed. This is the quantity that matches how the label was constructed. Uncertainty is a hierarchical bootstrap over polymers (5000 resamples) in which a polymer is resampled as a unit with all of its reports, because a polymer with ten reports contributes forty-five correlated pairwise differences.

This quantity is a *variability reference*, not an attainable error floor, and the distinction is not cosmetic. Identifying the two-point sample standard deviation with an attainable MAE through $E[s]=\sigma \sqrt{2/\pi }$ would fail here for three reasons: $E[s]=c_{4}(n)\sigma$ depends on n, and only 132 of the 279 multiply reported polymers have exactly two sources; the model is trained and scored against the median over sources rather than against a single laboratory report; and the dispersion between reports mixes measurement error with differences in molar mass, tacticity, thermal history, specimen preparation and measurement method. We therefore avoid the word *floor*: this dispersion is a reference scale for judging error magnitudes, not a bound, and no claim is made that a model attaining this error would be optimal. In the vocabulary of uncertainty classification [37], the specimen-to-specimen part of the spread is irreducible for a model with no access to processing variables and the measurement-method part is in principle reducible by better reporting; the curated table does not separate the two. The derivation and its assumptions are given in reproducibility_reference_method.md.

### 2.8. Group-Contribution Prior and Its Integration

The prior has the additive Van Krevelen form [1]

(1) $$g(x)\;=\;\frac{Y_{g}(x)}{M(x)},\;\;Y_{g}(x)\;=\;\sum_{i}n_{i}(x)\;Y_{i}$$

where $n_{i}(x)$ counts occurrences of structural group i in the repeat unit, M(x) is the repeat-unit molar mass, and $Y_{i}$ are molar glass-transition increments. Thirty-five groups defined using the SMILES arbitrary target specification (SMARTS) language spanning the backbone and pendant chemistry present in the dataset were used. The increments are not quoted from a handbook. They are estimated on the training partition of each fold by non-negative least squares [38] applied to the linear system $NY=T_{g}⊙M$ implied by Equation (1). This choice makes calibration quality a controlled experimental factor rather than an unknown property of a printed table. Fitting the increments inside each training fold gives the prior a deliberately favourable calibration on the training chemistry of that fold and removes the uncertainty associated with transferring an externally tabulated increment set; it is not a claim that fitted increments predict unseen chemistries better than published tabulated ones, which this study does not test (Section 4.5). Minimising the sum of $(N_{i}Y\;-\;M_{i}T_{g,i}{)}^{2}$ is equivalent to minimising the sum of $M_{i}^{2}(g_{i}\;-\;T_{g,i}{)}^{2}$. Thus the original fit weights squared Tg errors by repeat-unit molar mass squared; this is a modelling choice inherited from fitting the molar glass-transition function, not an estimate of measurement precision. A direct Tg-space non-negative least-squares sensitivity fit is reported in Supplementary Table S8. The integration factorial retains the original prior and remains conditional on that weighting choice. The direct Tg-space fit changes the prior-only macro R^2^/MAE from 0.670/48.1 K to 0.692/46.7 K under P1, and from −0.047/57.0 K to 0.017/55.1 K under P3. The prior remains coarse under either fitting criterion; integration modes were not rerun for this fitting-space comparison.

Provenance of the group dictionary. The 35-group list was written after inspecting which chemistries occur in the full dataset. Although Tg values were not used to choose groups and increments are fitted within each training fold, this is a dataset-informed representation rather than a fully external prior. Fold-internal coefficient fitting does not remove that design dependence. We limit the integration claim and report a targeted sensitivity experiment omitting the Si and P elemental groups in the two inorganic-backbone class holdouts (Supplementary Table S7). This removes those explicit group counts, but does not make the remaining dictionary or stream A independent of the dataset.

Groups are allowed to overlap and a single atom may be counted by more than one group: an aromatic carbon contributes to arom_C and, if it belongs to a benzene ring, that ring also contributes to phenylene. No precedence rule resolves the overlap, which can introduce collinearity into the design matrix, so coefficients need not be uniquely identifiable. Non-negative least squares restricts coefficient signs but does not guarantee identifiability or stability under collinearity. A zero increment therefore means “not needed given the other groups”, not “chemically irrelevant”. All 35 groups have non-zero support in every one of the 22 outer folds of the prior analysis, 24 receive a non-zero increment in every one of them, and 2 (urea, anhydride) are present in fewer than 1 % of repeat units. Zero support would make an increment unidentifiable and the implementation would assign zero, but that case did not occur in these 22 folds and cannot explain the observed degradation. Per-group SMARTS, chemical meaning, overlap rule, support count, fold-wise non-zero count and fitted increment are in Supplementary Table S1.

Three strategies compare omission of the empirical prior (L0), a quadratic prior penalty (L1) and an additional prior input (L3):

The prior is not used, so $L=n^{-1}\sum_{i}(f_{\theta }(x_{i})-y_{i}{)}^{2}$.

The prior is enforced as an additive penalty,

(2) $$L_{L1}(\theta )=\frac{1}{n}\sum_{i}(f_{\theta }(x_{i})-y_{i}{)}^{2}+\lambda \;\frac{1}{n}\sum_{i}(f_{\theta }(x_{i})-g(x_{i}){)}^{2}$$

The factorial uses λ = 1, with the weight swept in Section 3.10. For squared losses, $(f\;-\;y{)}^{2}\;+\;\lambda (f\;-\;g{)}^{2}\;=\;(1\;+\;\lambda )[f\;-\;\frac{y\;+\;\lambda g}{1\;+\;\lambda }{]}^{2}\;+\;\frac{\lambda (y\;-\;g{)}^{2}}{1\;+\;\lambda }.$ The last term is independent of the network prediction f. L1 fits a target shrunk towards g, with a common loss scale; it is not a physics-informed neural network (PINN) enforcing a physical differential equation [27,28]. Gradient disagreement describes the two components of this empirical objective.

g(x) is appended to the input vector and the objective remains the plain data loss, so the model is free to use or ignore it.

Calibration quality is varied by injecting a controlled additive bias, $g_{\delta }(x)=g(x)+\delta \;\sigma_{y,train}$ with δ∈{0,0.25,0.5,1.0}. The bias is scaled by the target standard deviation estimated exclusively from the outer training partition of each fold, $\sigma_{y,train}=SD(y_{outer\;train})$; no target statistic from the outer test partition enters the definition of the perturbation. The scale is a design constant rather than a fitted parameter, so this choice cannot leak predictive information, but a paper about evaluation leakage should not require the reader to take that on trust. Across the 22 outer folds $\sigma_{y,train}$ ranges from 104.2 to 115.3 K against 112.7 K for the whole dataset, a spread of −7.6 % to +2.3 %; per-fold values are in prior_bias_train_only_audit.csv. δ=0 is the in-fold calibrated prior; δ=1 represents a prior transferred from a population whose mean $T_{g}$ differs by one $\sigma_{y,train}$, a realistic failure mode when handbook increments are applied outside the chemistry on which they were derived.

The base learner for this comparison must be differentiable, so a compact fully connected network (two hidden layers of 64 rectified-linear units, Adam, 150 epochs, minibatch size 256, learning rate $3\times 10^{-3}$) was implemented directly in NumPy, operating on stream A. Inputs and target are standardised on the training fold, and g is standardised with the *target* scaler so that λ is dimensionless. Each condition is repeated with three random initialisations. The factorial crosses integration strategy × bias δ × protocol (random five-fold, and holdout of each of the 17 polymer classes with n≥40, the same classes used for P3 throughout), and effects are reported as the paired difference $\Delta R^{2}$ against the L0 run on the same fold.

For L1 we record the cosine between the minibatch weight gradients of the data and prior terms over the final 30% of training steps. The fraction of negative cosines is a descriptive gradient-conflict rate [29]. In view of the quadratic identity above, it does not establish a distinct physical mechanism.

### 2.9. Statistical Unit, Intervals and Multiplicity

The prior-integration factorial produces three paired runs per fold, one per random initialisation. These are *not* independent observations: three seeds on one fold share the same training and test data, and different folds of the same protocol share most of their training data. A seed-level analysis of variance is therefore inappropriate: treating the 15 random-split runs as independent inflates the denominator degrees of freedom and produces p-values that reflect that inflation rather than the effect.

The statistical unit used throughout is therefore the **fold**. Seeds are averaged within a fold before any comparison, leaving n=5 paired observations per cell under P1 and n=17 under P3. We report, in this order: the effect size (mean paired $\Delta R^{2}$); a percentile bootstrap 95 % confidence interval resampling folds (5000 resamples) [39]; a two-sided sign-flip permutation test over folds; and the fraction of folds in which the sign is consistent. Holm’s step-down correction [40] is applied within each protocol’s family of eight contrasts (2 integration strategies × 4 bias levels).

The power of the two protocols differs sharply, and this must be read with the results. A two-sided sign-flip permutation test on n paired folds has a minimum attainable p-value of $2/2^{n}$. Under P1, with five folds, that floor is 0.0625: **no random-split contrast can attain a nominal Holm-adjusted** p<0.05**, whatever its effect size**, and none is claimed. Under P3 the floor is $2/2^{17}=1.5\times 10^{-5}$, so class-holdout contrasts can be tested, and two of them reach a nominal Holm-adjusted p<0.05. We call these nominal rather than significant throughout: the 17 leave-one-class-out fits share most of their training observations, so neither the permutation p-values nor the class-resampling intervals are exact repeated-sampling inference (Section 4.5). The factorial uses all 17 held-out classes rather than a subset: with five folds the permutation floor would be 0.0625 under both protocols, and a subset of five is in any case not a representative sample of the 17 (Section 3.9). A linear mixed model dR2 ∼ integration × bias with a random intercept for fold was fitted for completeness and is reported in mixed_effects_diagnostics.txt; under P1 its p-values rest on asymptotics that five groups do not support and are not used for inference.

For the similarity–error regression, the unit is an individual test prediction. The archived P1–P3 intervals use a percentile-t wild cluster bootstrap with Rademacher signs drawn once per outer fold, with 2000 draws. Five folds in P1 and P2 permit only 2^5^ = 32 distinct sign vectors; 2000 draws repeat this finite support and do not add independent information. We exhaustively enumerated all 32 vectors using the discrete inverse empirical distribution for the 2.5% and 97.5% quantiles (Supplementary Table S9). The endpoints agree with the reported rounded intervals. These are coarse, approximate uncertainty summaries, not evidence of precise 95% repeated-sampling coverage. P3 also has dependence through overlapping training sets. P4 has only one outer fold, so its heteroskedasticity-consistent type-3 (HC3) interval describes variation among its 1480 test candidates without partition-to-partition uncertainty.

### 2.10. Uncertainty Quantification and Calibration

Two interval constructions are compared under all four protocols.

**Uncalibrated tree dispersion.** A 300-tree random forest supplies a per-candidate spread from the dispersion of its individual tree predictions, and symmetric intervals μ±1.645 s are formed, where s is that spread and μ the ensemble mean. The spread of a forest’s trees is not a posterior predictive distribution, and 1.645 is the Gaussian quantile rather than a calibrated one, so these are not nominal 90 % prediction intervals. They are reported as an *uncalibrated tree-dispersion heuristic*, which is how such spreads are used in practice.

**Split conformal.** We use inductive (split) conformal regression [41,42,43]. Each outer training partition is split into a proper training set and a disjoint calibration set (20 % of the training partition). The model is fitted on the proper training set only; the conformity score is the absolute residual on the calibration set; and the interval half-width is computed at quantile level min{1, ceil[(m + 1)(1−α)]/m}, with m calibration observations and α = 0.10. The archived NumPy implementation uses linear interpolation at this level. A normalised variant divides the residual by the tree spread before taking the quantile; its coverage and width are reported in Supplementary Table S5 rather than in the main text, because it behaves like the unnormalised version wherever the two can be compared and adds a second uncalibrated quantity to interpret. The calibration set is disjoint from the outer test fold in every case. For the class-holdout protocol the calibration set contains no member of the withheld class and therefore remains drawn from the training chemistries, whereas the outer test set consists entirely of the unseen class. That lack of exchangeability between calibration and test data is deliberate: it is precisely the distribution shift whose effect on conformal coverage is measured here, and not a defect of the construction. Split conformal guarantees marginal coverage under exchangeability of calibration and test data; chemistry and property holdouts violate that assumption by construction [44], and marginal validity does not imply conditional validity in any case [45], which is why coverage is reported both marginally and conditionally on structural similarity. The archived implementation evaluates the adjusted quantile using NumPy linear interpolation rather than selecting the exact order statistic; the results are therefore empirical coverage of this implementation, not a proof of an exact finite-sample guarantee.

We report empirical coverage, mean interval width, coverage conditional on the maximum Tanimoto stratum, and the correlation between the reported spread and the realised absolute error, protocol by protocol. For conditional coverage, the archived calculation includes a fold–stratum cell only if it contains at least 10 test polymers. The conditional-coverage results in Section 3.14 state the contributing fold counts and included sample totals; strata can therefore have different denominators.

### 2.11. Abstention with a Prospectively Selected Threshold

Declining to predict on part of the input space is selective prediction [46]; the question here is whether the abstention rule can be chosen without seeing the errors it is meant to avoid. Held-out candidates are ranked by their maximum Tanimoto similarity to the training partition and only those above a threshold are scored. Two analyses are reported and must not be confused.

**Retrospective risk–coverage.** The threshold is set by sweeping coverage from 0.10 to 1.00 on the *observed* holdout. This is a descriptive curve, not a deployable gate, and is labelled retrospective everywhere it appears.

**Nested threshold selection.** For P1 this analysis uses only the first prespecified five-fold repetition, matching the fold-level similarity analyses and avoiding counting the same polymers three times as nominally separate outer units; with P2, P3 and P4 that gives 5 + 5 + 17 + 1 = 28 outer folds. For each of them the threshold is selected entirely inside that fold’s training partition, then frozen and applied to the outer test fold. The inner construction is identical for all four protocols. The training partition is split by GroupKFold into four parts on the *scaffold key*, and the first of those four is taken as the inner holdout, so it consists of whole scaffold groups and is about a quarter of the training partition; GroupKFold does not shuffle, so this split is a deterministic function of the scaffold keys and involves no seed. A model is fitted on the remaining three quarters and predicts the inner holdout, and each inner-holdout point is given its maximum Tanimoto similarity to that inner training part. Candidate cuts are the grid 0.20,0.21,…,0.95. A cut is admissible if it retains at least 20 % of the inner holdout and at least 20 points; among admissible cuts whose selective MAE on the inner holdout is at most 30 K—a target fixed a priori—the one retaining the *most* candidates is chosen, that is, the smallest admissible cut meeting the target. If no cut reaches the target, the admissible cut with the lowest inner selective MAE is taken instead and the fold is flagged; this did not occur in any of the 28 folds. One inner holdout yields one threshold: there is no averaging over inner folds, repetitions or seeds. The model is then refitted on the whole training partition and the frozen threshold applied to the outer test set. We report the selected threshold, the realised outer coverage, the selective MAE and the full-fold MAE. Only this analysis licences a claim about what an abstention gate would do prospectively. Per-fold thresholds and coverages are in selective_prediction.csv.

### 2.12. External Replication Set

To test whether the protocol effect is a property of $T_{g}$, of experimental data, or of literature-derived tables in general, a matched evaluation battery was repeated on a second openly accessible set: the DFT-computed chain band gap of polymers distributed in the polyVERSE repository [32]. Of 4209 records, 81 were rejected for failing to parse or for not carrying exactly two polymerisation points and one canonical duplicate was collapsed, leaving 4127 structures with band gaps in 0.07–9.84 eV (mean 4.49, standard deviation 1.44 eV). The target is a consistently computed DFT reference produced under a common workflow, so it carries no inter-laboratory experimental scatter; it is not error-free, and the limitations of Section 4.5 state what it does carry instead. The set has no curator-assigned polymer classes, so chemistry families were defined by Butina clustering [35] of the 2048-bit Morgan fingerprints at a Tanimoto-distance cutoff of 0.65, giving 573 clusters, the largest holding 13.1 % of the data; whole clusters were assigned to five folds by greedy size balancing and held out in turn. Because the band-gap dataset carries no curator-assigned polymer classes, its chemistry-holdout analogue is this cluster-grouped five-fold construction rather than the named-class P3 protocol, which has no counterpart here. Only stream B is available for this set, since the published polymer-aware descriptors accompany the $T_{g}$ table alone. MAE is additionally reported normalised by the target standard deviation so that the two studies can be compared on one axis.

### 2.13. Few-Shot Calibration of a Withheld Class

For nine polymer classes spanning the full range of class-holdout difficulty, k∈{0,5,10,25,50,100} members of the withheld class were drawn at random, added to the training set, and the model scored on the remaining members; each k>0 was repeated with five independent draws. Cells in which k would leave fewer than about a third of the class as test data are not reported. The model is histogram gradient boosting on stream B, unchanged from the main study. These curves evaluate random calibration sampling; whether a designed calibration set performs better requires a separate comparison.

Retraining is an expensive way to use k labels, and it is not obvious that the gain comes from re-learning a structure–property relation rather than from removing a class-level intercept. Three cheaper baselines therefore consume exactly the same k labels and are compared against retraining:

- Mean-offset correction, using the same sign convention as the other analyses: b_k = (1/k)Σ_i(ŷ_i − y_i) and ŷ_cal = ŷ − b_k. Thus an estimated bias of +91 K lowers every prediction by 91 K. This is algebraically identical to adding the experiment-minus-prediction residual in the original implementation; no numerical results change.
- **Linear recalibration**, ${\hat{y}}_{cal}=a\hat{y}+b$ with a,b from least squares on the same k members.
- **No correction**, the zero-shot model, as a control.

### 2.14. Stronger Models, Learning Curves and the Penalty-Weight Sweep

Three controls guard against the objection that the protocol gap is an artefact of deliberately weak or under-trained baselines. First, the nested-tuned booster of Section 2.5 on the fused stream A ⊕ B feature set. Second, a three-way average of that tuned booster, a 300-tree random forest and a ridge model, evaluated as a simple ensemble. Third, learning curves measured by subsampling each training fold to 10–100 % of its size with three subsampling repetitions below 100% and one full-data fit at 100%. Finally, the penalty weight of the loss-constrained prior is swept over λ∈{0.1,0.3,1,3,10} crossed with calibration bias δ∈{0,0.5}, three seeds, and both the random and class-holdout protocols, with λ=0 recovering the L0 baseline used for the paired comparison. The statistical unit for the sweep is again the fold. The λ = 1 entries are reused directly from the factorial, with identical folds, seeds and preprocessing; they are not an independent replication.

### 2.15. Software, Reproducibility and Use of AI Assistance

The original analyses used Python 3.12.10 with NumPy 2.5.1, pandas 3.0.5, scikit-learn [36], SciPy 1.18.0, statsmodels, matplotlib 3.11.1 and RDKit 2026.03.5 [33]. The exact resolved versions of every package in the environment that produced the reported numbers are pinned in requirements-lock.txt in the original_reproduction/ folder of Reproducibility_Archive.zip; the software versions, including those of scikit-learn and statsmodels, are provided in that file. Revision analyses are separately archived with their script and runtime versions in revision_analyses. They use the original cached feature matrices and model and sampling settings; full-dictionary and full-data controls are compared with archived values before interpretation.

Every random seed is fixed at the module level (SEED = 20260805) and recorded; the seed governs fold assignment, forest and booster construction, network initialisation, subsampling draws, few-shot calibration draws and every bootstrap and permutation resample. No analysis was retried with a different seed after its result was seen, and no result reported here was selected from among several runs. The analysis scripts, the curation log with the input file digest, the leakage report, the per-fold score tables and the out-of-fold predictions behind every figure are provided as Supplementary Material, together with a source map covering all 16 figures (figure_source_map.json in the archive root). No result in this paper depends on data that are not openly redistributable.

An AI coding assistant assisted with manuscript revision, numerical auditing and implementation of the supplementary sensitivity analyses. The revision archive contains the executed scripts and derived numerical outputs. AI assistance does not replace author responsibility for scientific interpretation, code verification and final manuscript approval.

## 3. Results

**Figure 1:**
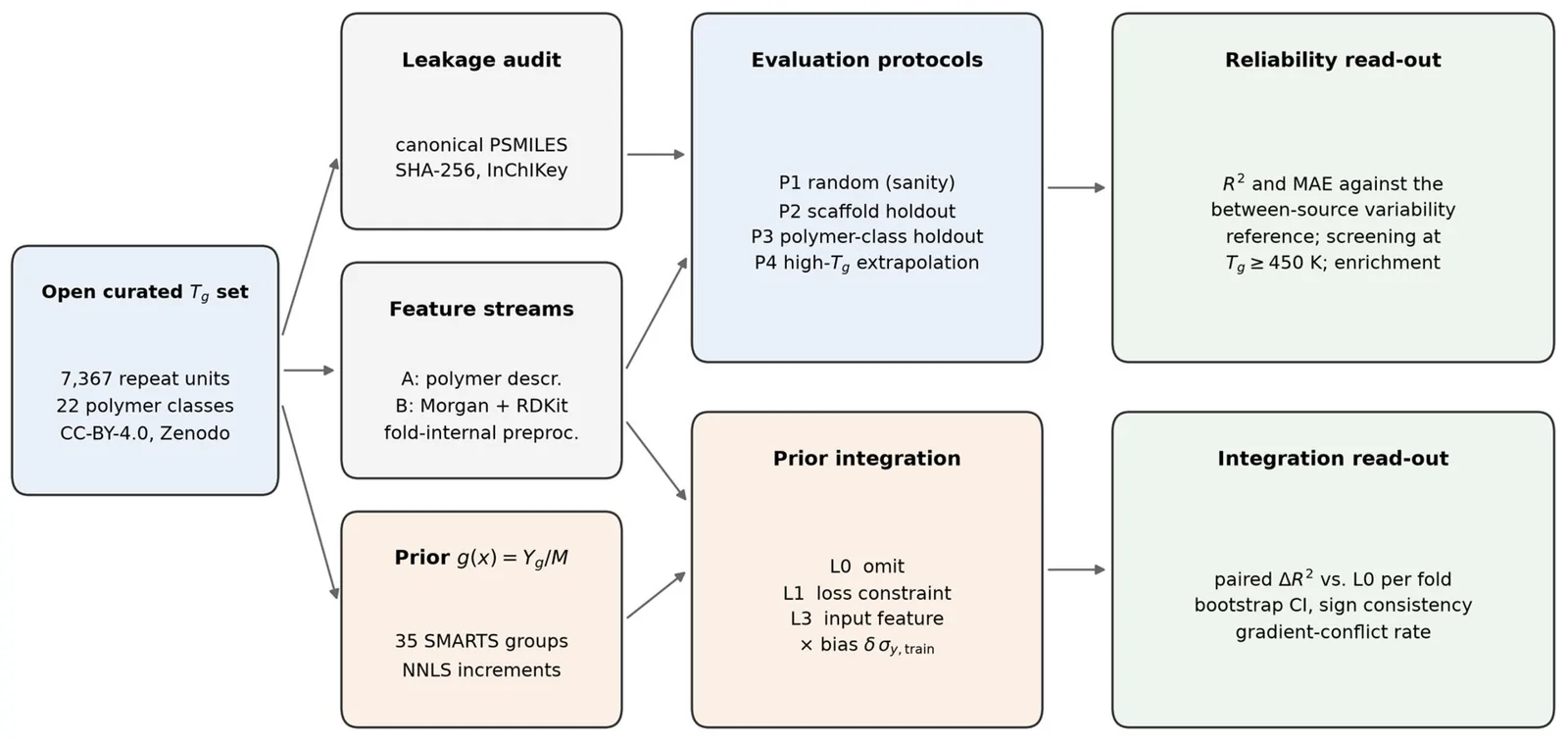
Study design. A single openly licenced curated $T_{g}$ dataset feeds a leakage audit, two independent feature streams and a group-contribution prior. Four evaluation protocols and three prior-integration strategies are applied to identical data with identical models. All preprocessing is fitted inside the training partition of each outer fold.

The results first compare the four prediction settings, then examine which polymer families fail and whether similarity, uncertainty intervals or a small calibration set help explain or reduce those errors. Figure 1 summarises the data and analysis design.

### 3.1. Curation and Leakage Audit

All 7367 records parsed, all carried exactly two polymerisation points, and all $T_{g}$ values fell inside the admissible window, so no record was discarded on those grounds (Table 1). The identity audit is more informative. The 7367 PSMILES strings are distinct as written and remain distinct after RDKit canonicalisation, so the published table contains no duplicate structures at the level at which it is normally used. Removing stereochemistry merges one pair, and the standard InChIKey of the methyl-capped repeat unit resolves only 7354 distinct species—13 collisions. The residual redundancy is therefore real but small (0.18 % of records), and stereoisomer pairs are grouped for scaffold holdout, but the archived partitions do not enforce grouping of all methyl-capped InChIKey collisions. The canonical-structure audit rules out exact duplicate repeat units across train and test [18], but does not exclude cross-partition identity overlap under the looser keys. The structural-neighbour analysis below therefore complements rather than replaces this limited duplicate check.

Scaffold grouping produced 3434 groups over 7367 records; the largest group holds 9.5 % of the data, so grouped five-fold partitioning is well conditioned. The in-fold variance filter drops one constant column from stream A in every fold, leaving 99 descriptors; stream B supplies 1034 features. Content hashes of the training and test structures overlap in zero folds.

### 3.2. The Evaluation Protocol, Not the Model, Sets the Reported Reliability

**Figure 2:**
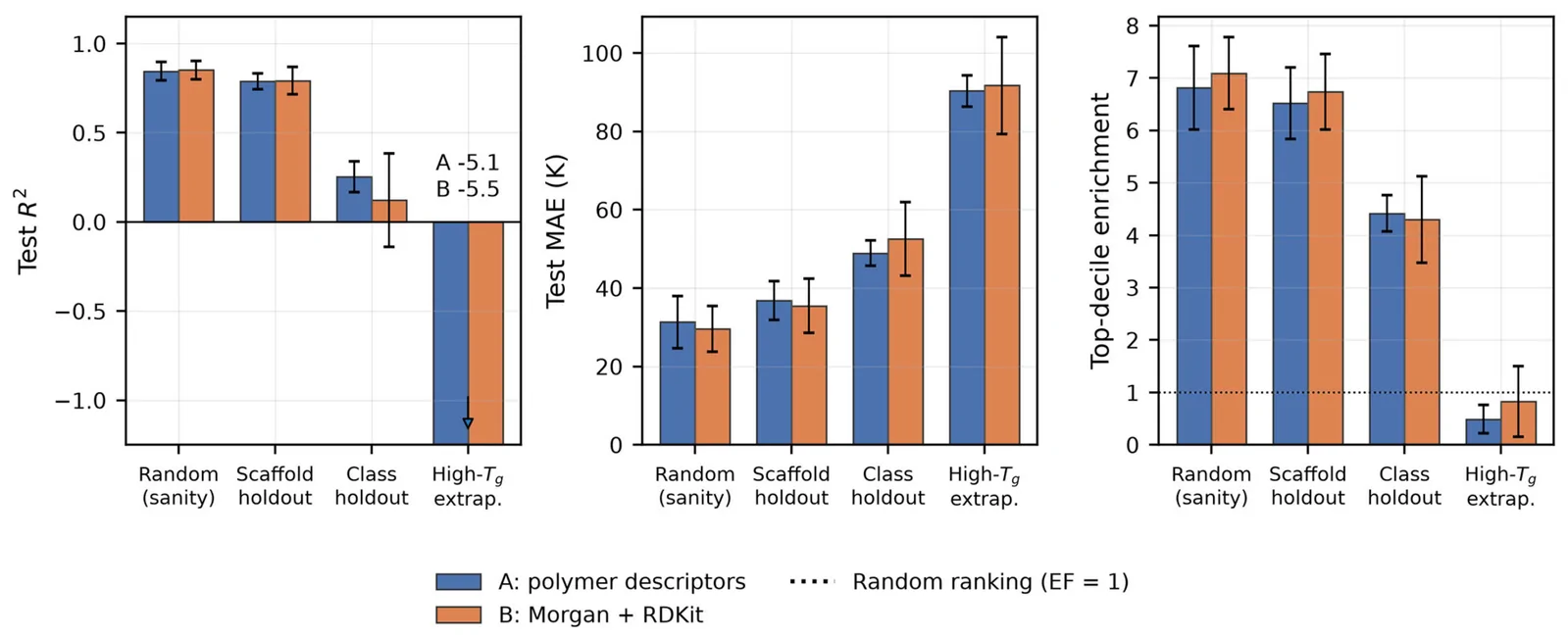
Reported reliability as a function of evaluation protocol, averaged over the four non-degenerate baselines and both feature streams. Error bars are standard deviations across the four non-degenerate baseline models, each first averaged over the folds of its protocol; they show model-to-model dispersion rather than fold-to-fold uncertainty. No fold-level interval is given here or in Table 2 for $R^{2}$ and MAE; protocol-appropriate bootstrap intervals are reported for enrichment in Table 2, and for both decision-level read-outs in Section 3.6. Source data: Supplementary Table S4. (**Left**): test $R^{2}$, clipped at *−1.25* so that the informative range stays legible; the high-$T_{g}$ bars reach *−5.1* and *−5.5*. (**Centre**): test MAE. (**Right**): top-decile enrichment, with the no-skill level at 1. Class-holdout entries are macro means over 17 folds.

Table 2 and Figure 2 give the central result. Averaged over the four non-degenerate baselines, random five-fold cross-validation returns $R^{2}=0.844$ (stream A) and 0.852 (stream B) with MAE 31.3 and 29.6 K—squarely inside the range reported in the recent $T_{g}$ literature [9,10,11]—providing a useful calibration of these deliberately untuned baselines against published practice. Holding out scaffold groups costs little: $R^{2}$ falls to 0.790/0.792 and MAE rises by about 6 K. Holding out an entire polymer class is a different matter: macro $R^{2}$ collapses to 0.253 and 0.121, and MAE rises to 48.9 and 52.6 K. Under high-$T_{g}$ extrapolation the mean $R^{2}$ is −5.07 and −5.47 with MAE near 91 K, worse than the spread of the held-out data itself.

The same ordering holds for a single strong model. Histogram gradient boosting on stream B scores $R^{2}=0.899\pm 0.008$ (MAE 23.9 ± 0.7 K) under random five-fold cross-validation, 0.857 ± 0.031 (28.5 ± 2.0 K) under scaffold holdout, a per-class mean of 0.355 ± 0.781 (43.3 ± 14.2 K) under class holdout, and −3.79 (79.5 K) under high-$T_{g}$ extrapolation. Because the two streams share no code path and disagree about which is better under class holdout, the ordering is consistent across these two feature streams. These four numbers are unchanged to four decimal places by moving all preprocessing inside the fold (Section 2.3).

One measurement subtlety deserves emphasis. If the predictions from all 17 class-holdout folds are pooled and a single $R^{2}$ is computed, the same model returns 0.802 with a pooled MAE of 36.6 K—apparently respectable figures. The discrepancy with the macro values of 0.355 and 43.3 K is not a contradiction: pooling restores the large *between*-class variance of $T_{g}$ to the denominator, so the pooled score mostly certifies that the model places an unseen class in roughly the right temperature region. It says almost nothing about ranking members *within* that class, which is what a screening campaign inside a new chemistry actually needs. Aggregate scores computed over heterogeneous holdouts should therefore be reported alongside, not instead of, the per-holdout distribution.

**Figure 3:**
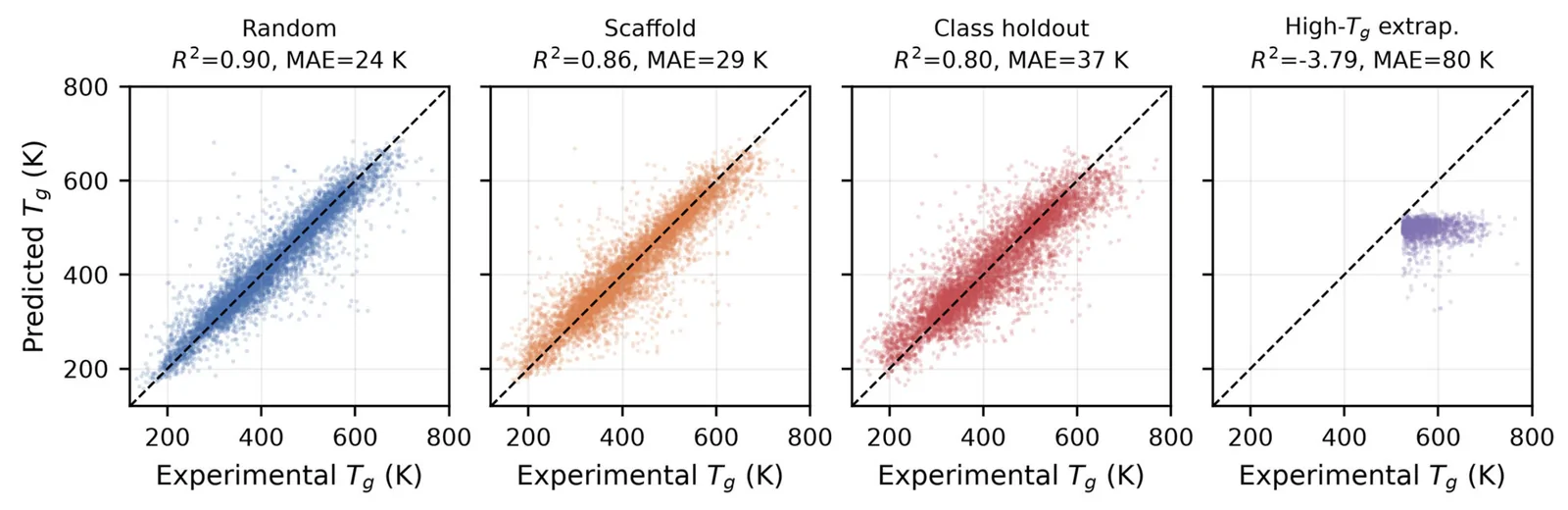
Out-of-fold parity plots for histogram gradient boosting on stream B, one panel per protocol, pooling the held-out predictions of every fold. Under high-$T_{g}$ extrapolation, the predictions collapse onto a horizontal band near the upper edge of the training range: the model reproduces the boundary of what it has seen rather than the property of what it has not. The $R^{2}$ printed on each panel is pooled, not macro.

The out-of-fold parity plots in Figure 3 show what each protocol does to the predictions themselves. Random and scaffold splitting scatter about the diagonal; class holdout widens that scatter; under high-$T_{g}$ extrapolation the predictions collapse onto a horizontal band near the top of the training range. The large negative $R^{2}$ of that protocol is a real failure but a poor summary of it: the denominator of $R^{2}$ is the variance *within* the top $T_{g}$ quintile, which is small, so the value is unstable. MAE and the ranking read-outs carry the interpretable content and are what we quote hereafter.

### 3.3. Neither a Stronger Model nor More Data from Already Represented Chemistries Closes the Gap

Two objections have to be answered before the protocol effect can be attributed to the partition. The first is that the baselines were deliberately untuned; the second is that the holdout folds are simply short of data.

**Figure 4:**
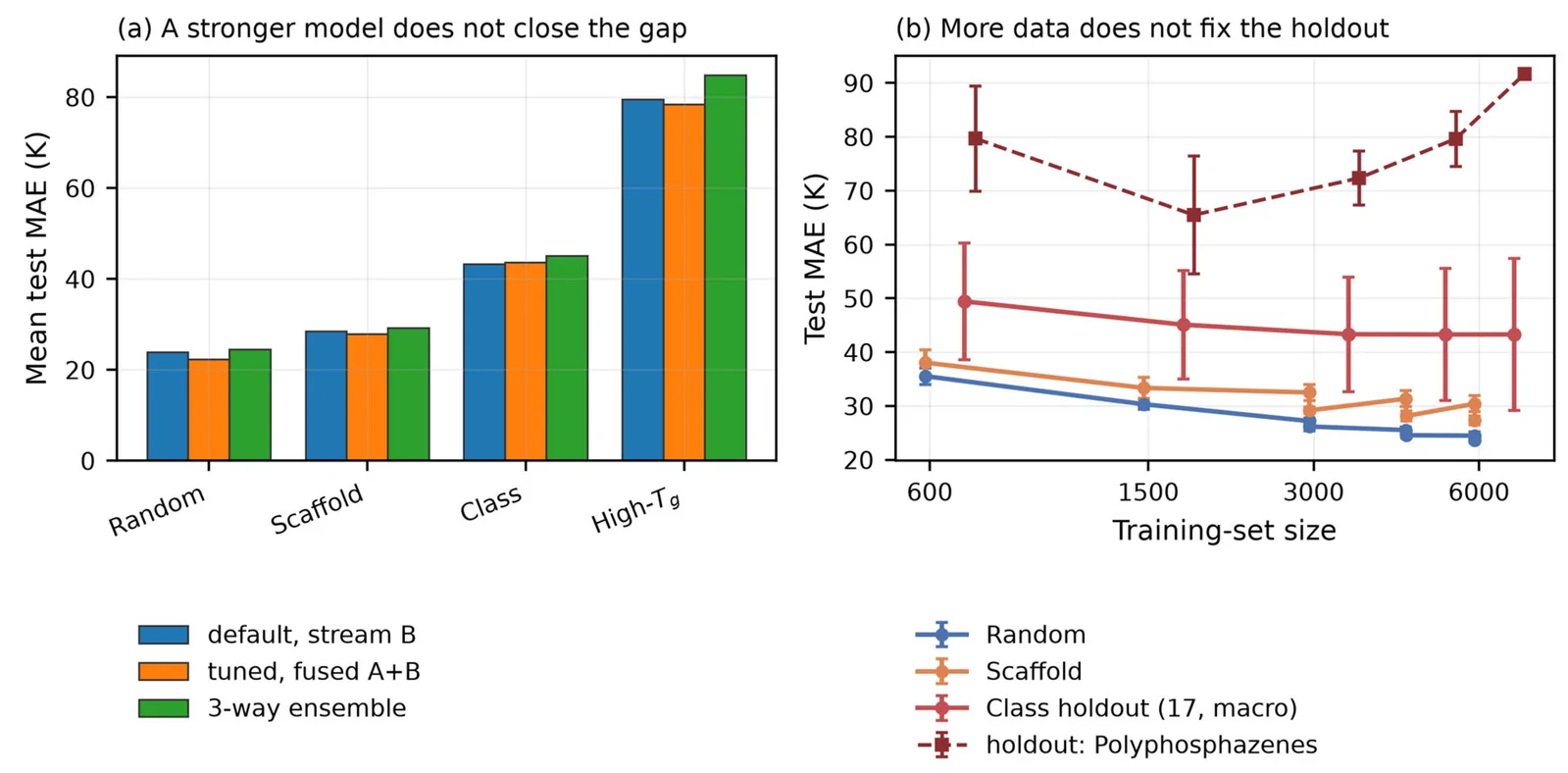
(**a**) test MAE by protocol for the default baseline (histogram gradient boosting, stream B), a nested-tuned booster on fused stream A ⊕ B features, and a three-way ensemble; macro means over folds. (**b**) learning curves for the same default model; the class-holdout curve is a macro mean over all 17 withheld classes. The interpolation protocols improve with training-set size in the usual way, while class holdout plateaus beyond half the training pool. Polyphosphazenes are shown separately because they are the only class of the 17 whose error rises as more data from the remaining, already represented chemistries is added. For random and scaffold holdout, error bars are standard deviations of the fold/repetition MAE values sharing each plotted training-set size. For the class-holdout macro curve, repetitions are first averaged within each class, and error bars show the standard deviation across the 17 classes. For the separate polyphosphazene curve, error bars below the full-data point show standard deviations over three subsampling repetitions. At the full-data fraction, each fold has one deterministic fit.

Capacity. Tuning the booster on an inner split of each training fold and giving it the fused stream A ⊕ B feature set improves the random-split MAE from 24.0 to 22.3 K, a 7 % gain, and lifts $R^{2}$ from 0.898 to 0.905. Under scaffold holdout, the same change is worth 0.6 K. Under class holdout, it is worth nothing—MAE moves from 43.3 to 43.6 K and $R^{2}$ from 0.355 to 0.361—and under high-$T_{g}$ extrapolation, it is worth 1.1 K out of 79.5. The ensemble has a higher MAE than the tuned booster in every protocol. Its scaffold-holdout R^2^ (0.858591) is slightly above the default booster (0.857392) but slightly below the tuned booster (0.859358); the ensemble and tuned booster both round to 0.859 in Table 3. Thus the ordering depends on both metric and comparator. The extra capacity therefore buys accuracy exactly where the model already works and none where it fails; the random-to-class-holdout MAE ratio in fact widens slightly, from 1.81 for the default model to 1.96 for the tuned one (Figure 4, Table 3). This capacity control uses one prespecified five-fold random repetition, giving default MAE 24.0 K; the 23.9 K headline value averages three repetitions. The P2–P4 partitions are unchanged.

**Data volume.** Subsampling each training fold gives ordinary learning curves for the interpolation protocols: random-split MAE falls monotonically from 35.5 K at 589 training points to 24.0 K at 5894, and scaffold holdout from 38.0 to 28.5 K. Class holdout does not behave this way. Averaged over all 17 withheld classes, MAE runs 49.4, 45.1, 43.3, 43.3, 43.3 K over the same range: it improves while the training pool is small and then stops improving entirely beyond half the data, whereas the random split is still gaining 0.9 K over its last quartile. Quadrupling the training data from 25 % to 100 % buys 1.8 K under class holdout against 6.4 K under random splitting.

The plateau is the general behaviour; outright reversal is not. Of the 17 classes, 16 improve between 1.4 and 16.4 K from the smallest to the largest training pool. Exactly one gets worse, and it is the most out-of-domain family identified in Section 3.5: for polyphosphazenes MAE is 79.7 ± 9.8 K at 10 % of the training pool, reaches a minimum of 65.5 ± 11.0 K at 25 %, and then rises monotonically to 72.3, 79.6 and 91.7 K, a net loss of 12.0 K. Polysiloxanes are flat within noise (49.1→44.4 K); polyimides and polyesters improve normally.

The polyphosphazene reversal should be read with its uncertainty—three repetitions per point, 107 test polymers, and standard deviations ranging from 5 to 11 K—but the trend spans four consecutive points and about 26 K. The interpretation we favour is that additional organic-backbone data sharpens a structure–property mapping that is simply wrong for a −P=N− backbone, so the model becomes more decisively incorrect. Being one class of 17 in one dataset, that is an interpretation and not a demonstrated law. Averaging only a handful of representative classes would mistake this single reversal for a general property of class holdout, which is why the curve is reported as a macro mean over all 17. What those 17 classes support is weaker and sufficient: collecting more data from chemistries that are already represented stops helping long before the gap closes, whereas measuring a few members of the absent family does help (Section 3.12). The added signed-bias analysis (Supplementary Table S10) gives mean prediction-minus-experiment biases of +77.5, +63.7, +70.3, +78.1 and +91.1 K at 10%, 25%, 50%, 75% and 100% of the training pool. The increase from 25% onwards is consistent with the proposed interpretation, but the initial decrease and the single-family design preclude a general monotonic law. The full-data point is one deterministic fit; lower fractions have three subsampling repetitions.

### 3.4. Structural Similarity Is Associated with a Limited Fraction of Prediction Error

**Figure 5:**
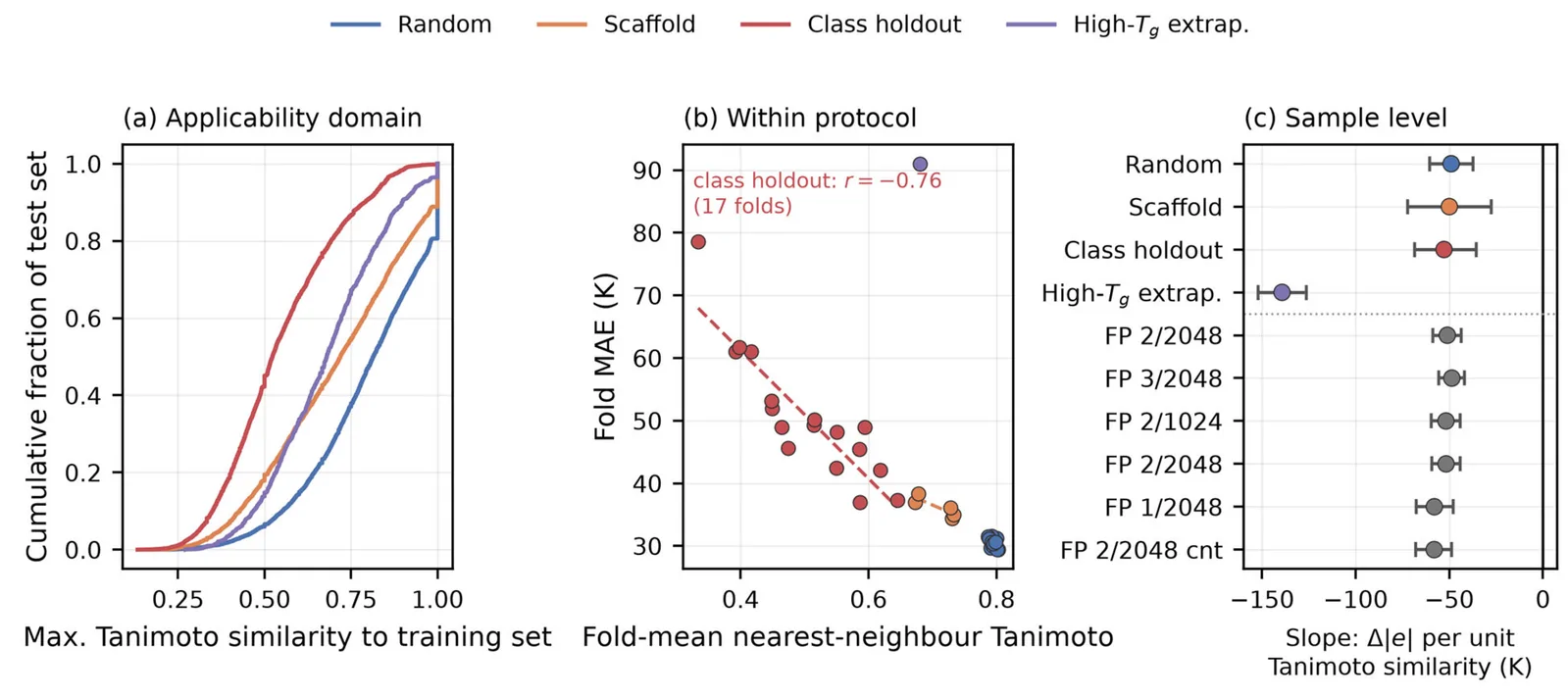
Similarity and error. (**a**) Cumulative distribution of the maximum Tanimoto similarity between each held-out repeat unit and its own training partition, by protocol, for histogram gradient boosting on stream B. (**b**) Fold-level MAE against fold-mean nearest-neighbour similarity, averaged over the four non-degenerate baselines; dashed lines are within-protocol least-squares fits, drawn only where the protocol has at least three folds. (**c**) Slope of absolute error on maximum Tanimoto similarity at the level of the individual test prediction, with 95 % confidence intervals. For P1–P3 the interval is the wild cluster bootstrap over outer folds (Rademacher weights, 2000 replications, percentile-***t***), which is wider than the asymptotic cluster-robust interval by a factor of 2.02, 2.78 and 1.10, respectively, and is the interval reported throughout. P4 has a single deterministic outer fold, so no cluster-level resampling is possible; its interval is HC3 over the 1480 candidates of that test set and carries no partition-to-partition uncertainty, which is why it is the narrowest of the four despite the largest slope. Coloured points above the dotted rule are the four protocols, each a within-protocol sample-level slope; the grey points below it are pooled estimates over the whole chemistry-holdout battery—all 5 scaffold folds and all 17 class holdouts, 14,487 predictions in 22 folds—with protocol included as a fixed effect, repeated under six Morgan fingerprint variants (radius/bits; “cnt” is the count-based fingerprint compared under the MinMax kernel). Their intervals use the same fold-cluster wild bootstrap construction as the protocol slopes above the rule and are drawn on the same scale. Because the pooled analysis contains polymers that are held out under both P2 and P3, and class holdouts that share most of their training data, these intervals are read as robustness summaries rather than exact repeated-sampling inference. For P1 and P2 the 2000 draws have only 32 distinct sign vectors. Exhaustive enumeration reproduces the rounded endpoints (Supplementary Table S9); the intervals remain coarse and approximate.

For a proposed polymer, the closest training analogue provides a simple measure of structural familiarity. Figure 5 and Table 4 test how strongly that measure is associated with error after separating the four evaluation settings.

The applicability domain itself is unambiguous. Under random splitting the mean maximum Tanimoto similarity of a held-out repeat unit to the training partition is 0.796, and 33.8 % of test polymers have a training analogue at similarity ≥0.9; only 2.0 % sit below 0.4. Scaffold holdout lowers the mean to 0.709 (22.0 % above 0.9, 6.9 % below 0.4). Class holdout lowers it to 0.502, with just 1.4 % of test polymers having a close analogue and 28.5 % falling below 0.4.

Fold-level analyses in this section use 28 fold observations rather than the 38 realised folds. The three P1 repetitions are three partitions of the same 7367 polymers, so each repetition assigns every polymer to exactly one held-out fold and any two repetitions share almost all of their training data; treating the 15 realised P1 folds as 15 independent observations would understate every interval computed from them. Only the first prespecified P1 repetition is therefore used, giving 5 + 5 + 17 + 1 = 28 fold-level observations, while all 38 realised folds remain part of the leakage audit. The accounting is set out in fold_accounting_audit.md.

A pooled descriptive correlation over the 28 fold summaries is r=−0.651, but this value is confounded by protocol and is not used for inference: protocol determines both the mean similarity and the mean error, so a pooled correlation partly measures the protocol contrast it is meant to explain. No interval is reported for it, and the claim rests on two better-controlled analyses.

*Within protocol*. Class holdout has enough folds to support a within-protocol estimate, and there the relation survives: across its 17 folds, fold-mean nearest-neighbour similarity and fold MAE are strongly negatively associated (r=−0.765, Spearman −0.86), with a slope of −121 K per unit similarity (95 % CI −189 to −53). The conventional Pearson p-value (p<0.001) is reported descriptively, because the 17 leave-one-class-out fits share most of their training observations and are therefore not 17 independent samples—the same limitation that makes the prior contrasts nominal (Section 4.5). P1 and P2 have five folds each and almost no similarity variation within them, so their within-protocol estimates are uninformative (r=+0.58 and r=−0.85; the accompanying p=0.30 and p=0.069 are likewise descriptive), and P4 is a single fold. This is a limitation of the fold-level view, not evidence for or against a causal mechanism.

*At sample level, controlling for protocol*. Regressing the absolute error of each individual test prediction on its own maximum Tanimoto similarity, with standard errors clustered on the outer fold and intervals from a wild cluster bootstrap, gives slopes of −49.0 K (95 % CI −60.6 to −37.4; n=7367) for P1, −49.9 K (−72.3 to −27.6) for P2 and −52.8 K (−68.6 to −35.6; n=7120) for P3 per unit similarity. The P1 and P2 intervals rest on five clusters each and are approximate; the 17 P3 clusters still share training data, so its interval also remains descriptive. We find no evidence that these three differ: in a mixed model pooling all protocols with a random intercept for fold, neither the P2 nor the P3 interaction with similarity is distinguishable from zero (p=0.76 and p=0.26). With five outer folds under P1 and P2 that comparison has little power, so it is read as absence of evidence for a protocol difference, not as evidence that the three slopes are equal. P4 is different, with a slope of −139.3 K (HC3 interval over the candidates of its single test set, −152.2 to −126.4), consistent with a property-range shift that structural familiarity does not repair.

Two caveats bound this. First, similarity alone explains only 6–11 % of the variance in absolute error within P1–P3 ($R^{2}=0.098$, 0.107, 0.062; 0.221 for P4): it is a real and reproducible effect, not a sufficient predictor of which individual prediction will fail. Second, the effect is not an artefact of one fingerprint. The nearest-neighbour similarity was recomputed under six Morgan fingerprint parameterisations—radius 1, 2 and 3, at 1024 and 2048 bits, with methyl-capped rather than starred repeat units, and with a count-based rather than a binary fingerprint compared under the MinMax (generalised Tanimoto) kernel—and the sample-level regression repeated for each, over the whole chemistry-holdout battery: all 5 scaffold folds and all 17 class holdouts, 14,487 predictions in 22 folds, with protocol as a fixed effect and a wild cluster bootstrap over the 22 folds. Across all six variants the point estimates stay tightly grouped, from −58.1 to −48.6 K per unit similarity. The relation is not an artefact of one Morgan fingerprint parameterisation; all six variants belong to the Morgan circular family, so this does not speak to a fundamentally different representation such as a learned embedding.

It is notable that high-$T_{g}$ extrapolation has an intermediate mean similarity (0.680) yet by far the worst accuracy, which shows that structural distance is a strong but not sufficient predictor of difficulty: a property-range shift can be damaging even when the candidate structures are chemically familiar.

### 3.5. Which Polymer Classes Fail, and in Which Direction

**Figure 6:**
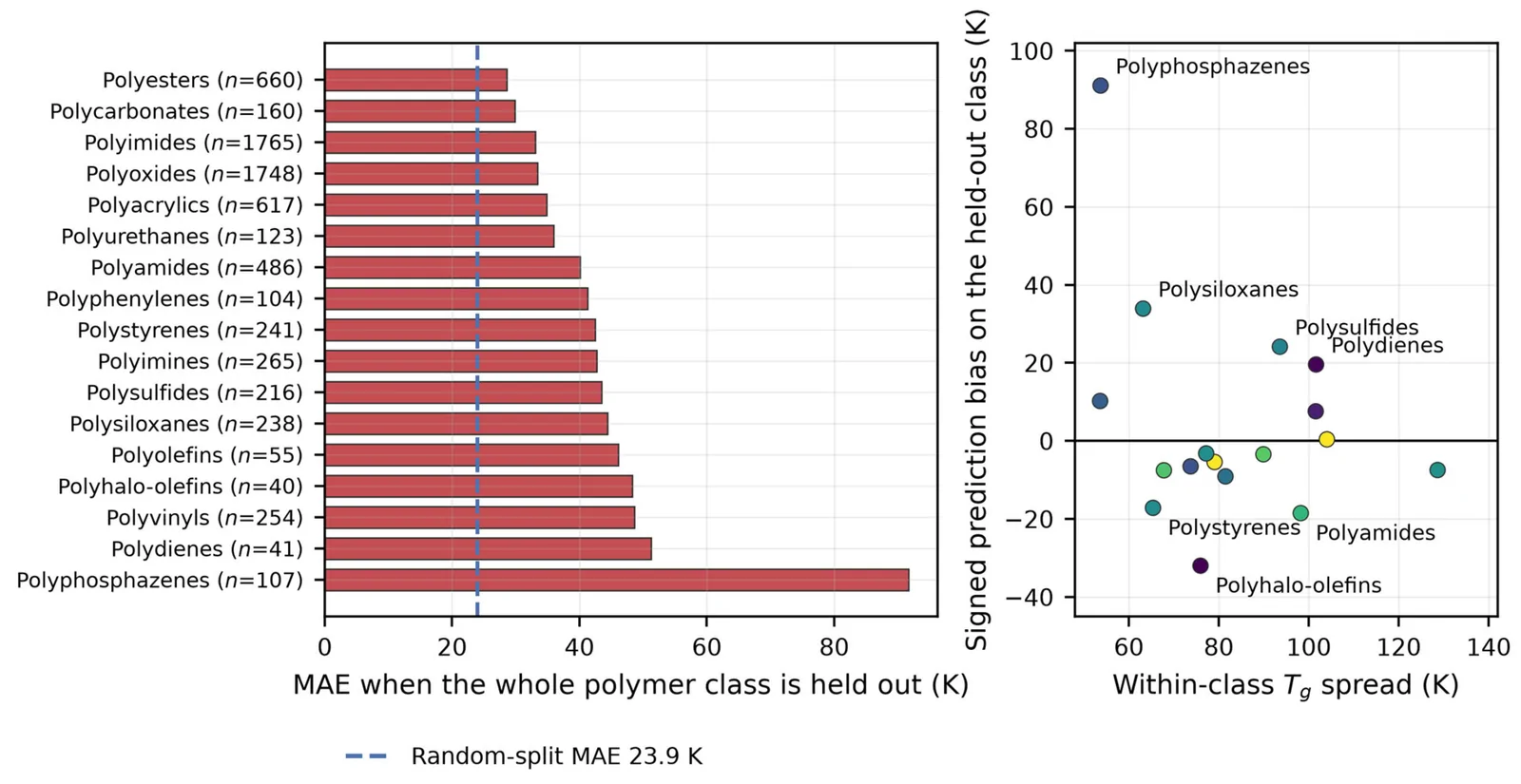
(**Left**) MAE when each of the 17 polymer classes is withheld in turn (histogram gradient boosting, stream B), against the random-split MAE of the same model. (**Right**) Signed prediction bias on the withheld class against the within-class $T_{g}$ spread; marker colour encodes ${log}_{10}n$. Only classes with an absolute signed bias of at least 15 K are labelled. The two inorganic-backbone families sit far above zero. Here, n is the number of polymers in the withheld class.

The per-class results (Table 5, Figure 6) are chemically interpretable and are the most directly actionable output of the study. The full 17-class table is Supplementary Table S3; Table 5 shows the six classes that span the behaviour.

The classes predicted best when withheld—polyesters (28.6 K), polycarbonates (29.8 K), polyimides (33.1 K), polyoxides (33.4 K)—are organic-backbone families whose bonding motifs (ester and carbonate linkages, aromatic rings, ether flexibility) recur extensively in the remaining training data. The classes predicted worst are those whose chain flexibility arises from a backbone chemistry with no analogue in the majority of the training set. Polyphosphazenes, with a −P=N− backbone and the lowest mean $T_{g}$ of any class (260 K), are predicted with MAE 91.7 K and $R^{2}=-2.56$, and are over-predicted by +91.1 K on average. Polysiloxanes, whose Si–O backbone gives exceptionally low rotational barriers, are over-predicted by +33.9 K. In both cases the model, trained overwhelmingly on conventional organic-backbone polymers, has no way to learn that the backbone itself is unusually flexible; it reads the substituents and returns an organic-backbone answer. This is an interpretation consistent with the signed biases and the training composition; it is not established by an intervention experiment.

The signed biases are as important as the magnitudes. A model that is merely noisy on an unseen class can still be useful for ranking; a model that is systematically biased by +91 K will place an entire low-$T_{g}$ family above a high-$T_{g}$ screening threshold. Polyhalo-olefins show the opposite sign (−32.0 K), and polyamides (−18.5 K) and polystyrenes (−17.2 K) are mildly under-predicted.

### 3.6. The Decision, Not the Coefficient of Determination

**Figure 7:**
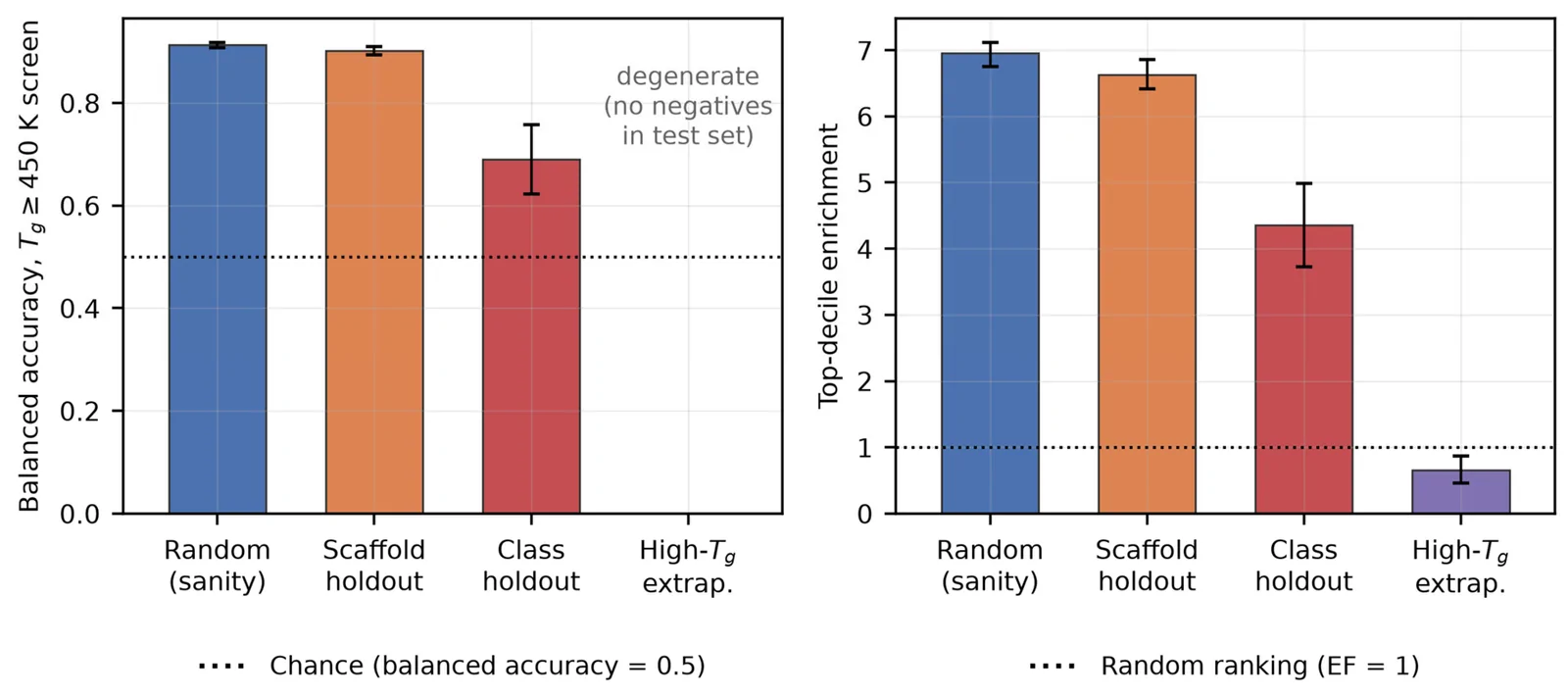
Decision-level read-outs by protocol, averaged over the four non-degenerate baselines and both feature streams. (**Left**): balanced accuracy of the $T_{g}\geq 450$ K screening decision. (**Right**): top-decile enrichment. Error bars are percentile bootstrap 95 % confidence intervals (5000 resamples), with the resampling unit matched to each design. Under P1 both read-outs use the same polymer-level bootstrap: the 7367 polymers are resampled, each drawn polymer carries its out-of-fold predictions in all three repetitions, and the statistic is recomputed inside every realised test partition before aggregation. The 15 realised (repetition, fold) partitions are 15 fifths of one fixed sample: averaged over all 105 pairs of them—including the 30 within-repetition pairs, which are disjoint by construction—the mean Jaccard is 0.079, so they are not 15 independent units and are not resampled as such. P2 resamples its 5 scaffold folds, P3 its 17 held-out classes, and P4 the candidates of its single deterministic test set, which contains no fold to resample. Dotted lines mark chance: enrichment 1 and balanced accuracy 0.5. The screening decision is degenerate under P4 because every test point lies above the threshold by construction.

A $T_{g}$ model is rarely used to report a number; it is used to decide which candidates to make. Figure 7 reports two decision-level read-outs, each with a bootstrap interval whose resampling unit matches the design (Table 2). Balanced accuracy of the $T_{g}\geq 450$ K screening decision is 0.912/0.914 under random splitting, essentially unchanged under scaffold holdout (0.902), and 0.689/0.691 under class holdout—still better than chance, but a very different proposition. Top-decile enrichment falls from 6.82/7.09 (95 % CI 6.62–6.99 and 6.89–7.25) to 6.52/6.74 to 4.42/4.30 (3.79–5.06 and 3.67–4.92) across the same three protocols. The class-holdout interval is wide, and deliberately so: with 17 heterogeneous folds and k≈4 candidates in the top decile of the smallest, a point estimate would misrepresent what the data support.

Under high-$T_{g}$ extrapolation, the fixed-threshold decision is degenerate, since every test point is by construction above the training range, and only the ranking read-out is meaningful. Averaged over the four baselines, top-decile enrichment is 0.49 (0.32–0.71, stream A) and 0.83 (0.61–1.03, stream B). The two streams differ in what can be concluded. Stream A lies entirely below 1 across its bootstrap interval, so on this test set its ranking is worse than choosing candidates at random. Stream B’s interval spans 1, so it is not distinguishable from random. Neither four-baseline average provides evidence of reliable top-decile enrichment in this test. The strongest single model retains an enrichment of 1.55, against 7.75 on the random split. This is the sharpest statement the data support. The application in which a $T_{g}$ model is most valuable—finding repeat units hotter than anything already in the table—is exactly the regime in which a random-split $R^{2}$ of 0.90 carries no warning whatsoever. The histogram-gradient-boosting parity plot in Figure 3 shows boundary saturation for that model. It does not establish the same mechanism for ridge regression or neural networks; model-specific P4 results appear in Supplementary Table S11. The added unconstrained network on stream A gives mean R^2^ = −4.677 and MAE = 83.3 K over three initialisations, with mean enrichment 1.60 (seed range 1.01–2.09). It predicts some values above the training boundary, so its poor error cannot be attributed to a hard tree-like output bound. The archived stream-B ridge gives R^2^ = −4.695 and MAE = 84.5 K.

### 3.7. Reading the Errors Against the Between-Source Variability Reference

**Figure 8:**
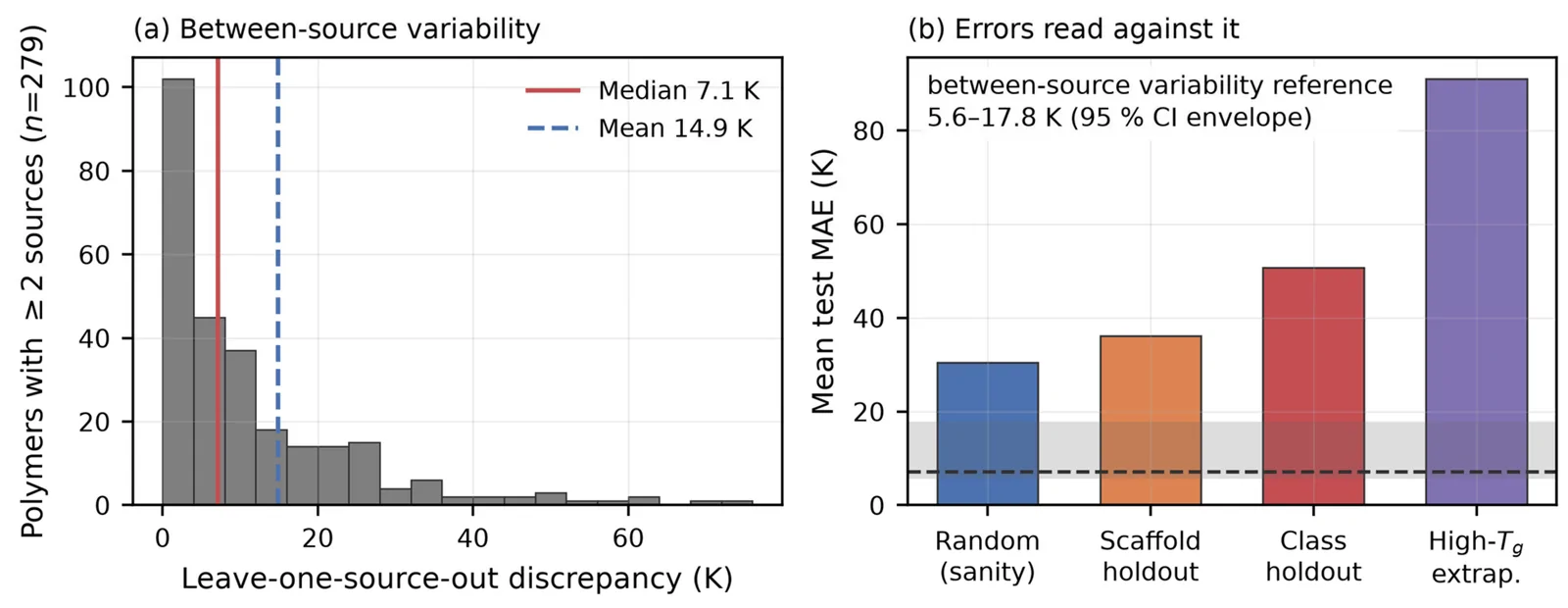
(**a**) Distribution of the leave-one-source-out discrepancy for the 279 polymers with at least two independent literature $T_{g}$ values; median *7.1* K, mean *14.9* K. (**b**) Mean test MAE by protocol, averaged over the four non-degenerate baselines and both streams, against the between-source variability reference (shaded band: the envelope of the 95 % bootstrap intervals of the median and the mean; dashed line: the median). The band is a variability scale, not an attainable error floor.

Of the 7367 records, 279 carry two or more independent literature values, and the curated table preserves the individual source values for all of them (132 have two sources, 91 have three, and the rest up to ten). Their leave-one-source-out discrepancy—the quantity that matches how the median label was built—has median 7.1 K (95 % CI 5.6–8.9), mean 14.9 K (12.3–17.8) and 90th percentile 33.8 K (Figure 8). The mean absolute pairwise difference between two reports is similar, median 7.0 K (5.9–8.9) and mean 15.5 K (12.9–18.6). The recomputed sample standard deviation has median 5.6 K and mean 12.1 K; correcting it for sample size raises it to 6.3 and 14.1 K.

The comparison reframes the protocol effect without licencing a claim about an attainable floor. A random-split MAE of 23.9 K is of the same order as the scale on which independent laboratories disagree about the same repeat unit—about 1.6 times the mean between-source discrepancy of 14.9 K and 3.4 times the median of 7.1 K—so further gains on this protocol are competing with the reproducibility of the labels themselves. A class-holdout MAE of 43.3 K, and still more a high-$T_{g}$ MAE of 79.5 K, are several times that scale and are therefore dominated by model error rather than by disagreement between sources. Effort spent improving random-split scores and effort spent improving chemistry-holdout scores are not competing for the same headroom.

What this quantity is not must be equally clear. It is not an attainable error floor and it bounds nothing. The model is trained and scored against the median over sources, not against a single laboratory report, so between-report dispersion is not the error an ideal model would make against the label actually used. It rests on 279 polymers, 3.8 % of the table, and those are by construction the most-studied ones, whose agreement need not represent the single-source majority. And it mixes measurement error with genuine specimen-to-specimen variation in molar mass, tacticity, thermal history and measurement method. That last conflation is informative rather than fatal—a model that sees only the repeat-unit structure cannot resolve any of those factors either—but it means the quantity is a variability scale, not a limit.

### 3.8. An Unsupervised Cluster Holdout on the Same Dataset

**Figure 9:**
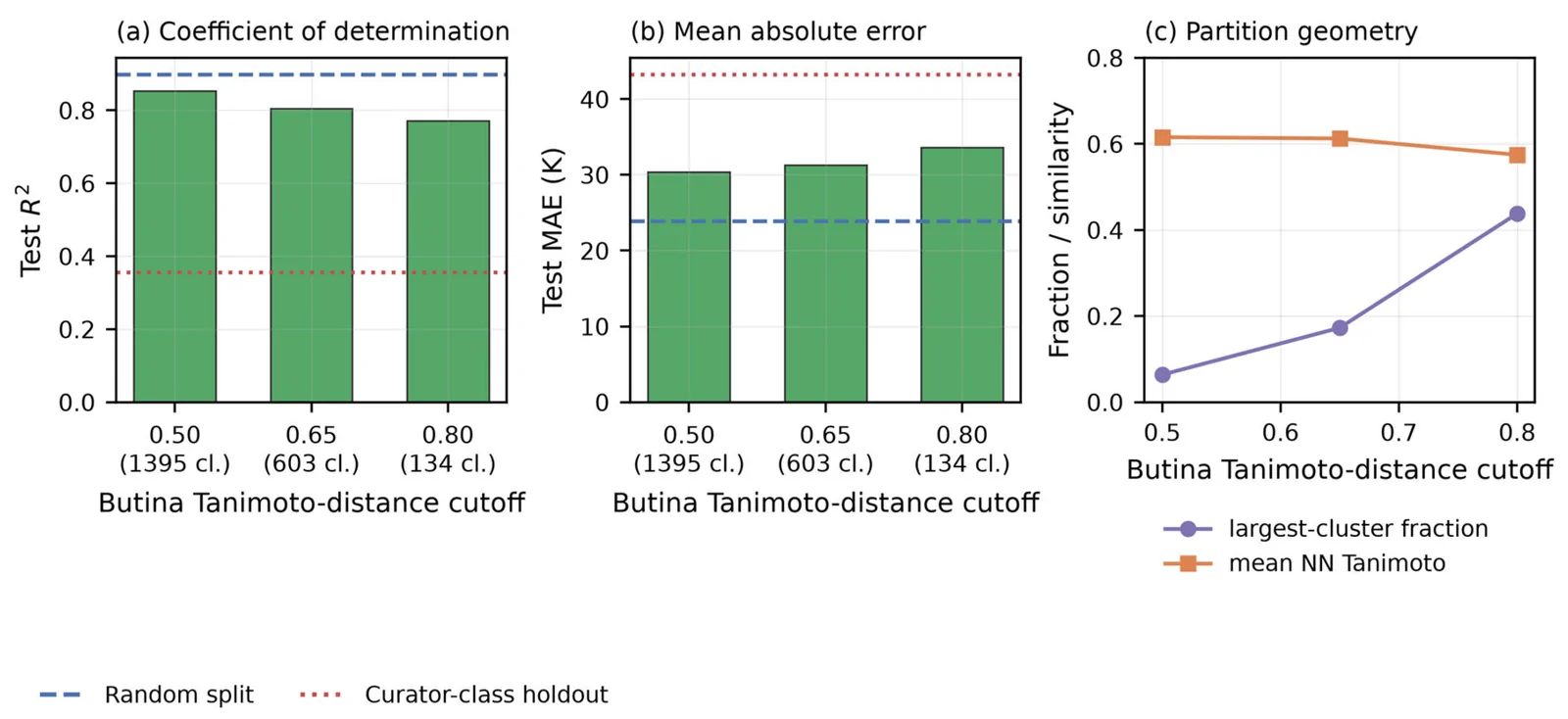
Unsupervised Butina cluster holdout on the $T_{g}$ dataset, histogram gradient boosting on stream B, and macro means over five whole-cluster folds. (**a**) Test $R^{2}$ and (**b**) test MAE at three Tanimoto-distance cutoffs, against the random-split (dashed) and curator-class-holdout (dotted) values of the same model. (**c**) Geometry of the partition: the fraction of the data in the largest cluster, and the mean nearest-neighbour Tanimoto similarity of a held-out repeat unit to its training partition.

The class-holdout protocol partitions on a curator-assigned name. To separate the effect of holding out a *region of structure space* from the effect of holding out a *named family*, the same $T_{g}$ data were also partitioned by Butina clustering of the 2048-bit Morgan fingerprints—the identical construction used for the external band-gap set—at three Tanimoto-distance cutoffs (Table 6, Figure 9).

The partition geometry behaves as expected: a cutoff of 0.50 gives 1395 clusters with the largest holding 6.4 % of the data, 0.65 gives 603 clusters (17.3 %), and 0.80 gives 134 clusters, with the largest holding 43.8 % of the data, which makes the five-fold construction at that cutoff poorly balanced and correspondingly poorly conditioned. Mean nearest-neighbour similarity falls only from 0.616 to 0.575 across the three, against 0.796 for a random split and 0.502 for the curator-class holdout.

The performance result is the informative one. For histogram gradient boosting on stream B, cluster holdout costs $R^{2}=0.899\to 0.854$, 0.805 and 0.772 and MAE 23.9→30.4, 31.3 and 33.7 K at the three cutoffs. At the cutoff of 0.65 used for the external set, $R^{2}$ falls to 0.805—close to the 0.888→0.832 drop that the same construction produces on the band-gap set, and nothing like the macro 0.355 of the curator-class holdout.

The matched comparison shows that holdout construction materially affects the apparent cross-dataset difference. It would be natural to attribute the milder structure-holdout penalty on the band-gap set to a property of that data—an enumerated, evenly sampled design space—but once the construction is held fixed the two datasets agree closely: fingerprint clusters and curator classes are not interchangeable partitions of the same data, and applying fingerprint clusters to Tg reproduces the band gap’s mild penalty on the very dataset whose named-class holdout collapses. Butina clusters are similarity groups; they split homologous series but they do not isolate a distinct bonding physics the way polyphosphazenes or polysiloxanes do, and several such clusters can therefore land on both sides of the split. Both dataset and partition can affect difficulty, so the construction must be stated before comparing datasets. For deployment into a named family with little representation in training, P3 is the more directly interpretable stress test if the class definition matches the candidate chemistry. For a diverse library without reliable family labels, Butina holdout offers a reproducible structure-based test; the fingerprint, cutoff and cluster-size imbalance must be reported. Neither substitutes for external validation on the intended library.

### 3.9. Where a Group-Contribution Prior Helps, and Where It Does Not

The fitted additive prior of Equation (1) is a competent but coarse predictor: with increments fitted in-fold it reaches $R^{2}=0.670$ and MAE 48.1 K on random held-out data, and $R^{2}=-0.047$ with MAE 57.0 K averaged over the 17 held-out classes. Twenty-six of the 35 group increments are non-zero in the representative full-data fit after the non-negativity constraint, and 24 of those 26 remain non-zero in every one of the 22 outer folds of the prior analysis; the largest belong to sulfone, amide and halogen groups, consistent with the stiffening and cohesive-interaction motifs that group-contribution schemes are built around [1]. Injecting a one-σ bias degrades the prior to $R^{2}=-0.34$ (random) and −3.02 (class holdout). The prior is thus a poor predictor on an unseen class; what follows asks whether it is nonetheless a useful constraint. In the targeted dictionary audit, removing the Si and P elemental groups preserves the larger additive-bias sensitivity of L1 in both inorganic families (Supplementary Table S7). For polyphosphazenes, raising δ from 0 to 1 changes R^2^ by −2.144 for L1 and −0.265 for L3 without those groups, compared with −2.354 and −1.101 with the full dictionary. For polysiloxanes the corresponding changes are −1.787 and −0.058 without the groups, and −1.745 and −0.014 with them. Absolute performance nevertheless depends on dictionary choice. This two-family stress test does not eliminate dataset-informed feature design or establish robustness to other kinds of prior error.

**Figure 10:**
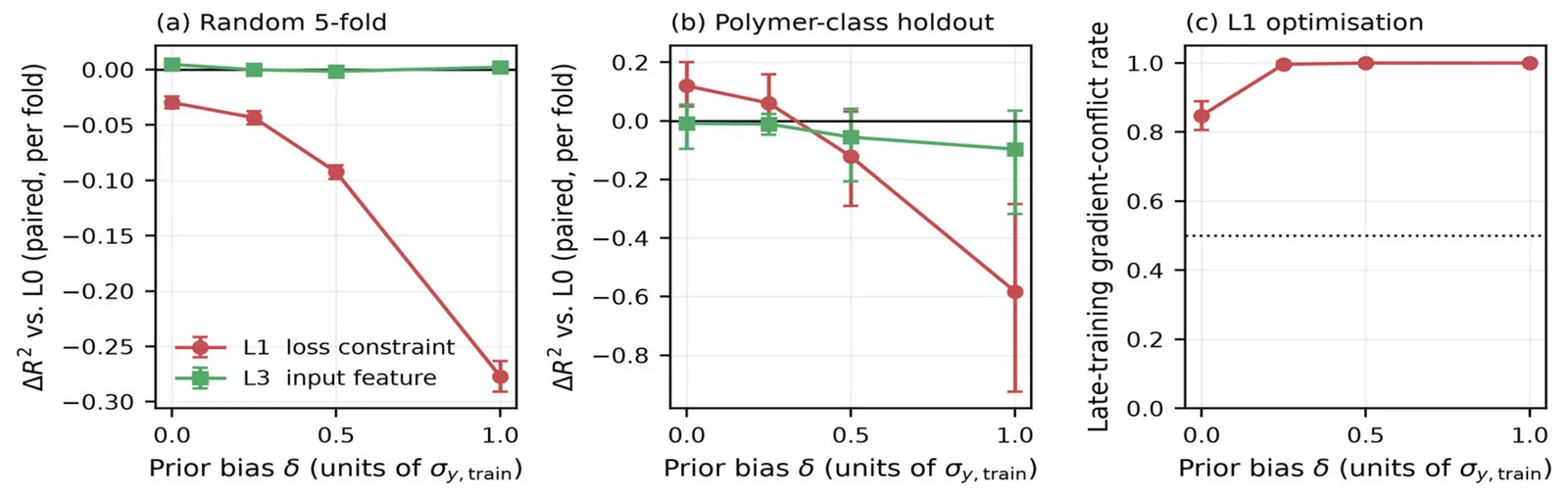
Integration of the group-contribution prior. (**a**,**b**) Paired change in test $R^{2}$ against the L0 baseline as a function of injected calibration bias, under random five-fold cross-validation and under polymer-class holdout. The statistical unit is the fold: the three seeds of each fold are averaged before the paired difference is taken, and error bars are percentile bootstrap 95 % confidence intervals over folds (5 under random splitting, 17 under class holdout; 5000 resamples), not standard errors over runs. (**c**) Fraction of late-training minibatch steps in which the data and prior gradients point in opposing directions under L1; error bars are standard deviations over runs. No random-split contrast can attain nominal p<0.05 with five folds; two class-holdout contrasts have nominal Holm-adjusted p<0.05 (Section 2.9, Table 7).

Table 7 and Figure 10 report how much this prior helps or harms depending on where it is inserted. Every entry is a paired difference against the L0 run on the same fold, with seeds averaged first, so n=5 per cell under random splitting and n=17 under class holdout. The two protocols therefore carry different evidential weight, as Section 2.9 sets out: the random-split family is floored at p=0.0625 and nothing in it can be significant, whereas the class-holdout family can be tested and two of its eight contrasts have nominal Holm-adjusted p<0.05.

**At** λ=1 **the loss constraint is harmful under random splitting at every tested calibration-bias level.** Even with in-fold calibration, L1 costs $\Delta R^{2}=-0.0297$ (95 % CI −0.0351 to −0.0244), and the penalty grows monotonically to −0.0434, −0.0925 and −0.2772 as the injected bias increases; the sign is negative in 5 of 5 random-split folds throughout. At the weaker weights λ=0.1 and 0.3 the calibrated constraint is approximately neutral instead (+0.005 and +0.001), so the harm is a statement about the penalty weight and not about loss-level integration in general (Table 8). The reading we offer is that the prior is simply less accurate than what the network learns from the descriptors, so forcing agreement with it displaces the solution.

**Under class holdout a well-calibrated constraint helps, and the window is narrower than it first appeared.** With in-fold increments L1 gains $\Delta R^{2}=+0.1196$ (+0.0506 to +0.1976), positive in 14 of 17 classes, and this is the positive contrast with the smallest nominal Holm-adjusted p ($p_{Holm}=0.033$). The gain is largely gone at a quarter-σ bias (+0.0607, −0.0333 to +0.1560, 11/17 classes), is already *harmful* at half a $\sigma_{y,train}$ (−0.1216, −0.2906 to +0.0296, 6/17 classes), and collapses to −0.5828 (−0.9284 to −0.2820, 1/17 classes) at one $\sigma_{y,train}$, which also reaches a nominal Holm-adjusted p of 0.003. The mechanism we suggest is ordinary regularisation: extrapolating to an unseen class, the low-variance additive prior is a useful anchor even though it is, in absolute terms, a worse predictor than the unconstrained network ($R^{2}=-0.047$ against 0.211).

Across all 17 held-out classes the calibrated constraint therefore shows a sizeable positive mean effect, while half- and one-$\sigma_{y,train}$ miscalibration reverse it quickly. The usable window is narrow in a specific and practical sense: at λ=1 it closes below half a training-fold standard deviation of calibration error, whereas the weaker penalties λ=0.1 and 0.3 still retain a small positive effect (+0.035 and +0.033) at that same level of bias. The quantity that decides it is exactly the one hardest to verify before deployment.

**Supplying the prior as a feature is substantially less sensitive to calibration bias, though not insensitive to it.** Under random splitting L3 is marginally positive at zero bias (+0.0047, +0.0027 to +0.0068, 5/5 folds) and numerically small throughout the rest of the sweep (−0.0001, −0.0015, +0.0022). The descriptive intervals straddle zero at δ=0.25 and 0.5; at δ=1 the interval is narrowly positive (+0.0004 to +0.0042), though with five folds the sign-flip design cannot attain nominal p<0.05 in any case. Under class holdout it ranges from −0.0966 to −0.0088; every interval straddles zero and no cell has a nominal Holm-adjusted p<0.05 (all $p_{Holm}=1.00$), but the point estimate at bias of one $\sigma_{y,train}$ is not negligible. The contrast with L1 is one of degree, and the degree is large: as the injected bias grows from 0 to $1\sigma_{y,train}$, L1’s effect moves by 0.248 (random) and 0.702 (class holdout), while L3’s moves by 0.003 and 0.088. This asymmetry is compatible with the network parameterisation and the target-shrinkage form of L1; it does not establish a general superiority of feature-level integration.

The recorded late-training gradient conflict is descriptive. At zero injected bias the negative-cosine fraction is 79.3% under random splitting and 86.4% under class holdout, increasing when bias is added. However, Equation (2) is equivalent to a scaled squared error against the blended target (y + λg)/(1 + λ). Opposing gradients indicate disagreement between the empirical targets, rather than evidence for competition between an experimental objective and an independent physical law. Whether shrinkage improves held-out prediction is decided by paired errors, not the conflict rate alone.

### 3.10. The Penalty Weight Decides How Much Miscalibration Can Be Tolerated

**Figure 11:**
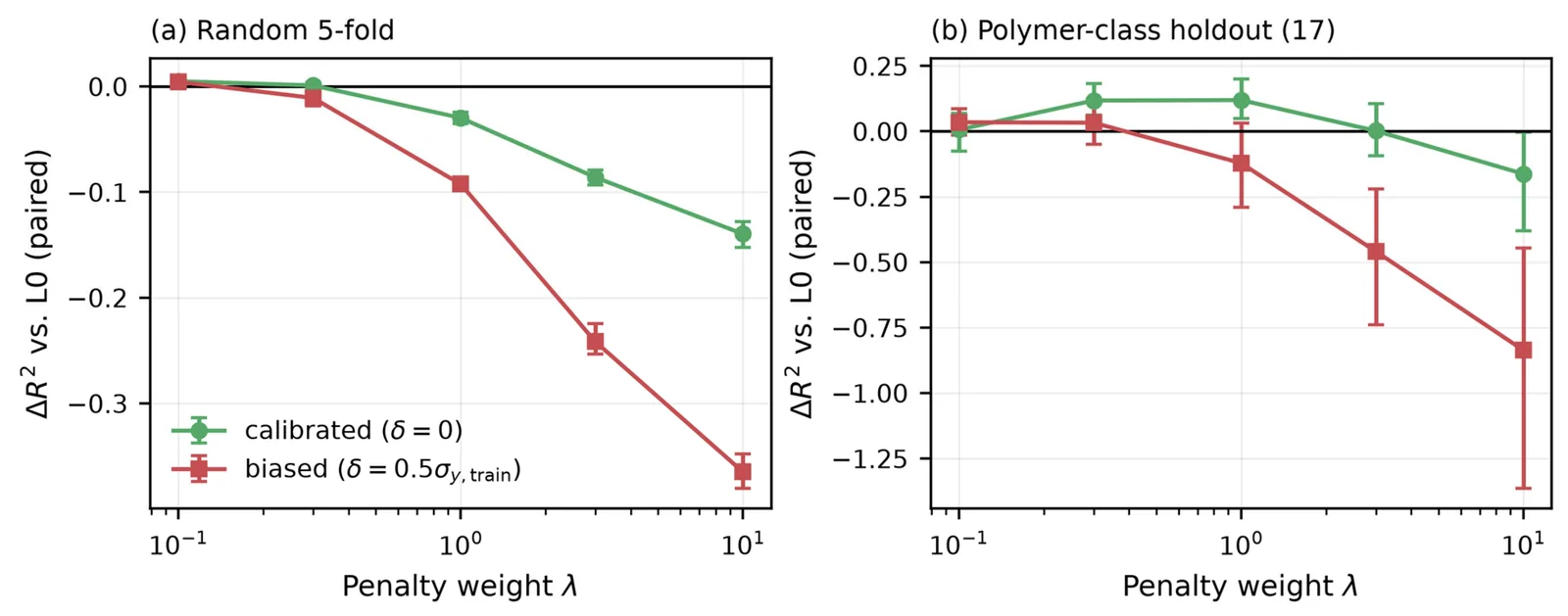
Paired $\Delta R^{2}$ against the L0 baseline as a function of the penalty weight λ, for a calibrated prior and one biased by half a standard deviation, under random five-fold cross-validation and polymer-class holdout. Under random splitting the constraint can only do harm once it is strong enough to matter; under class holdout there is an interior optimum. Both λ=0.1 and λ=0.3 retain a small positive effect under half-$\sigma_{y,train}$ miscalibration (*+0.035* and *+0.033*); λ=0.3 is the largest tested weight that does so while still retaining a substantial benefit when the prior is calibrated. Points are means over folds—5 under random splitting, 17 under class holdout—with seeds averaged within a fold.

The factorial above fixed λ=1. Sweeping λ over two decades (Figure 11, Table 8) shows that the qualitative pattern is robust but that the operating point matters more than the choice of integration mode alone. As above, the unit is the fold: five under random splitting, 17 under class holdout.

Under random splitting the damage done by L1 is monotone in λ: with a calibrated prior, $\Delta R^{2}$ runs +0.005, +0.001, −0.030, −0.086, −0.140 for λ=0.1,0.3,1,3,10. A very weak constraint is harmless because it barely moves the optimum; anything strong enough to matter is harmful, because the prior is less accurate than what the model learns from the descriptors.

Under class holdout the benefit is an interior optimum. With a calibrated prior, $\Delta R^{2}$ rises from +0.006 at λ=0.1 to +0.118 at λ=0.3—positive in 15 of 17 classes—is flat at +0.120 for λ=1, and then falls to +0.002 and −0.163 at λ=3 and 10.

The practically important comparison is what happens when the prior is miscalibrated by half a $\sigma_{y,train}$, and this is where the choice of λ stops being cosmetic. At λ=1 the gain does not merely disappear; it inverts: −0.122, positive in only 6 of 17 classes. At λ=0.3 a smaller part of it survives (+0.033, positive in 10 of 17), and at λ=0.1 +0.035. At λ=3 and 10 a half-$\sigma_{y,train}$ error costs −0.459 and −0.837. Among the tested values, λ=0.3 therefore gave the most favourable robustness trade-off on this dataset—not because it is best when the prior is right, where it ties with λ=1, but because it is the largest weight that still degrades gracefully when the prior is wrong. This is a retrospective sensitivity result on the outer class-holdout folds, not a prospectively validated optimum: no independent dataset was used to confirm that the same weight is preferable elsewhere. λ≳3 was harmful in every condition tested.

### 3.11. External Replication on a Second Open Polymer Property

To test whether the protocol effect is specific to $T_{g}$, or to experimental data, the same machinery was applied to a second openly accessible set: the DFT-computed chain band gap of 4127 polymers distributed in the polyVERSE repository [32]. This property is computed under a common workflow rather than measured in different laboratories, so it carries no inter-laboratory experimental scatter, though it carries computational error of its own (Section 4.5). The dataset has no curator-assigned classes, so chemistry families were defined by Butina clustering of the Morgan fingerprints at a Tanimoto-distance cutoff of 0.65, giving 573 clusters. Whole clusters were then assigned to five size-balanced folds and held out groupwise, giving a cluster-grouped five-fold holdout: a fold contains many complete clusters rather than one, so this is analogous in spirit to the leave-one-cluster-out validation of Meredig et al. [23] rather than identical to it.

**Figure 12:**
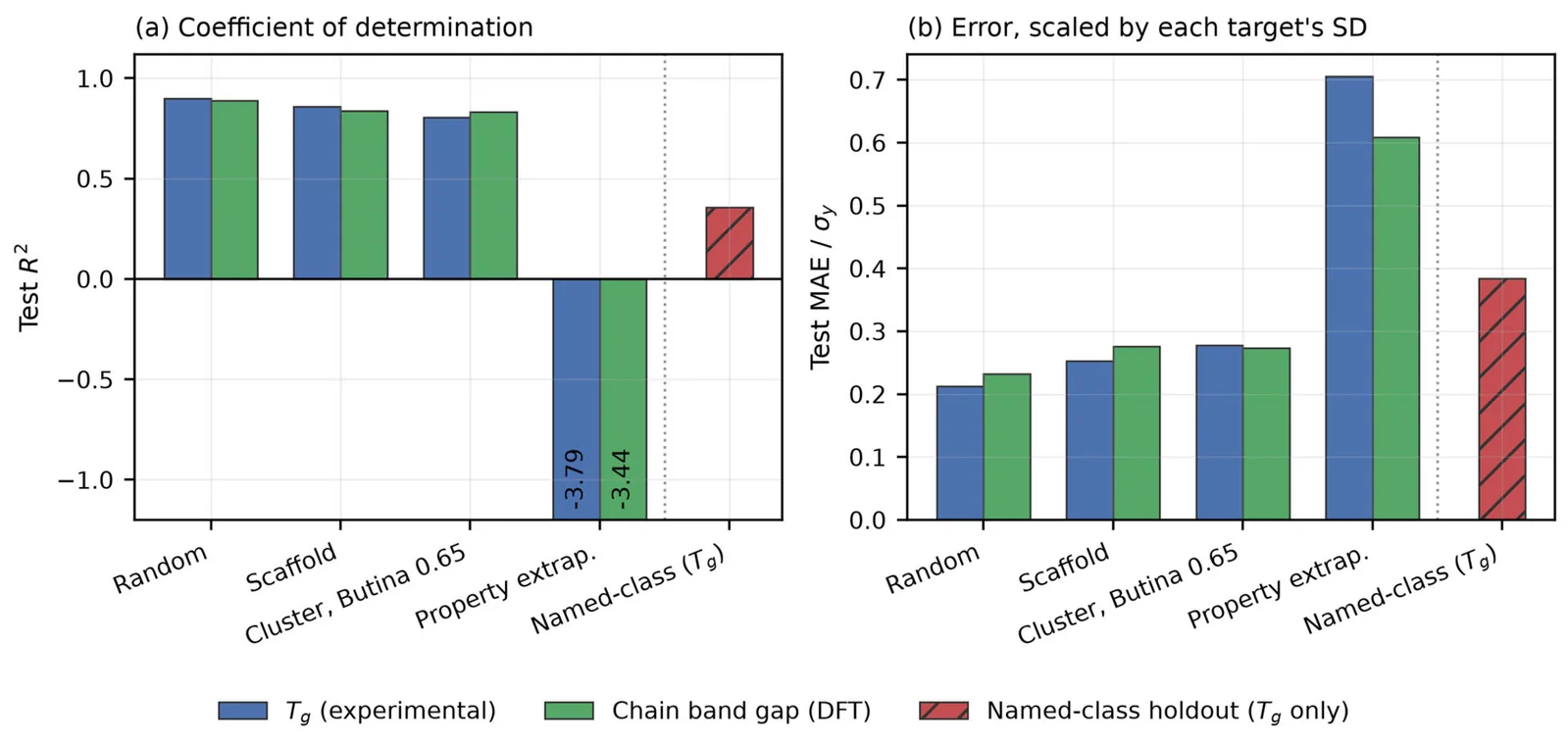
External replication, with the holdout construction matched. Histogram gradient boosting on the experimental $T_{g}$ set (stream B) and on the DFT-computed chain band gap. The four protocols left of the dotted rule use the same construction on both datasets, including fingerprint-cluster holdout by Butina clustering at a Tanimoto-distance cutoff of 0.65 for each. Under that matching, the two datasets agree closely: $R^{2}$ 0.805 against 0.832 and normalised MAE 0.277 against 0.273 for cluster holdout. The bar right of the rule is the $T_{g}$ curator-named-class holdout, which has no band-gap counterpart because that set carries no curator classes; it is drawn separately and hatched so that it is not read as a dataset effect. (**a**) $R^{2}$, clipped at −1.2 with the property-extrapolation values annotated. (**b**) MAE normalised by each target’s standard deviation (112.7 K and 1.44 eV), which is what makes the two targets comparable. Source data: figure12_like_for_like.csv. All bars refer to a single model; Table 9 reports four-model averages. Supplementary Table S6 reconciles the presentations.

The result (Figure 12, Table 9) separates cleanly into a part that replicates and a part that does not, and it is worth being precise about which is which.

**Property-range extrapolation replicates almost quantitatively.** Holding out the top quintile of the band gap gives $R^{2}=-3.44$ and a top-decile enrichment of 1.56 for the strongest model, against −3.79 and 1.55 for $T_{g}$; averaged over the baselines, enrichment is 0.54 against 0.49–0.83. Because the band gap labels carry no inter-laboratory scatter, this failure cannot be attributed to experimental disagreement between sources. It is a property of asking a model to rank candidates outside the range it was fitted on.

The structure-holdout comparison depends on model aggregation. For histogram gradient boosting alone, band-gap R^2^ changes from 0.888 to 0.832 and MAE from 0.334 to 0.393 eV between random and Butina-cluster holdout. These are the single-model results in Figure 12, not the four-model mean in Table 9. The latter changes from R^2^ = 0.791 to 0.673 and MAE = 0.431 to 0.534 eV. The matched single-model Tg comparison is R^2^ = 0.899 to 0.805. For cluster holdout, normalised MAE is 0.277 (Tg) and 0.273 (band gap) for histogram gradient boosting, but 0.355 and 0.371 for the four-model means. Supplementary Table S6 shows both aggregations. Both show a fingerprint-cluster penalty; neither licences equating that construction with curator-class holdout.

The cross-dataset conclusion is limited: the tested baselines show poor property-range extrapolation on both targets, and a moderate penalty for fingerprint-cluster holdout. The much lower Tg class-macro R^2^ is a result for the curator-defined P3 partition, whose folds differ in size and chemical novelty. Polyphosphazene and polysiloxane biases identify particularly difficult inorganic-backbone families, but do not prove a general failure on every unseen polymer chemistry.

### 3.12. How Many Measurements Does a New Polymer Class Need, and for What?

**Figure 13:**
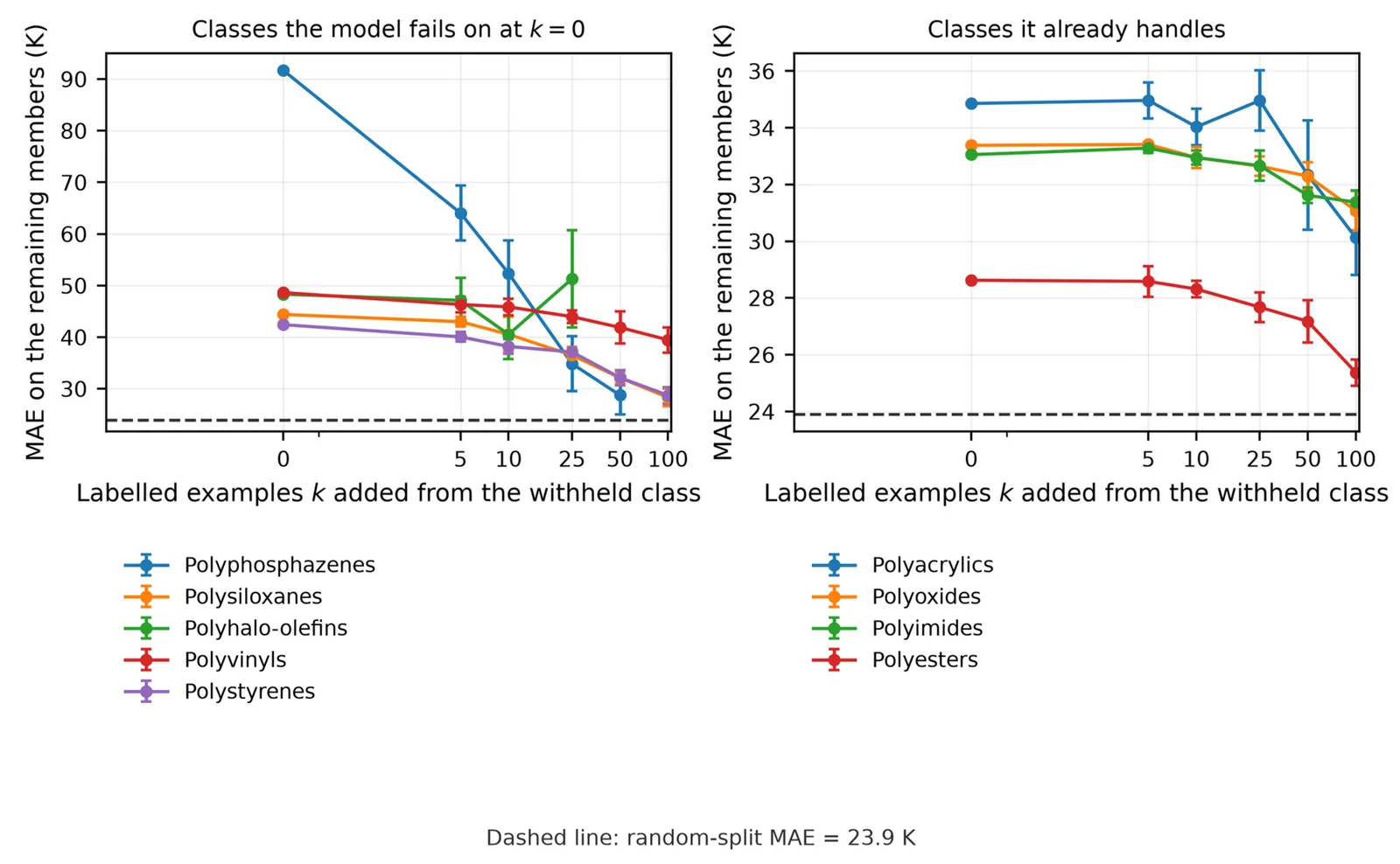
Few-shot calibration of a withheld polymer class by retraining. (**Left**) The classes on which the zero-shot model fails. (**Right**) Classes it already handles. Model is histogram gradient boosting on stream B. Error bars are standard deviations over five random draws of the calibration subset; the dashed line is the random-split MAE of the same model.

The class-holdout result states a problem; the natural follow-up is what it costs to fix. Adding k labelled members of the withheld class back into the training set and scoring on the remainder converts the finding into an experimental budget (Figure 13, Table 10).

The recovery is dramatic precisely where the failure was a systematic offset. For polyphosphazenes, MAE falls 91.7→64.0→52.4→34.8→28.7 K for k=0,5,10,25,50: 25 measured repeat units reduce MAE by approximately 62%, and 50 bring the class within 5 K of the random-split reference of 23.9 K while still leaving 57 of its 107 members as test data. The k=100 cell is not reported for this class: it would leave seven test points, 6.5 % of the class, below the one-third threshold required by the protocol (fewshot_reporting_audit.csv). Polysiloxanes behave the same way (44.4→28.3 K, bias +33.9→+8.3 K), as do polystyrenes (42.4→28.7 K, bias −17.2→−6.2 K). Where the error was diffuse rather than biased, few-shot calibration buys much less: polyvinyls improve only from 48.6 to 39.5 K, and the classes the model already handled—polyesters, polyimides, polyoxides, polyacrylics—move by about 2–5 K over the whole sweep, because there was no class-level offset to remove.

**Figure 14:**
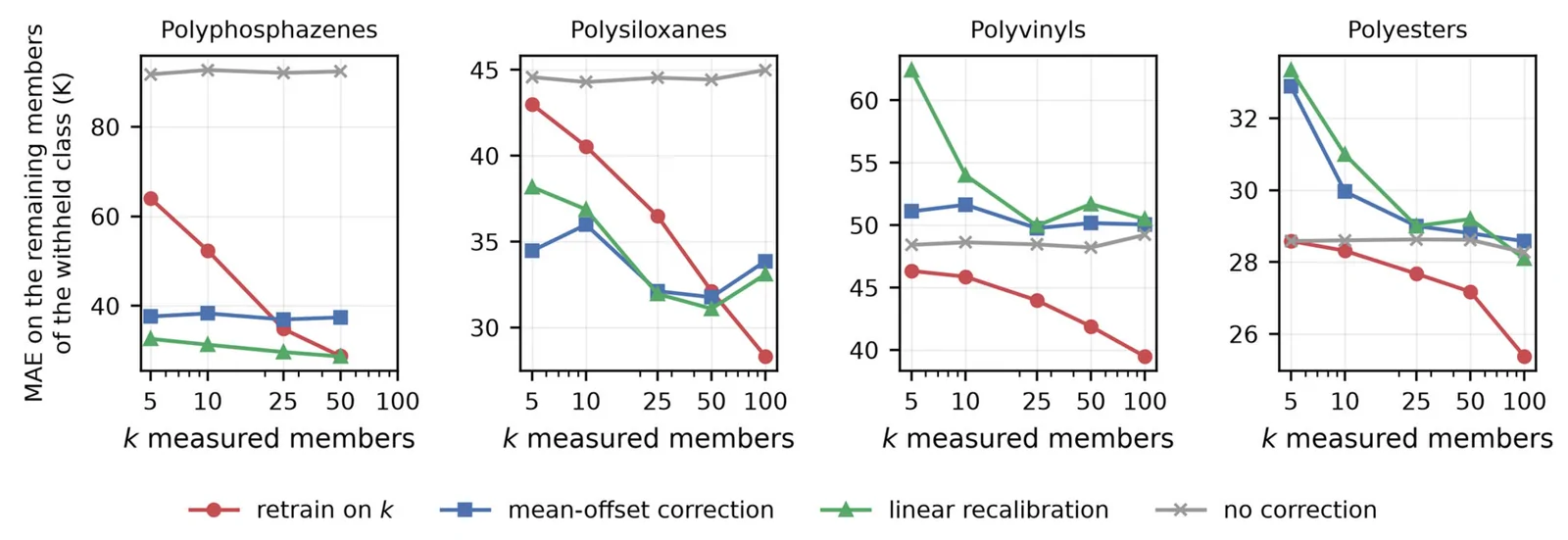
What the k labels buy: retraining compared with two cheaper baselines that consume exactly the same k labelled members of the withheld class, plus the uncorrected zero-shot model. Means over five random draws; note that each panel has its own vertical scale, because the four classes differ by a factor of three in error. Where the zero-shot failure is a class-level offset (polyphosphazenes, polysiloxanes) a one- or two-parameter correction beats retraining until k is large; where it is diffuse (polyvinyls, polyesters) both corrections are worse than doing nothing.

**A retraining-only comparison is, however, incomplete.** Comparing retraining with a mean-offset correction and a linear recalibration that consume exactly the same k labels shows that most of the early gain in the polyphosphazene recovery is intercept correction, not re-learned chemistry (Table 11, Figure 14). With only five labelled polyphosphazenes, a constant offset takes MAE from 91.7 to 37.6 K and a linear recalibration to 32.6 K, while retraining on the same five members reaches only 64.0 K. Retraining needs ten times as many labels to catch up: at k=50 it reaches 28.7 K, the level already achieved by linear recalibration, while the constant offset saturates at 37.4 K because it can only remove an intercept. On this class, a two-parameter recalibration is a better use of the smallest tested label budgets, and retraining becomes competitive only once k approaches half the class. The same ordering holds for polysiloxanes at small k (34.5 and 38.2 K for offset and linear against 43.0 K for retraining at k=5).

The corrections are not free, and where the error is diffuse they are actively harmful. For polyesters, whose zero-shot bias is only −3.4 K, a mean-offset correction fitted on five members raises MAE from 28.6 to 32.9  K and a linear recalibration to 33.3 K, because a five-point estimate of an almost-zero offset is mostly noise. Polyvinyls, with a −3.2 K bias but a large scatter, behave the same way (48.4→51.1 K under offset correction at k=5). A useful decomposition is prediction error = bias + centred residual. For representative calibration draws, the variance of an estimated mean bias scales approximately as the residual variance divided by k. Removing a large common offset can help immediately, whereas correcting a near-zero mean from a small dispersed sample adds estimation noise. An offset cannot learn structure-dependent residuals or change rankings; retraining can change the mapping, but a few target-class points are diluted among thousands of existing examples. This explains the observed comparison without guaranteeing the same ordering for every class. All four treatments use identical calibration indices and the same remaining test set for each class, k and draw.

This gives a usable diagnostic, with one caveat about how it is obtained. The signed bias of a held-out class can only be computed once some of that class is labelled, so it is free in retrospective evaluation but not in deployment, where the class is by definition unmeasured. The deployable form is to estimate the class-level bias from the same small labelled calibration set that the remedy would use, and let that estimate inform the remedy: a substantial systematic offset is cheap to correct with a one-parameter shift or a linear recalibration, whereas diffuse residual error needs many more labels and a retrained model, or a decision not to score that class at all. The results illustrate different error patterns; they do not validate a prospective rule for choosing the correction from only five measurements. Such a rule requires independent evaluation. Polyhalo-olefins illustrate the limits of the measurement rather than of the method: with only 40 members, k=25 leaves 15 test points and the curve becomes non-monotone, so small classes cannot be characterised this way.

### 3.13. Abstention: What Survives Prospective Threshold Selection

Ranking candidates by their maximum Tanimoto similarity to the training set and declining to score the distant ones is the natural operational use of Section 3.4. Whether it works depends entirely on how the threshold is chosen, and a retrospective choice flatters it.

**Figure 15:**
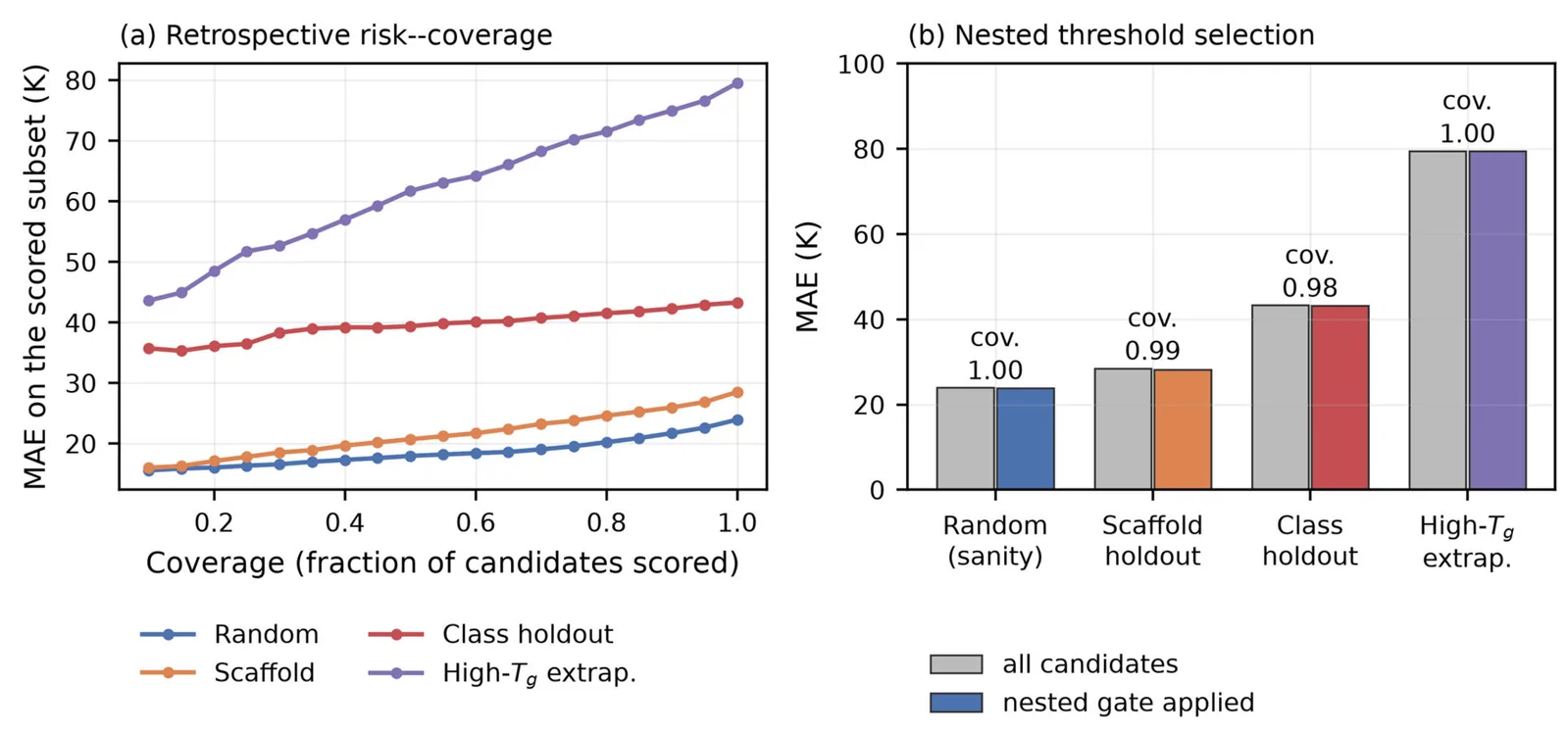
Abstention on structural distance, histogram gradient boosting on stream B. (**a**) Retrospective risk–coverage curves: MAE on the scored subset when candidates are ranked by maximum Tanimoto similarity to the training partition and only the closest fraction is scored, with the coverage chosen on the observed holdout. Curves are macro means over the folds of each protocol. This is descriptive, not a deployable gate. (**b**) The same gate with the threshold selected inside the outer training partition and then frozen: grey bars are the full-fold MAE, coloured bars the selective MAE, and the annotation is the realised outer coverage. The gate almost never fires and buys almost nothing.

**Retrospectively, the gate looks useful, but it is not.** Sweeping coverage on the observed holdout (Figure 15a) shows MAE falling from 43.3 K at full coverage to 36.1 K at 20 % coverage under class holdout, where the implied similarity threshold averages 0.62; under high-$T_{g}$ extrapolation, it falls from 79.5 to 48.5 K. These curves are descriptive. They quantify how error is distributed across the similarity axis, and they are computed with knowledge of the very errors the gate is supposed to avoid.

A pooled version of the same retrospective analysis overstates the benefit, giving 36.6→25.5 K at 20 % coverage. Pooling class-holdout predictions across the 17 folds both changes the baseline from the macro 43.3 K to the pooled 36.6 K and lets the gate preferentially discard whole difficult classes, so the macro curve above is the appropriate one.

**Prospectively, on this dataset, the gate barely fires.** When the threshold is selected inside the outer training partition on a simulated holdout and then frozen (Section 2.11), the selected thresholds are low—0.24 on average for class holdout, 0.20 to 0.27 for the other protocols—and outer coverage averages 0.98 to 1.00 by protocol, falling no lower than 0.86 in any single fold. Selective MAE is then indistinguishable from full-fold MAE: 43.21 against 43.29 K under class holdout, 28.13 against 28.53 K under scaffold holdout, 23.89 against 23.95 K under random splitting, and exactly 79.52 K under high-$T_{g}$ extrapolation, where nothing is abstained on at all (Figure 15b). Prospectively, therefore, this gate does **not** restore near-random-split accuracy; it barely fires at all.

We think the reason is instructive rather than merely negative. The inner holdout is a scaffold-grouped quarter of the training partition, and scaffold groups are far finer than polymer classes, so it stays chemically close to the inner training set. Its similarity distribution is therefore much more favourable than the one produced by a genuinely withheld class, and the cut that meets a 30 K selective-MAE target there is too permissive to fire on the real holdout. A gate calibrated on chemistry the model already knows does not generalise to chemistry it does not. That is the same failure mode as the model itself, one level up, and it means that on this dataset the threshold could not be learned reliably from the standard inner holdouts; external information about the intended candidate library, or a deliberately more adversarial calibration design, may therefore be required. The retrospective curves remain useful for that purpose—they say what a correctly chosen threshold could achieve—but they are not evidence that the threshold can be chosen automatically.

### 3.14. Calibrated Intervals Fail Under Shift, and the Failure Is Conditional

Table 12 and Table 13 test whether reported uncertainty is reliable for unfamiliar polymers. They use a 300-tree random forest on stream B, whereas the single-model headline and parity plots use histogram gradient boosting. These coverage results are a model-specific stress test, not calibrated intervals for the headline booster.

**The tree-dispersion heuristic is not a prediction interval.** Its empirical coverage is 0.967, 0.962 and 0.931 under P1, P2 and P3—far above the 0.90 implied by the 1.645 multiplier, i.e., systematically conservative—and then collapses to 0.238 under high-$T_{g}$ extrapolation. The intervals are also *narrower* in the regime where the model is worst: the mean reported spread is 37.5 K under high-$T_{g}$ extrapolation against 66.0 K under class holdout, while the corresponding MAEs are 95.7 and 47.0 K. This is why the quantity is relabelled rather than merely recalibrated: a score that is conservative in three regimes, anti-conservative in the fourth, and anti-correlated with error across them is not measuring predictive uncertainty.

**Split conformal achieves near-nominal marginal coverage under random splitting and degrades under distribution shift.** Coverage is 0.899 under random splitting, consistent with the nominal 0.90 marginal coverage expected under exchangeability, and 0.851 under scaffold holdout, a mild violation. Under class holdout it falls to 0.683 and under high-$T_{g}$ extrapolation to 0.214. Conformal prediction guarantees marginal coverage only when calibration and test data are exchangeable, and holding out a whole polymer class or the top quintile of the target is precisely a violation of that assumption. The value of running it is that the size of the violation is now measured rather than assumed: a practitioner can read 0.683 and 0.214 as the price of the two kinds of shift. Intervals are also much narrower than the tree-dispersion ones (116–119 K against 149–217 K), so the tree heuristic bought its conservatism with uselessly wide intervals.

**Figure 16:**
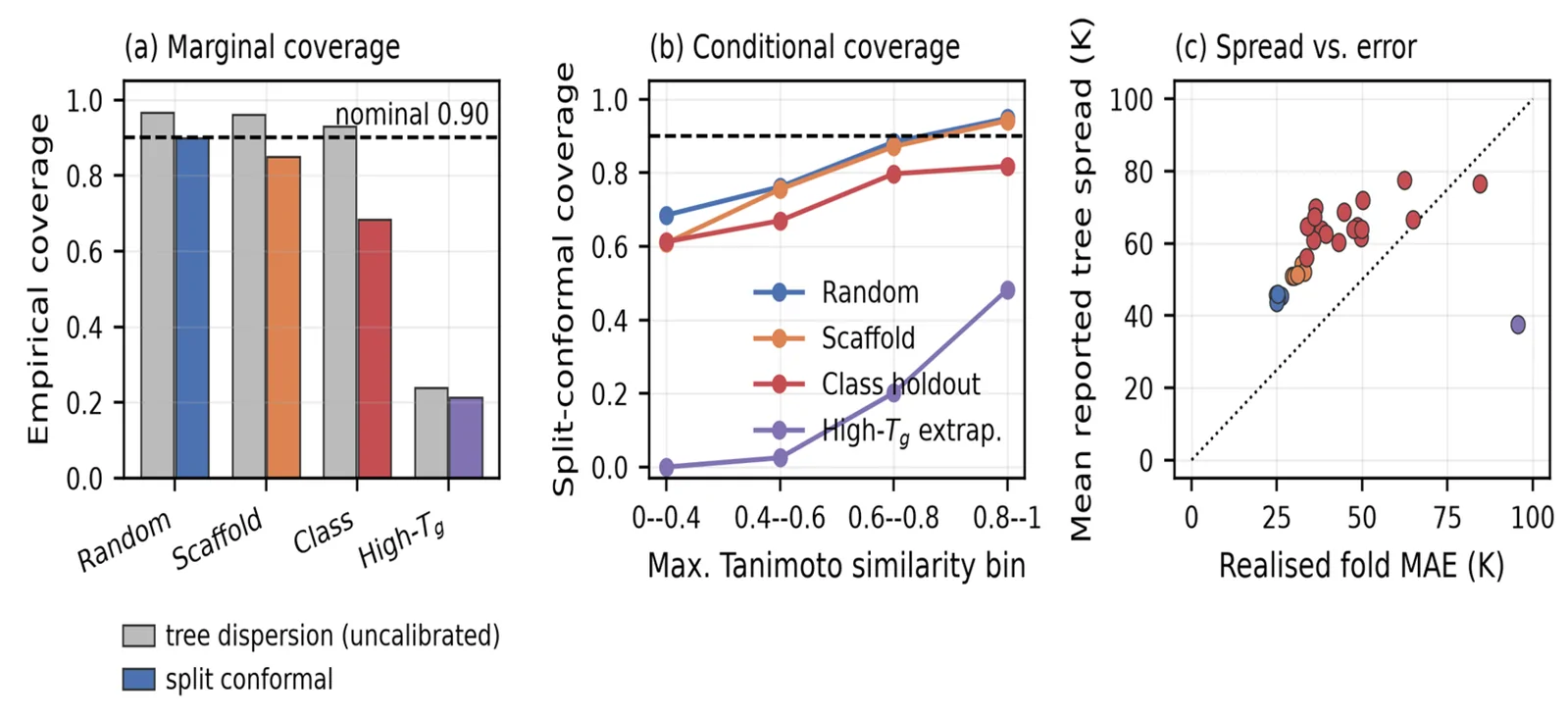
Interval calibration, 300-tree random forest on stream B, macro means over the folds of each protocol. (**a**) Marginal empirical coverage of the uncalibrated tree-dispersion heuristic (grey) and of split conformal intervals at α=0.10 (coloured); the dashed line is the nominal 0.90. (**b**) Split conformal coverage conditional on the test point’s own maximum Tanimoto similarity to its training partition: coverage degrades with structural distance even inside the random split, whose marginal coverage is near nominal. (**c**) Mean reported tree spread against realised fold MAE; the dotted line is parity. Points above it have mean spread exceeding fold MAE; the single point far below it is the high-$T_{g}$ extrapolation fold, where the model reports its narrowest spread and makes its largest errors. Panel (**b**) averages only cells with at least 10 polymers; Table 13 reports the contributing folds and sample totals.

**Conditional coverage fails inside the random split too.** Stratifying by the test point’s own maximum Tanimoto similarity (Table 13, Figure 16b) shows conformal coverage of 0.948 for the closest stratum (similarity ≥0.8) but 0.685 for the 29 test points per fold with similarity below 0.4—*within* the random-split protocol, where marginal coverage is near nominal. The same gradient appears under scaffold holdout (0.942→0.609) and class holdout (0.818→0.613). A marginally valid interval can therefore be badly miscalibrated on exactly the candidates a screening campaign cares about, and reporting marginal coverage alone conceals this.

Consistent with all of the above, the correlation between the reported tree spread and the realised absolute error is weak within class holdout (mean r=0.26) and essentially zero for the worst class, polyphosphazenes (r=0.08, tree coverage 0.841, conformal coverage 0.224). An ensemble spread computed on conventional organic-backbone training data cannot express ignorance about a backbone chemistry it has never seen. The practical implication is to validate coverage under the intended shift and report conditional as well as marginal coverage. Neither tested interval construction provides reliable protection in P3 or P4. Candidate-family measurements and an independently checked calibration design are needed before using intervals for deployment decisions.

## 4. Discussion

### 4.1. What a Reported $R^{2}$ Estimates

The four protocols do not disagree about the model; they answer different questions, and within the model family examined here the choice of protocol changes the reported score by more than the choice of model does. A random-split $R^{2}$ of 0.90 is a correct estimate of how well the model interpolates within a densely sampled chemical neighbourhood. The similarity audit makes the size of that neighbourhood explicit: a third of randomly held-out repeat units have a training analogue at Tanimoto ≥0.9. This is not a defect of the curated dataset; it is what a literature-derived polymer table looks like, because experimental $T_{g}$ studies are published as homologous series. The consequence is that random splitting on such a table primarily measures within-series interpolation performance. For the deployment question—ranking an unfamiliar chemistry—that is the wrong figure of merit, in exactly the sense identified for prospective prediction in cheminformatics [19,21] and for materials discovery [23,24].

The practical size of the gap is what the decision read-outs quantify. It is tempting to treat a fall in $R^{2}$ from 0.90 to 0.36 as a moderate loss of precision. Top-decile enrichment says otherwise. For the strongest model it falls from 7.7 under random splitting to 5.3 under class holdout and to 1.6 under high-$T_{g}$ extrapolation; averaged over the four transparent baselines, the last figure is 0.5–0.8, with bootstrap intervals that include or lie below chance. A screening campaign guided by the random-split score would expect a sevenfold enrichment and, if it were searching above the known $T_{g}$ range, obtain something not distinguishable from none.

### 4.2. Three Tiers of Conclusion

Because the two datasets do not agree in every respect, it is worth separating what this study establishes from what it merely suggests.

**Supported directly, within this** $T_{g}$ **dataset.** A random split measures interpolation inside a densely sampled chemical neighbourhood, and its $R^{2}$ is not an estimate of extrapolative performance. Holding out a curator-named polymer class is markedly harder than holding out scaffolds or fingerprint clusters, and the per-class failures are systematically biased in chemically interpretable directions, with polyphosphazenes over-predicted by +91 K. Structural proximity has a reproducible but limited association with error at sample level, with protocol controlled (≈−49 K per unit Tanimoto similarity, stable across six fingerprint variants), while explaining only 6–11 % of its variance. Neither nested tuning on fused features nor additional training data from already represented chemistries closes the gap; a few labelled members of the withheld family itself do help (Section 3.12). Marginally valid conformal intervals become badly miscalibrated on structurally distant candidates even within the random split.

**Supported by both datasets.** Property-range extrapolation fails catastrophically on an experimental target and on a computed one, at $R^{2}=-3.79$ and −3.44 with top-decile enrichment of 1.55 and 1.56. The penalty for holding out fingerprint clusters is consistent in direction and moderate in size on both (0.899→0.805 for $T_{g}$, 0.888→0.832 for the band gap) once the partition construction is held fixed. The quoted R^2^ pairs and enrichment values are for histogram gradient boosting alone; Table 9 gives four-baseline averages. These findings do not establish failure of all extrapolation-capable models.

**Not established by this work.** That every polymer property collapses the same way under chemistry holdout—two properties are not a survey. That graph neural networks or chemical language models pretrained on large unlabelled corpora [8] would fail in the same way; they might transfer across chemistries in a way fixed descriptors plus trees cannot, and testing that is the obvious next experiment. That all uncertainty methods fail: we tested two, and split conformal is marginally valid where its assumptions hold. That feature-level integration of prior knowledge is generally superior to loss-level integration; our evidence covers one prior, one compact architecture, one additive bias model and one dataset, under a design that does not support exact repeated-sampling inference.

### 4.3. Practical Recommendations for Evaluation and Deployment

Most of the recommendations below require no new model, no new descriptor and no new experimental data: they are reporting conventions. The exception is remediation of a genuinely unseen class. Where a class-specific bias has to be corrected rather than merely detected, the few-shot results of Section 3.12 show that a small labelled calibration set drawn from that class is the intervention that helps, and that is a real experimental cost. The conventions first:

1. Report a chemistry-holdout score *alongside* the random-split score, never instead of it, and say how the holdout was constructed. The gap between them is itself the useful quantity; a small gap is evidence that the model has learned transferable structure–property relations rather than series membership. Section 3.8 shows that fingerprint-cluster and curator-class holdouts on the same data differ by 0.45 in $R^{2}$, so “chemistry holdout” without a construction is not a specification.
2. State the applicability domain quantitatively. The distribution of nearest-neighbour Tanimoto similarity between the intended candidates and the training set costs one matrix product, and absolute error falls by about 49 K per unit similarity with the protocol controlled.
3. Report per-holdout results, not only their pooled aggregate. Pooling over 17 held-out classes turned a macro $R^{2}$ of 0.36 into a pooled 0.80 purely through the between-class variance in the denominator.
4. Estimate class-level bias from a small labelled calibration set, and let that estimate inform the remedy. The signed bias of a genuinely unseen class cannot be known before any of it is measured, so the retrospective biases in Table 5 explain the failure mechanism but are not themselves a deployment tool. What is deployable is the same handful of labels the remedy would need anyway: a substantial systematic offset favours a low-cost mean-offset or linear recalibration—five measured polyphosphazenes and a one-parameter offset removed more error than retraining on the same five—whereas diffuse residual error requires more labelled examples, retraining, or declining to score the class, and applying an offset correction there made polyesters and polyvinyls worse.
5. Report interval coverage conditionally, not marginally. Split conformal is near-nominal under a random split and still covers only 0.685 of its least-similar stratum (similarity below 0.4), which is 2.0 % of that same random split’s test points.
6. Do not answer a chemistry-holdout gap simply with a bigger model or with more training data from chemistries that are already represented. Section 3.3 shows that tuning and feature fusion bought 7 % on the random split and nothing on the class holdout, and that class-holdout error stops falling beyond half the training pool while the random split is still improving. For one of the 17 held-out classes, the most out-of-domain family, additional in-domain training data from already represented chemistries made the prediction worse. Labelling a few members of the withheld family is the intervention that does work (Section 3.12).

One tempting recommendation is deliberately not made here. We do not suggest gating deployment on a structural-distance threshold, because Section 3.13 shows that such a threshold could not be selected prospectively on this dataset: a threshold chosen on chemistry the model already knows is too permissive for chemistry it does not. The applicability-domain distribution should be *reported*, and a candidate far outside it treated with suspicion. On this dataset a reliable cutoff could not be learned from the standard inner holdouts; candidate-library information or a more adversarial calibration design may therefore be required.

The chemistry-holdout protocol proposed here complements rather than replaces the splitters distributed with PolyMetriX [25]. Its property-extrapolation splitter is reproduced as P4 and is the harshest protocol we ran; its feature-space leave-one-cluster-out splitter partitions the descriptor space, whereas P3 partitions on a chemically named, human-interpretable class, which is what makes the per-class biases in Table 5 directly usable by a synthetic chemist.

### 4.4. Feature-Level Versus Loss-Level Integration of the Group-Contribution Prior

The prior results caution against a symmetric reading, and against a strong one. It is not true that loss-level integration of an approximate formula is always harmful: under class holdout, a prior calibrated in-fold bought $\Delta R^{2}=+0.120$ in 14 of 17 classes, the one positive effect in this study with a nominal Holm-adjusted p below 0.05 (0.033); the evidence we rest on is the effect size, its consistency across classes and its monotone response to injected bias, with the p-value as a secondary summary. Nor is it safe: at λ=1 the same constraint cost −0.583 once its calibration was off by one $\sigma_{y,train}$ (nominal $p_{Holm}=0.003$), and under random splitting it was negative, at λ=1, in every random-split fold at every tested bias level—a qualifier that matters, because at λ=0.1 and 0.3 the same constraint is not uniformly harmful. What the factorial isolates is an asymmetry in sensitivity: as the injected bias grows from zero to one standard deviation, the constrained model’s effect moves by 0.248 (random) to 0.702 (class holdout) while the feature-based model’s moves by at most 0.088. For a first-layer affine map h = W_x x + w_g g + b, replacing g by g + c can be exactly compensated by b becoming b−w_g c, leaving downstream predictions unchanged. The hypothesis class of L3 is invariant to a uniform train-and-test shift in this input. Identical initialisation and finite optimisation need not follow this compensating path, so fitted results can still vary. By contrast, the L1 blended target shifts by λc/(1 + λ). The additive-bias asymmetry is partly built into the formulations. A seed-level analysis of variance would be inappropriate here, because seeds within a fold are not independent; all inference therefore uses the fold as the statistical unit.

The evidence supports a conditional result for a fold-fitted empirical prior, one compact network and uniform additive perturbations. It does not establish that feature-level integration is generally more robust to chemically structured, multiplicative or deployment-only prior errors. It also does not answer whether published Van Krevelen or Bicerano increments should be imposed in the loss. The preferred penalty weight is retrospective rather than independently validated. Supplementary Tables S7 and S8 further delimit the result. External dictionaries, different architectures and independent candidate sets remain necessary tests.

### 4.5. Limitations and Scope

The findings are bounded in several respects, and we state them rather than generalising.

*Two datasets, and only one of them experimental.* The main study rests on a single curated $T_{g}$ table, of which one literature compilation supplies most records, so provenance diversity is limited. The external replication of Section 3.11 shows that property-range extrapolation fails on a second, computed property as badly as it does here, and that the fingerprint-cluster holdout penalty is comparable on both. Generalisation beyond $T_{g}$ rests on that single second dataset.

*The band-gap target is computed, not error-free.* It carries no inter-laboratory experimental scatter, which is why it is useful here, but it carries systematic computational error instead: exchange–correlation functional bias, finite numerical convergence, and the structural idealisation involved in representing a polymer by a single periodic chain without conformational disorder, packing or chain-length effects. A model that reproduced these labels perfectly would not thereby predict a measured band gap.

Class labels are the curators’. Many families share carbon-based motifs, and the vocabulary is not a strict partition of chemical mechanisms. P3 quantifies transfer to these named classes; it is not synonymous with a universally unseen backbone. Polyphosphazenes and polysiloxanes provide the clearest inorganic-backbone examples. Overlap can retain relevant motifs in training, but does not justify calling P3 a general lower bound on chemistry-holdout difficulty.

The group dictionary was designed after inspecting the dataset. Fitting increments within folds prevents coefficient leakage but cannot undo the dataset-informed group choice. Targeted removal of Si and P groups tests one part of this dependence, not the entire design. Validation with a genuinely external or training-only-designed dictionary remains unperformed. The mass-weighted prior fit is also a modelling choice; the Tg-space sensitivity does not retroactively change Table 7 and Table 8.

Few-cluster uncertainty is intrinsically limited. Five folds imply only 32 Rademacher sign vectors and a two-sided sign-flip *p*-value floor of 0.0625. Exhaustive enumeration removes Monte Carlo repetition from the P1/P2 slope audit, but cannot turn five folds into a large independent sample. The 17-class arm avoids that numerical floor but still has overlapping training data; its *p*-values and intervals remain nominal or descriptive.

*The nested abstention gate rests on one inner partition per outer fold.* The threshold is selected on a single deterministic scaffold-grouped inner holdout—the first of four GroupKFold parts of the training partition. Rotating all four inner folds, or aggregating thresholds over several deliberately more adversarial inner partitions such as withheld whole classes, could yield a more stable gate and was not evaluated here. The negative result reported in Section 3.13 is therefore a statement about this inner design, not about abstention in general.

*Class-holdout tests are not tests on independent samples.* The 17 leave-one-class-out models share the great majority of their training data: any two differ only in which single class was withheld, so their paired differences are correlated in a way that neither a sign-flip permutation test nor a class-resampling bootstrap models. Both the class-resampling confidence intervals and the permutation p-values should therefore be read as descriptive or nominal summaries rather than exact repeated-sampling inference. The primary evidence for the prior conclusions is, in order: the paired effect size, the consistency of its direction across classes, the bootstrap interval as a descriptive measure of spread, the monotone response to injected bias, and the penalty-weight sweep, with the nominal p-value last. A reader who discounts $p_{Holm}=0.033$ entirely is left with the same qualitative conclusion, because the effect is monotone in the bias and consistent in sign across 14 of 17 classes. Holding the fitted training composition fixed and permuting model labels on test predictions targets a conditional comparison and requires an exchangeability assumption that is not guaranteed here. It would not account for refitting uncertainty on new training collections. We therefore do not present such a permutation as a correction for leave-one-class-out dependence. Independent external families or repeated refitting under a separately specified training design are needed for stronger inference.

*Model families.* The capacity control of Section 3.3 covers nested-tuned gradient boosting on fused features and a simple ensemble, but no graph neural network or chemical language model [8] was evaluated. What the present controls establish is narrower: within the descriptor-plus-tree family, extra capacity does not close the gap, and additional training data from already represented chemistries stops reducing class-holdout error well before the gap closes. A small labelled calibration set drawn from the genuinely withheld class is a different intervention, and it does help (Section 3.12). Supplementary Table S11 separately reports ridge baselines and an additional unconstrained network under P4. Tree and nearest-neighbour range restrictions cannot be a universal explanation for these models. The uncertainty experiment also uses random forests rather than the headline booster, so calibration findings do not transfer automatically between learners.

$R^{2}$*under narrow holdouts.* For P4 the denominator of $R^{2}$ is the variance within the top $T_{g}$ quintile, which is small; the large negative values should be read as “far worse than predicting the held-out mean”, with MAE and enrichment carrying the interpretable content.

*Prior study design.* The penalty weight was swept (Section 3.10), but the base learner remained a single compact network on stream A, and alternative architectures and gradient-surgery remedies [29] were not explored. The bias was injected additively, which is the simplest miscalibration; multiplicative or chemistry-dependent miscalibration may behave differently, and a real handbook increment set would be wrong in a structured rather than a uniform way. Uniform train-and-test input shifts can be absorbed by the first-layer bias in L3; robustness to this perturbation is partly architectural.

*Abstention was not made to work prospectively.* The nested analysis of Section 3.13 is a negative result on this dataset, not a demonstration that structural gating is impossible. A gate calibrated on an external candidate library, or an inner holdout constructed to be more adversarial than the classes the model has already seen, may well succeed; we did not achieve it here.

*Similarity is not sufficient.* The sample-level slope is reproducible across fingerprints, but similarity explains only 6–11 % of the variance in absolute error inside P1–P3. It is a population-level diagnostic, not a per-candidate error predictor.

*Fingerprint sensitivity covers one descriptor family.* The reference fingerprint and five alternatives were compared—radius 1, 2 and 3, 1024 and 2048 bits, methyl-capped rather than starred repeat units, and a count-based fingerprint compared under the MinMax kernel. All six are Morgan circular fingerprints on the same structures, and each was applied to the whole chemistry-holdout battery—all 5 scaffold folds and all 17 class holdouts—so the comparison is not confined to the chemistries the model happens to handle well. These regressions are a robustness analysis rather than exact repeated-sampling inference: an individual polymer can contribute a held-out prediction under both P2 and P3, and the fold-clustered bootstrap does not explicitly model that cross-protocol dependence, nor the training data the 17 class-holdout folds share. Their agreement shows that the similarity–error relation does not depend on radius, folding width, count-versus-binary encoding or polymerisation-point handling; it is not evidence that the relation would survive a fundamentally different representation such as a learned embedding.

*The variability reference is an estimate on a biased subset.* It rests on 279 polymers that are, by construction, the most-studied ones, and it mixes measurement reproducibility with genuine specimen-to-specimen variation. It is not a floor and bounds nothing. The 279 polymers represent 3.8% of the 7367-record main dataset; their variability and model performance need not represent the less-studied single-source majority. External experimental validation on such structures, ideally in a third independently curated dataset with specimen and measurement metadata, is an important next step.

## 5. Conclusions

The 7367-record study shows that validation protocol substantially changes the reported accuracy of the tested Tg models. For histogram gradient boosting on stream B, random, scaffold, curator-class and high-Tg holdouts give R^2^ = 0.899, 0.857, 0.355 (class-macro) and −3.79, with MAE = 23.9, 28.5, 43.3 and 79.5 K. Random-split accuracy describes interpolation within this collection and should be accompanied by a test matching the intended polymer family and property range. P3 and Butina holdout address different deployment questions; neither is a universal chemistry-holdout benchmark.

Structural similarity provides a limited population-level diagnostic: it explains 6–11% of within-protocol absolute-error variance under P1–P3. Large positive biases for polyphosphazenes and polysiloxanes identify specific transfer failures without establishing a law for all unseen backbones. The single histogram-gradient-boosting model changes from R^2^ = 0.899 to 0.805 for Tg and 0.888 to 0.832 for band gap under matched cluster holdouts; the four-model band-gap mean changes from 0.791 to 0.673. Poor property-range extrapolation is observed for the tested baselines on both properties, but mechanisms and performance remain model-dependent.

Small target-family calibration sets can remove large offsets: five polyphosphazenes reduce MAE from 91.7 to 37.6 K with a constant correction, compared with 64.0 K after retraining on the same five labels. Corrections can add noise when errors are diffuse and mean bias is small. The prospective similarity gate provided little benefit in the tested inner-split design. Random-forest split-conformal empirical coverage was 0.899 under random splitting but only 0.683 and 0.214 under class and property holdout. These are negative stress-test results, not deployment-ready uncertainty or rejection tools.

For the fold-fitted group-contribution prior, the quadratic penalty is equivalent to target shrinkage. Its additive-bias sensitivity differs from that of a network input whose constant shift can be absorbed by a first-layer bias. The result is conditional on architecture, dictionary, fitting criterion and perturbation; it does not establish a general preference for feature-level integration or test handbook increments. All class-holdout inferential results are nominal or descriptive because training sets overlap. Only the variability reference rests on the 279 multiply reported polymers; the main analysis uses 7367 records. That subset is biased towards well-studied polymers and establishes neither an error floor nor the applicability domain for less-studied structures. External validation on those structures and a third independent experimental dataset are needed to extend the conclusions.

## Figures and Tables

**Table 1:** Curation funnel and structural identity audit. The three identity keys are progressively looser; the counts show that residual structural redundancy in the published table is small. Bold type identifies the analysed record count.

Step/Key	Records
Published table	7367
rejected: unparseable structure	0
rejected: not exactly two polymerisation points	0
rejected: $T_{g}$outside 100–900 K	0
Analysed set	**7367**
Distinct PSMILES as written	7367
Distinct canonical isomeric SMILES	7367
Distinct after stereochemistry removal	7366
Distinct InChIKey of the methyl-capped repeat unit	7354
Scaffold groups	3434
Largest scaffold group (fraction of data)	9.5 %
Polymer classes used for leave-one-class-out (n≥40)	17
Polymers with ≥2 independent literature $T_{g}$ values	279
Train/test structure-hash overlap, all 38 folds	0

**Table 2:** Protocol summary. Each P1–P4 entry averages the four non-degenerate baselines (ridge, kNN, random forest, histogram gradient boosting) over all folds of the protocol. “Mean sim.” is the mean maximum Tanimoto similarity of a held-out repeat unit to its own training partition. Enrichment is given with a percentile bootstrap 95 % confidence interval (5000 resamples). The resampling unit is chosen to match each design. For P1 the fold labels have no identity across the three shuffled five-fold repetitions, so the unit is the polymer: polymers are resampled and enrichment is recomputed within every realised test partition before aggregation. P2 resamples the 5 scaffold folds, P3 the 17 held-out classes, and P4 the 1480 candidates in its single deterministic test set. Every interval recomputes the four-baseline mean enrichment on each resample. Class-holdout entries are macro means over the 17 folds. The screening decision is degenerate under P4 because every test point lies above the threshold by construction. All preprocessing is fitted inside the training partition of each fold. The pooled class-holdout reference is histogram gradient boosting (HGB) on stream B, rather than a four-baseline average.

Protocol	Stream	$R^{2}$	MAE (K)	Bal. Acc.	Enrich.	95 % CI	Mean Sim.
P1 random 5-fold (sanity)	A	0.844	31.3	0.912	6.82	6.62–6.99	0.796
	B	0.852	29.6	0.914	7.09	6.89–7.25	0.796
P2 scaffold holdout	A	0.790	36.8	0.902	6.52	6.30–6.74	0.709
	B	0.792	35.5	0.902	6.74	6.53–6.97	0.709
P3 polymer-class holdout	A	0.253	48.9	0.689	4.42	3.79–5.06	0.502
	B	0.121	52.6	0.691	4.30	3.67–4.92	0.502
P4 high-$T_{g}$ extrapolation	A	−5.07	90.3	—	0.49	0.32–0.71	0.680
	B	−5.47	91.7	—	0.83	0.61–1.03	0.680
Class holdout, pooled not macro (HGB, B)	—	0.802	36.6	—	—	—	—
Mean predictor (reference)	—	≤0	95–199	—	—	—	—

**Table 3:** Effect of model capacity on each protocol, for the model in each column averaged over the folds of the protocol (macro; 5 folds for P1 and P2, 17 for P3, 1 for P4). The tuned booster selects its configuration on an inner split of the training fold and uses the fused stream A ⊕ B features; the ensemble averages it with a random forest and a ridge model. The outer test fold is never used for selection. The default model’s random-split MAE is 24.0 K here rather than the 23.9 K reported for the same model in Section 3.2, because this control uses a single repetition of the five-fold split whereas that value averages three; the P2–P4 entries are identical between the two tables. All MAE values are in kelvin.

Protocol	Default $R^{2}$	Default MAE	Tuned $R^{2}$	Tuned MAE	Ens. $R^{2}$	Ens. MAE
P1 random 5-fold	0.898	24.0	0.905	22.3	0.897	24.6
P2 scaffold holdout	0.857	28.5	0.859	28.0	0.859	29.2
P3 class holdout	0.355	43.3	0.361	43.6	0.347	45.1
P4 high-$T_{g}$ extrap.	−3.79	79.5	−3.78	78.4	−4.41	84.9

**Table 4:** Similarity–error relation. The pooled fold-level correlation is confounded by protocol and is reported only descriptively. Within-protocol correlations use the fold as the unit; sample-level models use the individual test prediction as the unit with standard errors clustered on the outer fold for P1–P3, with intervals from a wild cluster bootstrap; P4 has one fold and uses an HC3 interval over its candidates instead. They report the slope of absolute error on maximum Tanimoto similarity. The Descr. p column carries the fold-level correlation p-values, which are descriptive rather than exact repeated-sampling inference: under P3 the 17 leave-one-class-out fits share most of their training data, and under P1 and P2 five folds carry very little power. They occupy their own column because they are not confidence limits. Confidence intervals are reported only for the sample-level slope and fingerprint-sensitivity rows; the pooled and within-protocol fold-level correlations are descriptive summaries and carry none. The fingerprint-sensitivity rows repeat the sample-level model on the whole chemistry-holdout battery: all 5 scaffold folds and all 17 class holdouts, 14,487 predictions in 22 folds. Protocol enters as a fixed effect, so each reported slope is the within-protocol association between similarity and absolute error, and the intervals are the same wild cluster bootstrap used for the sample-level rows, clustered on the fold.

Analysis	Stratum	Estimate	CI Low	CI High	Descr. p	Regression R^2^
Pooled Pearson r (descriptive)	28 folds, all protocols	−0.651	—	—	—	—
Within-protocol Pearson r	P1 random (5 folds)	+0.584	—	—	0.30	—
	P2 scaffold (5 folds)	−0.849	—	—	0.069	—
	P3 class holdout (17 folds)	−0.765	—	—	<0.001	—
	P4 (1 fold)	—	—	—	not estimable	—
Sample-level slope (K per unit similarity)	P1 random	−49.0	−60.6	−37.4	—	0.098
	P2 scaffold	−49.9	−72.3	−27.6	—	0.107
	P3 class holdout	−52.8	−68.6	−35.6	—	0.062
	P4 high-$T_{g}$ (HC3)	−139.3	−152.2	−126.4	—	0.221
Fingerprint sensitivity (K per unit similarity)	Morgan r = 2, 2048 bit	−51.0	−58.7	−43.5	—	0.097
	Morgan r = 3, 2048 bit	−48.6	−55.6	−41.9	—	0.095
	Morgan r = 2, 1024 bit	−51.8	−59.6	−44.1	—	0.097
	Morgan r = 2, methyl-capped	−51.7	−59.5	−44.2	—	0.097
	Morgan r = 1, 2048 bit	−58.0	−67.6	−47.9	—	0.092
	Morgan r = 2, count/MinMax	−58.1	−67.9	−48.8	—	0.098

**Table 5:** Leave-one-polymer-class-out results for histogram gradient boosting on stream B. Six representative classes are shown, including the best-predicted class, the largest class, strongly over-predicted inorganic-backbone families, an under-predicted class, and a class whose error is diffuse rather than offset. Bias is the mean signed error (prediction minus experiment) on the withheld class. The complete 17-class table is Supplementary Table S3.

Withheld Class	n	MAE (K)	$R^{2}$	Bias (K)	Class Mean $T_{g}$ (K)
Polyesters	660	28.6	0.79	−3.4	365.9
Polyimides	1765	33.1	0.67	−5.4	513.8
Polyamides	486	40.1	0.69	−18.5	433.3
Polysiloxanes	238	44.4	0.26	+33.9	271.5
Polyvinyls	254	48.6	0.32	−3.2	347.2
Polyphosphazenes	107	91.7	−2.56	+91.1	260.1
Macro mean over all 17 classes	—	43.3	0.355	—	—
Pooled over all 17 classes	7120	36.6	0.802	—	—
Random-split reference (same model)	7367	23.9	0.899	—	417.1

**Table 6:** Unsupervised cluster-holdout sensitivity on the $T_{g}$ dataset. Whole-cluster five-fold holdout of Butina clusters of the 2048-bit Morgan fingerprints, with greedy fold size balancing; histogram gradient boosting on stream B, macro means over the five folds. Random-split and curator-class rows are the same model under P1 and P3 for comparison. The band gap under the identical Butina construction is not repeated here; the matched cross-dataset comparison is presented in Section 3.11.

Partition	Clusters	Largest	$R^{2}$	MAE (K)	Spearman	Enrichment	Mean Sim.
Butina cutoff 0.50	1395	6.4 %	0.854	30.4	0.921	7.23	0.616
Butina cutoff 0.65	603	17.3 %	0.805	31.3	0.886	7.06	0.612
Butina cutoff 0.80	134	43.8 %	0.772	33.7	0.870	6.74	0.575
Random five-fold (P1)	—	—	0.899	23.9	—	7.75	0.796
Curator-class holdout (P3, macro)	—	—	0.355	43.3	—	5.25	0.502

**Table 7:** Paired change in test $R^{2}$ relative to the L0 baseline on the same fold, for the group-contribution prior enforced as a loss constraint (L1) or supplied as an input feature (L3), as a function of injected calibration bias δ, expressed in units of the outer-training target standard deviation $\sigma_{y,train}$. The statistical unit is the fold: the three seeds of a fold are averaged before the paired difference is taken, so each cell rests on n=5 folds under P1 and n=17 under P3. CIs are percentile bootstrap over folds; “Sign” is the number of folds in which the effect has the sign of the mean. $p_{Holm}$ is a two-sided sign-flip permutation p-value with Holm correction over the eight contrasts of each protocol. Its minimum attainable value is $2/2^{n}$: *0.0625* on five folds, so no random-split cell can attain nominal p<0.05 and none is claimed; $1.5\times 10^{-5}$ on 17 folds, so the class-holdout cells can be tested, and two reach a nominal Holm-adjusted p<0.05 (bold). Because the 17 leave-one-class-out fits share most of their training data, both these p-values and the class-resampling intervals are nominal summaries rather than exact repeated-sampling inference.

Protocol	Strategy	δ	$\Delta R^{2}$	CI Low	CI High	Sign	$p_{Holm}$
Random 5-fold	L1	0.00	−0.0297	−0.0351	−0.0244	5/5	0.488
	L1	0.25	−0.0434	−0.0492	−0.0373	5/5	0.488
	L1	0.50	−0.0925	−0.0986	−0.0866	5/5	0.488
	L1	1.00	−0.2772	−0.2912	−0.2632	5/5	0.488
	L3	0.00	+0.0047	+0.0027	+0.0068	5/5	0.488
	L3	0.25	−0.0001	−0.0020	+0.0018	3/5	1.000
	L3	0.50	−0.0015	−0.0062	+0.0033	4/5	1.000
	L3	1.00	+0.0022	+0.0004	+0.0042	4/5	0.488
Polymer-class holdout	L1	0.00	+0.1196	+0.0506	+0.1976	14/17	**0.033**
	L1	0.25	+0.0607	−0.0333	+0.1560	11/17	1.000
	L1	0.50	−0.1216	−0.2906	+0.0296	11/17	1.000
	L1	1.00	−0.5828	−0.9284	−0.2820	16/17	0.003
	L3	0.00	−0.0088	−0.0943	+0.0535	9/17	1.000
	L3	0.25	−0.0108	−0.0483	+0.0237	12/17	1.000
	L3	0.50	−0.0555	−0.2067	+0.0424	8/17	1.000
	L3	1.00	−0.0966	−0.3148	+0.0330	8/17	1.000

**Table 8:** Penalty-weight sweep for the loss-constrained prior. Entries are the paired $\Delta R^{2}$ against the L0 baseline on the same fold. The figure in parentheses is the number of folds in which the effect is positive, out of five under random splitting and out of 17 under class holdout. Seeds are averaged within a fold before the paired difference is taken. The λ = 1 entries are the same factorial runs as Table 7: identical outer folds, three initialisation seeds, preprocessing and L0 references. All 132 corresponding per-seed R^2^ and MAE records agree exactly.

Protocol	Prior	λ=0.1	0.3	1	3	10
Random 5-fold	calibrated	+0.005 (5)	+0.001 (4)	−0.030 (0)	−0.086 (0)	−0.140 (0)
	$\delta =0.5\sigma_{y,train}$	+0.004 (5)	−0.011 (0)	−0.093 (0)	−0.242 (0)	−0.365 (0)
Class holdout	calibrated	+0.006 (10)	+0.118 (15)	+0.120 (14)	+0.002 (10)	−0.163 (6)
	$\delta =0.5\sigma_{y,train}$	+0.035 (10)	+0.033 (10)	−0.122 (6)	−0.459 (3)	−0.837 (0)

**Table 9:** External replication on the DFT chain band gap of 4127 polymers (polyVERSE). Entries average the four non-degenerate baselines over the folds of each protocol; MAE is in eV and is also given normalised by the target standard deviation so that it can be compared with the $T_{g}$ study directly. E3 uses whole-cluster holdout of Butina clusters at a Tanimoto-distance cutoff of 0.65, the same construction as Table 6. The four $T_{g}$ comparison rows are matched to the band-gap protocol above them construction by construction: the third is the $T_{g}$ Butina cluster holdout at the same 0.65 cutoff, not the curator-class holdout, which is a different partition and is reported separately in Table 2 and Figure 12. All four are four-baseline means over the folds of their protocol. Figure 12 instead shows histogram gradient boosting alone. Its values must not be substituted for the four-model averages in this table. The italic row label identifies the Tg comparison block.

Protocol	$R^{2}$	MAE (eV)	Normalised MAE	Enrichment	Mean Sim.
E1 random 5-fold (sanity)	0.791	0.431	0.299	6.97	0.716
E2 scaffold holdout	0.666	0.527	0.366	6.19	0.632
E3 cluster-grouped five-fold	0.673	0.534	0.371	5.56	0.555
E4 high-band-gap extrapolation	−5.62	1.094	0.759	0.54	0.629
$T_{g}$ *(stream B), matched construction*					
random 5-fold	—	—	0.263	—	—
scaffold holdout	—	—	0.315	—	—
Butina cluster holdout, cutoff 0.65	—	—	0.355	—	—
high-$T_{g}$ extrapolation	—	—	0.814	—	—

**Table 10:** Few-shot class calibration by retraining. MAE (K) on the remaining members of the withheld class after k of its members are added to the training set, averaged over five random draws. Dashes mark cells that the reporting rule of Section 2.13 excludes, because k would leave fewer than about a third of the class as test data; the withheld cells and their remaining test sizes are listed in fewshot_reporting_audit.csv. The random-split reference for this model is *23.9* K.

Withheld Class	n	k=0	5	10	25	50	100
Polyphosphazenes	107	91.7	64.0	52.4	34.8	28.7	—
Polyvinyls	254	48.6	46.3	45.9	44.0	41.9	39.5
Polyhalo-olefins	40	48.3	47.1	40.5	51.3	—	—
Polysiloxanes	238	44.4	43.0	40.5	36.5	32.1	28.3
Polystyrenes	241	42.4	40.1	38.2	37.1	32.1	28.7
Polyacrylics	617	34.9	35.0	34.0	35.0	32.3	30.1
Polyoxides	1748	33.4	33.4	33.0	32.6	32.3	31.1
Polyimides	1765	33.1	33.3	32.9	32.7	31.6	31.4
Polyesters	660	28.6	28.6	28.3	27.7	27.2	25.4

**Table 11:** What the k labels are actually buying. MAE (K) on the remaining members of the withheld class when the same k labelled members are used to retrain the model, to fit a constant offset, or to fit a linear recalibration; “none” is the zero-shot model. Means over five random draws. Where the zero-shot failure is a large offset (polyphosphazenes, polysiloxanes) a one-parameter correction beats retraining at small k; where it is diffuse (polyesters, polyvinyls) both corrections make the prediction worse.

Withheld Class	Treatment	k=5	10	25	50	100
Polyphosphazenes (bias +91 K)	none	91.7	92.7	92.1	92.4	—
	offset	37.6	38.3	36.9	37.4	—
	linear	32.6	31.3	29.7	28.7	—
	retrain	64.0	52.4	34.8	28.7	—
Polysiloxanes (bias +34 K)	none	44.6	44.3	44.5	44.4	45.0
	offset	34.5	36.0	32.1	31.8	33.9
	linear	38.2	36.9	32.0	31.1	33.1
	retrain	43.0	40.5	36.5	32.1	28.3
Polyvinyls (bias −3 K, diffuse)	none	48.4	48.6	48.5	48.2	49.2
	offset	51.1	51.6	49.7	50.2	50.0
	linear	62.4	54.0	49.9	51.7	50.5
	retrain	46.3	45.9	44.0	41.9	39.5
Polyesters (bias −3 K, already good)	none	28.6	28.6	28.6	28.6	28.3
	offset	32.9	30.0	29.0	28.8	28.6
	linear	33.3	31.0	29.0	29.2	28.1
	retrain	28.6	28.3	27.7	27.2	25.4

**Table 12:** Interval calibration by protocol, macro means over folds, for a 300-tree random forest on stream B. “Tree dispersion” intervals are μ±1.645 s, with s the spread of the individual tree predictions; they are uncalibrated and the *0.90* target is nominal only in the sense of the Gaussian multiplier used. “Split conformal” intervals use an absolute-residual conformity score at α=0.10, calibrated on a held-out 20 % of each outer training partition that is disjoint from the test fold and, under P3, contains no member of the withheld class. MAE and both widths are in kelvin; r is the Pearson correlation between the reported tree spread and the realised absolute error. Bold coverage values highlight severe undercoverage under P4.

Protocol	MAE	Tree Cov.	Tree Wid.	Conf. Cov.	Conf. Wid.	r
P1 random 5-fold	25.3	0.967	148.6	0.899	118.7	0.46
P2 scaffold holdout	31.2	0.962	170.9	0.851	118.7	0.42
P3 class holdout	47.0	0.931	217.0	0.683	116.3	0.26
P4 high-$T_{g}$ extrap.	95.7	**0.238**	123.5	**0.214**	103.4	0.58

**Table 13:** Empirical split-conformal coverage by maximum Tanimoto similarity. Included *n* is the total number of test predictions in eligible fold–stratum cells, and Folds is their count. Coverage is the unweighted mean over eligible cells. Cells with fewer than 10 polymers were omitted by the archived calculation. Under P3 the strata use 16, 17, 12 and 8 folds; the original mean counts were therefore not additive. Included P3 counts total 7093 of 7120 held-out polymers, with 27 in omitted small cells. P1, P2 and P4 have no omitted cells. These eligibility-conditioned estimates are not all-candidate conditional coverage.

Similarity	Statistic	P1 Random	P2 Scaffold	P3 Class	P4 High Tg
Below 0.4	Included *n*	145	505	1316	54
Below 0.4	Folds	5	5	16	1
Below 0.4	Coverage	0.685	0.609	0.613	0.000
0.4–0.6	Included *n*	904	1896	3333	436
0.4–0.6	Folds	5	5	17	1
0.4–0.6	Coverage	0.762	0.755	0.670	0.025
0.6–0.8	Included *n*	2399	2180	1793	615
0.6–0.8	Folds	5	5	12	1
0.6–0.8	Coverage	0.884	0.872	0.798	0.202
0.8–1.0	Included *n*	3919	2786	651	375
0.8–1.0	Folds	5	5	8	1
0.8–1.0	Coverage	0.948	0.942	0.818	0.483

## Data Availability

The curated glass-transition dataset is openly available from Zenodo at https://doi.org/10.5281/zenodo.15210035 under CC-BY-4.0 [26] and is distributed with PolyMetriX [25]. The DFT chain band-gap dataset is available in polyVERSE at https://doi.org/10.5281/zenodo.13352644 [32]. Derived source tables, out-of-fold predictions and analysis code are included in the accompanying reproducibility archive; revision-specific scripts and numerical outputs are identified separately. Input-file digests are retained in the original reproduction logs.

## Supplementary Materials

The following supporting information can be downloaded at: https://www.mdpi.com/article/10.3390/polym18192445/s1, Supplementary_Materials.docx consolidates Tables S1–S20 and explanatory notes in one document. Reproducibility_Archive.zip preserves the machine-readable source tables, predictions, original reproduction package, numerical audit and executed revision analyses. The supplementary tables are: Table S1. Group dictionary and support; Table S2. Fold preprocessing and leakage audit; Table S3. Complete class holdout results; Table S4. Protocol summary behind Figure 2; Table S5. Normalised split conformal results; Table S6. Single model and four model comparisons; Table S7. Targeted dictionary sensitivity; Table S8. Sensitivity to prior fitting space; Table S9. Exhaustive five fold wild sign audit; Table S10. Signed bias as the training pool grows; Table S11. High Tg extrapolation by model; Table S12. Conditional coverage denominators; Table S13. Prior integration factorial summaries; Table S14. Penalty sweep summaries; Table S15. Learning curve summary; Table S16. Matched few shot correction results; Table S17. Prospective abstention results; Table S18. Fingerprint sensitivity; Table S19. Band gap results by model; Table S20. Between source variability reference. The tables are intended for reading; the archive provides the underlying records and code.
