# Supplementary material for: What Random Splits Measure in Polymer Glass-Transition Prediction: Chemistry Holdouts, Applicability Domains, and Prior Integration
Source: Polymers (Basel). 2026 Oct 7;18(19):2445. doi: 10.3390/polym18192445 (PMC13667063; doi:10.3390/polym18192445)
Supplement: Supplementary file 1 [file polymers-18-02445-s001.zip › polymers-4543851-supplementary.pdf]

Supplementary Materials

What Random Splits Measure in Polymer Glass-Transition Prediction: Chemistry Holdouts, Applicability Domains, and Prior Integration

This document consolidates additional tables and audit explanations for the revised manuscript. S1–S5 retain their original roles; S6–S12 document reviewer-requested checks and sensitivity analyses; S13–S20 collect the remaining result summaries. Full per-run records, predictions and code are retained in Reproducibility_Archive.zip. Averages are descriptive unless a specific inferential procedure is stated. Tg errors are in kelvin; band-gap errors are in electronvolts.

Table S1 Group dictionary and support

Source: smarts_group_table.csv. Groups may overlap. Counts are structural occurrences, not exclusive chemical assignments. All 35 groups have nonzero support in all 22 prior-analysis training folds. Coefficient identifiability is not guaranteed.

group id	name	smarts	chemical meaning
G01	CH3	[CX4H3]	terminal methyl
G02	CH2	[CX4H2]	methylene
G03	CH	[CX4H1]	methine
G04	C_quat	[CX4H0]	quaternary sp3 carbon
G05	CH=CH	[CX3H1]=[CX3H1]	disubstituted alkene
G06	C=C_subst	[CX3H0]=[CX3]	substituted alkene
G07	phenylene	c1ccccc1	benzene ring
G08	arom_C	[c]	aromatic carbon (atom count)
G09	arom_N	[n]	aromatic nitrogen
G10	ether_O	[OX2;!$(O[CX3]=O)]	ether oxygen, excluding ester oxygen
G11	ester	[CX3](=O)[OX2][#6]	carboxylic ester
G12	carbonate	[OX2][CX3](=O)[OX2]	carbonate
G13	ketone	[#6][CX3](=O)[#6]	ketone
G14	amide	[CX3](=O)[NX3]	amide
G15	imide	[NX3]([CX3]=O)[CX3]=O	imide
G16	urethane	[NX3][CX3](=O)[OX2]	urethane
G17	urea	[NX3][CX3](=O)[NX3]	urea
G18	anhydride	[CX3](=O)[OX2][CX3]=O	anhydride
G19	amine_N	[NX3;!$(NC=O)]	amine nitrogen, excluding amide
G20	imine	[CX3]=[NX2]	imine
G21	nitrile	[NX1]#[CX2]	nitrile
G22	nitro	[N+](=O)[O-]	nitro group
G23	hydroxyl	[OX2H]	hydroxyl
G24	thioether_S	[SX2]	divalent sulfur
G25	sulfone	S(=O)(=O)	sulfone
G26	Si	[Si]	silicon
G27	P	[P]	phosphorus
G28	F	[F]	fluorine
G29	Cl	[Cl]	chlorine
G30	Br_I	[Br,I]	bromine or iodine
G31	ring_aliph	[CX4;R]	sp3 carbon in a ring

group id	name	smarts	chemical meaning
G32	ring_fused_arom	c1ccc2ccccc2c1	fused aromatic ring
G33	CF3	[CX4](F)(F)F	trifluoromethyl
G34	t_butyl	[CX4H0]([CH3])([CH3])[CH3]	tert-butyl
G35	biphenyl_link	c1ccc(-c2ccccc2)cc1	biphenyl

group id	n polymers with group	folds with nonzero support	folds with nonzero increment	increment full data K g per mol
G01	3421	22	22	6910.978
G02	4682	22	22	2642.267
G03	2032	22	22	2144.707
G04	2431	22	13	75.124
G05	347	22	22	6651.526
G06	244	22	22	12157.549
G07	5922	22	0	0.000
G08	6021	22	22	7132.941
G09	984	22	22	14316.207
G10	4074	22	1	0.000
G11	2412	22	22	6949.431
G12	147	22	3	0.000
G13	532	22	22	5023.900
G14	3047	22	22	21697.186
G15	1777	22	2	0.000
G16	213	22	0	0.000
G17	72	22	0	0.000
G18	35	22	22	15312.213
G19	1261	22	22	6008.590
G20	104	22	0	0.000
G21	303	22	22	10045.505
G22	188	22	22	5758.827
G23	268	22	22	6709.278
G24	267	22	22	5008.163
G25	501	22	22	30498.639
G26	433	22	1	0.000
G27	349	22	22	7408.555
G28	967	22	22	7223.842
G29	195	22	22	16581.140
G30	116	22	22	34964.007
G31	1142	22	22	5166.026
G32	384	22	20	812.399
G33	770	22	22	16607.550
G34	189	22	22	9178.086
G35	1080	22	0	0.000

Table S2 Fold preprocessing and leakage audit

Source: audit_preprocessing_leakage.csv. Each partition is shared by the two streams, so fold sizes and zero overlap are listed once. The archive retains both stream-specific audit records and full hashes. Imputers and scalers are fitted on training rows only.

protocol	fold	n train	n test	train test overlap
P1_random	rep0_fold0	5893	1474	0
P1_random	rep0_fold1	5893	1474	0
P1_random	rep0_fold2	5894	1473	0
P1_random	rep0_fold3	5894	1473	0
P1_random	rep0_fold4	5894	1473	0
P1_random	rep1_fold0	5893	1474	0
P1_random	rep1_fold1	5893	1474	0
P1_random	rep1_fold2	5894	1473	0
P1_random	rep1_fold3	5894	1473	0
P1_random	rep1_fold4	5894	1473	0
P1_random	rep2_fold0	5893	1474	0
P1_random	rep2_fold1	5893	1474	0
P1_random	rep2_fold2	5894	1473	0
P1_random	rep2_fold3	5894	1473	0
P1_random	rep2_fold4	5894	1473	0
P2_scaffold	fold0	5893	1474	0
P2_scaffold	fold1	5893	1474	0
P2_scaffold	fold2	5894	1473	0
P2_scaffold	fold3	5894	1473	0
P2_scaffold	fold4	5894	1473	0
P3_class_holdout	Polyimides	5602	1765	0
P3_class_holdout	Polyoxides	5619	1748	0
P3_class_holdout	Polyesters	6707	660	0
P3_class_holdout	Polyacrylics	6750	617	0
P3_class_holdout	Polyamides	6881	486	0
P3_class_holdout	Polyimines	7102	265	0
P3_class_holdout	Polyvinyls	7113	254	0
P3_class_holdout	Polystyrenes	7126	241	0
P3_class_holdout	Polysiloxanes	7129	238	0
P3_class_holdout	Polysulfides	7151	216	0
P3_class_holdout	Polycarbonates	7207	160	0
P3_class_holdout	Polyurethanes	7244	123	0
P3_class_holdout	Polyphosphazenes	7260	107	0
P3_class_holdout	Polyphenylenes	7263	104	0
P3_class_holdout	Polyolefins	7312	55	0
P3_class_holdout	Polydienes	7326	41	0
P3_class_holdout	Polyhalo-olefins	7327	40	0
P4_highTg_extrap	train_Tg_lt_524.1K	5887	1480	0

stream	input features	minimum kept	maximum kept	maximum missing
A_published_descriptors	100	99	99	0
B_morgan_rdkit	1034	1034	1034	0

Table S3 Complete class holdout results

Source: table_class_holdout.csv. Histogram gradient boosting on stream B. Bias uses prediction minus experiment. Rows are classes, not independent replications of training.

class	n	MAE	R2	bias	Tg mean
Polyesters	660	28.622	0.788	-3.433	365.930
Polycarbonates	160	29.820	0.765	-9.099	402.343
Polyimides	1765	33.051	0.668	-5.415	513.804
Polyoxides	1748	33.380	0.815	0.391	417.891
Polyacrylics	617	34.855	0.474	-7.536	341.384
Polyurethanes	123	35.912	0.209	10.226	347.457
Polyamides	486	40.062	0.688	-18.518	433.248
Polyphenylenes	104	41.269	0.452	-6.537	407.902
Polystyrenes	241	42.416	0.343	-17.165	370.082
Polyimines	265	42.726	0.792	-7.467	441.580
Polysulfides	216	43.434	0.534	24.135	353.860
Polysiloxanes	238	44.402	0.256	33.899	271.520
Polyolefins	55	46.092	0.671	7.584	305.260
Polyhalo-olefins	40	48.310	0.217	-31.989	331.000
Polyvinyls	254	48.623	0.322	-3.213	347.238
Polydienes	41	51.215	0.613	19.575	319.816
Polyphosphazenes	107	91.667	-2.562	91.071	260.142

Table S4 Protocol summary behind Figure 2

Source: figure2_source_data.csv. Model dispersions describe variation among the four non-degenerate baselines, rather than confidence intervals across folds.

protocol	stream	metric	mean	sd across baselines	n baselines
P1_random	A_published_descriptors	R2	0.844	0.051	4
P1_random	A_published_descriptors	MAE	31.334	6.638	4
P1_random	A_published_descriptors	enrichment_top10pct	6.817	0.797	4
P1_random	B_morgan_rdkit	R2	0.852	0.051	4
P1_random	B_morgan_rdkit	MAE	29.601	5.809	4
P1_random	B_morgan_rdkit	enrichment_top10pct	7.093	0.685	4
P2_scaffold	A_published_descriptors	R2	0.790	0.045	4
P2_scaffold	A_published_descriptors	MAE	36.830	4.942	4
P2_scaffold	A_published_descriptors	enrichment_top10pct	6.519	0.685	4
P2_scaffold	B_morgan_rdkit	R2	0.792	0.076	4
P2_scaffold	B_morgan_rdkit	MAE	35.474	6.923	4
P2_scaffold	B_morgan_rdkit	enrichment_top10pct	6.737	0.721	4
P3_class_holdout	A_published_descriptors	R2	0.253	0.086	4
P3_class_holdout	A_published_descriptors	MAE	48.915	3.253	4
P3_class_holdout	A_published_descriptors	enrichment_top10pct	4.418	0.350	4
P3_class_holdout	B_morgan_rdkit	R2	0.121	0.263	4
P3_class_holdout	B_morgan_rdkit	MAE	52.579	9.367	4
P3_class_holdout	B_morgan_rdkit	enrichment_top10pct	4.298	0.824	4
P4_highTg_extrap	A_published_descriptors	R2	-5.073	0.535	4
P4_highTg_extrap	A_published_descriptors	MAE	90.337	3.992	4
P4_highTg_extrap	A_published_descriptors	enrichment_top10pct	0.490	0.272	4
P4_highTg_extrap	B_morgan_rdkit	R2	-5.469	1.703	4
P4_highTg_extrap	B_morgan_rdkit	MAE	91.713	12.323	4
P4_highTg_extrap	B_morgan_rdkit	enrichment_top10pct	0.828	0.674	4

Table S5 Normalised split conformal results

Source: supp_table_S5_normalised_conformal.csv and its conditional companion. The normalised conformity score divides residuals by the forest spread. The calculation uses the archived interpolated quantile. Conditional cells with fewer than 10 test polymers are excluded; averages refer only to eligible cells.

protocol	n folds	unnorm cov	unnorm width	norm cov
P1_random	5.000	0.899	118.694	0.900
P2_scaffold	5.000	0.851	118.687	0.877
P3_class_holdout	17.000	0.683	116.336	0.790
P4_highTg_extrap	1.000	0.214	103.385	0.057

protocol	norm width
P1_random	106.793
P2_scaffold	120.149
P3_class_holdout	148.829
P4_highTg_extrap	88.530

protocol	stratum	conformal abs coverage	conformal norm coverage
P1_random	tanimoto_0.0-0.4	0.685	0.802
P1_random	tanimoto_0.4-0.6	0.762	0.846
P1_random	tanimoto_0.6-0.8	0.884	0.908
P1_random	tanimoto_0.8-1.01	0.948	0.911
P2_scaffold	tanimoto_0.0-0.4	0.609	0.773
P2_scaffold	tanimoto_0.4-0.6	0.755	0.846
P2_scaffold	tanimoto_0.6-0.8	0.872	0.893
P2_scaffold	tanimoto_0.8-1.01	0.942	0.904
P3_class_holdout	tanimoto_0.0-0.4	0.613	0.755
P3_class_holdout	tanimoto_0.4-0.6	0.670	0.788
P3_class_holdout	tanimoto_0.6-0.8	0.798	0.849
P3_class_holdout	tanimoto_0.8-1.01	0.818	0.809
P4_highTg_extrap	tanimoto_0.0-0.4	0.000	0.000
P4_highTg_extrap	tanimoto_0.4-0.6	0.025	0.007
P4_highTg_extrap	tanimoto_0.6-0.8	0.202	0.055
P4_highTg_extrap	tanimoto_0.8-1.01	0.483	0.128

Table S6 Single model and four model comparisons

Figure 12 uses histogram gradient boosting; Table 9 uses equal-weight means of four non-degenerate baselines after fold aggregation. These are different estimands. Sources: external_scores.csv, figure12_like_for_like.csv and table9_tg_comparison_source.csv. Normalised MAE divides by the full target standard deviation used for descriptive cross-property comparison.

Aggregation	protocol	R2	MAE	MAE / SD
HistGBT	E1_random	0.888	0.334	0.232
HistGBT	E2_scaffold	0.837	0.397	0.276
HistGBT	E3_cluster_holdout	0.832	0.393	0.273
HistGBT	E4_high_extrap	-3.437	0.877	0.609
Four-model mean	E1_random	0.791	0.431	0.299
Four-model mean	E2_scaffold	0.666	0.527	0.366
Four-model mean	E3_cluster_holdout	0.673	0.534	0.371
Four-model mean	E4_high_extrap	-5.622	1.094	0.760

protocol	dataset	construction	R2	MAE over sigma y
Random	Tg (experimental)	matched	0.899	0.212
Scaffold	Tg (experimental)	matched	0.857	0.253
Cluster, Butina 0.65	Tg (experimental)	matched	0.805	0.277
Property extrap.	Tg (experimental)	matched	-3.788	0.706
Random	Chain band gap (DFT)	matched	0.888	0.232
Scaffold	Chain band gap (DFT)	matched	0.837	0.276
Cluster, Butina 0.65	Chain band gap (DFT)	matched	0.832	0.273
Property extrap.	Chain band gap (DFT)	matched	-3.437	0.609
Named-class holdout	Tg (experimental)	Tg only, not matched	0.355	0.384

pair	Tg MAE K	Tg MAE over sy	n rows
E1 random / Tg random	29.601	0.263	20
E2 scaffold / Tg scaffold	35.474	0.315	20
E3 Butina 0.65 / Tg Butina 0.65	39.956	0.355	20
E4 extrap / Tg high-Tg extrap	91.713	0.814	4

Table S7 Targeted dictionary sensitivity

New analysis. The Si and P elemental count columns (zero-based columns 25 and 26) were removed together and all remaining increments refitted on training rows. Both inorganic-backbone holdouts use the original cached stream A, the original 64–64 network, 150 epochs, λ=1 and three seeds, at δ=0 and 1. Full-dictionary controls reproduce all 30 matching original per-run records to numerical precision (maximum absolute R^2 difference below 2×10^{-14}). This tests two explicit groups, not all dataset-informed design choices. It is not an independent external-dictionary validation.

fold	dictionary	integration	bias delta	R2	MAE
Polyphosphazenes	full	L0	0.000	-0.886	54.859
Polyphosphazenes	full	L1	0.000	-0.655	54.464
Polyphosphazenes	full	L1	1.000	-3.009	90.938
Polyphosphazenes	full	L3	0.000	-1.449	62.171
Polyphosphazenes	full	L3	1.000	-2.550	75.480
Polyphosphazenes	omit_Si_P	L1	0.000	-0.391	50.562
Polyphosphazenes	omit_Si_P	L1	1.000	-2.535	84.947
Polyphosphazenes	omit_Si_P	L3	0.000	-1.100	59.801
Polyphosphazenes	omit_Si_P	L3	1.000	-1.364	61.690
Polysiloxanes	full	L0	0.000	-0.292	57.012
Polysiloxanes	full	L1	0.000	-0.225	58.569
Polysiloxanes	full	L1	1.000	-1.969	97.581
Polysiloxanes	full	L3	0.000	-0.107	51.404
Polysiloxanes	full	L3	1.000	-0.121	53.632
Polysiloxanes	omit_Si_P	L1	0.000	-0.241	58.798
Polysiloxanes	omit_Si_P	L1	1.000	-2.028	98.284
Polysiloxanes	omit_Si_P	L3	0.000	0.026	49.110
Polysiloxanes	omit_Si_P	L3	1.000	-0.032	51.995

Table S8 Sensitivity to prior fitting space

New prior-only analysis on the original five random and 17 class holdouts. The original fit minimises $\Sigma(N_iY - M_iTg_i)^2$, giving M_i^2 weights to squared Tg error. The alternative fits N_i/M_i directly to Tg_i with nonnegative increments. These are paired changes to the prior alone; the full L1/L3 factorial was not repeated with the alternative fitting criterion.

protocol	fit target	R2	MAE
P1_random	Tg_space	0.692	46.710
P1_random	mass_weighted	0.670	48.093
P3_class_holdout	Tg_space	0.017	55.130
P3_class_holdout	mass_weighted	-0.047	56.981

Table S9 Exhaustive five fold wild sign audit

New enumeration of all 32 equally weighted Rademacher sign vectors for each of P1 and P2. The fitted residual wild-bootstrap and cluster-corrected covariance match the archived procedure. Discrete inverse-CDF quantiles are used, avoiding artificial interpolation of this finite distribution. This removes Monte Carlo duplication, not the limitations of five folds or overlapping training sets. The endpoints round to the original Table 4 values.

protocol	slope	ci low	ci high	distinct sign vectors
P1_random	-48.985	-60.569	-37.402	32
P2_scaffold	-49.946	-72.286	-27.607	32

Table S10 Signed bias as the training pool grows

New signed-bias outputs reproduce the original polyphosphazene learning-curve sampling and model settings. Test set: the same 107 polyphosphazenes. Each smaller fraction uses three original subsampling seeds; 100% is one full-data fit. Bias is prediction minus experiment. The increase after 25% is a descriptive observation for this one family, not a general causal law.

frac	n train	runs	bias mean	bias SD	MAE mean
0.100	726.000	3.000	77.490	10.577	79.697
0.250	1815.000	3.000	63.732	11.075	65.478
0.500	3630.000	3.000	70.260	6.040	72.337
0.750	5445.000	3.000	78.067	5.687	79.646
1.000	7260.000	1.000	91.071	—	91.667

Table S11 High Tg extrapolation by model

Archived P4 results for both streams and all baselines, followed by a new unconstrained L0 neural-network check on stream A using three original initialisation seeds. The network is not included in the four-model means. All use the lower-80% training rule and 1480 top-range test polymers. Range saturation is not assumed for ridge or the network. Large negative R^2 is sensitive to the narrow test-target variance; MAE and ranking metrics should be read alongside it.

stream	model	R2	MAE	bias K	enrichment top10pct
A_published_descriptors	Dummy_mean	-22.053	199.151	-199.151	0.541
A_published_descriptors	Ridge	-4.995	89.269	-87.927	0.608
A_published_descriptors	kNN_k5	-5.584	93.111	-93.111	0.203
A_published_descriptors	RandomForest	-5.355	93.816	-93.816	0.338
A_published_descriptors	HistGBT	-4.357	85.152	-85.152	0.811
B_morgan_rdkit	Dummy_mean	-22.053	199.151	-199.151	0.541
B_morgan_rdkit	Ridge	-4.695	84.497	-82.417	1.149
B_morgan_rdkit	kNN_k5	-7.761	107.183	-107.183	0.608
B_morgan_rdkit	RandomForest	-5.632	95.653	-95.653	0.000
B_morgan_rdkit	HistGBT	-3.788	79.520	-79.514	1.554

seed	R2	MAE	bias K	enrichment	pred max
0.000	-5.235	89.631	-88.707	1.689	679.926
1.000	-4.382	78.738	-77.550	1.014	621.838
2.000	-4.413	81.641	-80.123	2.095	668.666

Table S12 Conditional coverage denominators

Source: uncertainty_conditional.csv. Each row counts the fold–stratum cells retained by the n≥10 rule. The P3 included counts total 7093; the 27 remaining predictions lie in excluded small cells. The original 82+196+149+81 combined four means with denominators 16, 17, 12 and 8 and was not a count of 508 polymers per fold. Coverage retains its original unweighted eligible-cell definition.

protocol	stratum	eligible folds	included n	coverage
P1_random	tanimoto_0.0-0.4	5	145	0.685
P1_random	tanimoto_0.4-0.6	5	904	0.762
P1_random	tanimoto_0.6-0.8	5	2399	0.884
P1_random	tanimoto_0.8-1.01	5	3919	0.948
P2_scaffold	tanimoto_0.0-0.4	5	505	0.609
P2_scaffold	tanimoto_0.4-0.6	5	1896	0.755
P2_scaffold	tanimoto_0.6-0.8	5	2180	0.872
P2_scaffold	tanimoto_0.8-1.01	5	2786	0.942
P3_class_holdout	tanimoto_0.0-0.4	16	1316	0.613
P3_class_holdout	tanimoto_0.4-0.6	17	3333	0.670
P3_class_holdout	tanimoto_0.6-0.8	12	1793	0.798
P3_class_holdout	tanimoto_0.8-1.01	8	651	0.818
P4_highTg_extrap	tanimoto_0.0-0.4	1	54	0.000
P4_highTg_extrap	tanimoto_0.4-0.6	1	436	0.025
P4_highTg_extrap	tanimoto_0.6-0.8	1	615	0.202
P4_highTg_extrap	tanimoto_0.8-1.01	1	375	0.483

Table S13 Prior integration factorial summaries

Source: physics_runs_allclasses.csv. Three seeds are averaged within each fold; folds then receive equal weight. Mean changes are paired against L0 in the same fold. These descriptive averages do not turn the overlapping class training sets into independent observations.

protocol	integration	bias delta	R2	MAE	delta R2
P1_random	L0	0.000	0.859	29.908	0.000
P1_random	L1	0.000	0.829	34.137	-0.030
P1_random	L1	0.250	0.816	36.400	-0.043
P1_random	L1	0.500	0.767	42.856	-0.092
P1_random	L1	1.000	0.582	62.212	-0.277
P1_random	L3	0.000	0.864	28.915	0.005
P1_random	L3	0.250	0.859	29.628	-0.000
P1_random	L3	0.500	0.858	29.705	-0.001
P1_random	L3	1.000	0.861	29.340	0.002
P3_class_holdout	L0	0.000	0.211	50.123	0.000
P3_class_holdout	L1	0.000	0.331	45.759	0.120
P3_class_holdout	L1	0.250	0.272	48.750	0.061
P3_class_holdout	L1	0.500	0.090	55.442	-0.122
P3_class_holdout	L1	1.000	-0.372	71.729	-0.583
P3_class_holdout	L3	0.000	0.202	49.854	-0.009
P3_class_holdout	L3	0.250	0.200	50.382	-0.011
P3_class_holdout	L3	0.500	0.156	50.611	-0.056
P3_class_holdout	L3	1.000	0.115	51.142	-0.097

Table S14 Penalty sweep summaries

Source: lambda_sweep_allclasses.csv. λ=1 and bias 0/0.5 directly reuse the 132 corresponding factorial runs; R^2 and MAE match exactly. Seeds are averaged before equal-weight fold means. The apparent best weight is retrospective.

protocol	bias delta	lam	R2	MAE
P1_random	0.000	0.000	0.859	29.908
P1_random	0.000	0.100	0.864	29.272
P1_random	0.000	0.300	0.860	30.079
P1_random	0.000	1.000	0.829	34.137
P1_random	0.000	3.000	0.773	40.086
P1_random	0.000	10.000	0.720	44.697
P1_random	0.500	0.100	0.864	29.440
P1_random	0.500	0.300	0.848	32.235
P1_random	0.500	1.000	0.767	42.856
P1_random	0.500	3.000	0.618	56.892
P1_random	0.500	10.000	0.494	66.181
P3_class_holdout	0.000	0.000	0.211	50.123
P3_class_holdout	0.000	0.100	0.218	49.233
P3_class_holdout	0.000	0.300	0.329	46.303
P3_class_holdout	0.000	1.000	0.331	45.759
P3_class_holdout	0.000	3.000	0.213	49.786
P3_class_holdout	0.000	10.000	0.048	54.523
P3_class_holdout	0.500	0.100	0.246	48.822
P3_class_holdout	0.500	0.300	0.244	48.651
P3_class_holdout	0.500	1.000	0.090	55.442
P3_class_holdout	0.500	3.000	-0.248	66.893
P3_class_holdout	0.500	10.000	-0.626	76.236

Table S15 Learning curve summary

Source: learning_curves_full.csv. Repetitions are averaged within a fold before the protocol macro mean. Full-data fractions have one fit per fold; smaller fractions have three subsampling repetitions. These values concern histogram gradient boosting on stream B.

protocol	frac	R2	MAE	n train
P1_random	0.100	0.806	35.538	589.000
P1_random	0.250	0.851	30.317	1473.000
P1_random	0.500	0.877	26.586	2946.600
P1_random	0.750	0.891	24.943	4419.600
P1_random	1.000	0.898	23.950	5893.600
P2_scaffold	0.100	0.782	38.040	589.000
P2_scaffold	0.250	0.820	33.350	1473.000
P2_scaffold	0.500	0.844	30.506	2946.600
P2_scaffold	0.750	0.851	29.422	4419.600
P2_scaffold	1.000	0.857	28.528	5893.600
P3_class_holdout	0.100	0.225	49.424	694.412
P3_class_holdout	0.250	0.378	45.098	1736.588
P3_class_holdout	0.500	0.394	43.325	3473.824
P3_class_holdout	0.750	0.391	43.285	5210.765
P3_class_holdout	1.000	0.355	43.286	6948.176
P4_highTg_extrap	0.100	-4.731	88.470	588.000
P4_highTg_extrap	0.250	-4.224	83.557	1471.000
P4_highTg_extrap	0.500	-4.043	81.873	2943.000
P4_highTg_extrap	0.750	-4.001	81.183	4415.000
P4_highTg_extrap	1.000	-3.788	79.520	5887.000

Table S16 Matched few shot correction results

Source: fewshot_bias_baseline.csv. Each method uses exactly the same k calibration indices and remaining test indices within class and draw. Results are means of five draws; bias is prediction minus experiment. All archived diagnostic classes are included.

Polyphosphazenes

k	treatment	MAE	bias
5	linear	32.616	4.583
5	none	91.723	91.126
5	offset	37.607	1.176
5	retrain	64.027	61.273
10	linear	31.302	4.622
10	none	92.701	92.146
10	offset	38.297	11.502
10	retrain	52.356	47.598
25	linear	29.663	-0.212
25	none	92.060	91.594
25	offset	36.943	2.235
25	retrain	34.845	25.228
50	linear	28.659	2.659
50	none	92.369	91.698
50	offset	37.403	1.342
50	retrain	28.747	14.311

Polysiloxanes

k	treatment	MAE	bias
5	linear	38.180	-4.124
5	none	44.571	33.863
5	offset	34.460	-1.725
5	retrain	42.989	30.182
10	linear	36.874	3.297
10	none	44.283	33.925
10	offset	35.987	0.612
10	retrain	40.539	27.626
25	linear	31.959	1.066
25	none	44.536	33.922
25	offset	32.119	0.220
25	retrain	36.494	21.274
50	linear	31.098	2.262
50	none	44.430	34.306
50	offset	31.766	1.938
50	retrain	32.127	14.910
100	linear	33.102	-0.908
100	none	44.987	33.595
100	offset	33.865	-0.725
100	retrain	28.314	8.290

Polystyrenes

k	treatment	MAE	bias
5	linear	44.078	11.004
5	none	42.329	-17.018
5	offset	40.472	7.070
5	retrain	40.050	-14.839
10	linear	59.381	4.323
10	none	41.998	-16.800
10	offset	40.628	8.795
10	retrain	38.197	-12.701
25	linear	41.415	-1.830
25	none	42.888	-17.553
25	offset	39.876	-3.744
25	retrain	37.124	-13.149
50	linear	39.108	-2.318
50	none	41.814	-17.840
50	offset	38.456	-3.255
50	retrain	32.142	-11.559
100	linear	38.965	0.128
100	none	41.728	-17.232
100	offset	38.342	-0.162
100	retrain	28.730	-6.160

Polyvinyls

k	treatment	MAE	bias
5	linear	62.434	-4.051
5	none	48.414	-3.533
5	offset	51.095	-16.278
5	retrain	46.328	-5.205
10	linear	54.004	11.189
10	none	48.619	-2.915
10	offset	51.632	7.566
10	retrain	45.865	-2.351
25	linear	49.945	0.679
25	none	48.451	-3.172
25	offset	49.748	0.410
25	retrain	43.963	-2.138
50	linear	51.688	8.268
50	none	48.204	-1.572
50	offset	50.165	8.333
50	retrain	41.878	0.245
100	linear	50.466	-0.919
100	none	49.215	-3.374
100	offset	50.042	-0.410
100	retrain	39.464	-1.259

Polyacrylics

k	treatment	MAE	bias
5	linear	44.394	-1.244
5	none	34.824	-7.569
5	offset	35.881	-4.097
5	retrain	34.962	-7.803
10	linear	41.528	11.396
10	none	34.783	-7.394
10	offset	39.821	8.772
10	retrain	34.027	-3.509
25	linear	37.617	-3.601
25	none	35.106	-7.735
25	offset	36.606	-4.917
25	retrain	34.960	-7.699
50	linear	35.943	-1.574
50	none	34.774	-7.745
50	offset	35.373	-2.576
50	retrain	32.336	-7.593
100	linear	36.960	1.496
100	none	34.750	-7.340
100	offset	35.521	1.209
100	retrain	30.121	-3.258

Polyimides

k	treatment	MAE	bias
5	linear	47.624	-2.968
5	none	33.055	-5.405
5	offset	36.351	3.401
5	retrain	33.281	-5.910
10	linear	38.029	11.303
10	none	33.071	-5.363
10	offset	35.343	9.026
10	retrain	32.946	-4.688
25	linear	34.610	2.119
25	none	33.078	-5.392
25	offset	33.747	1.614
25	retrain	32.661	-4.068
50	linear	33.397	3.884
50	none	33.037	-5.299
50	offset	33.258	4.064
50	retrain	31.616	-2.246
100	linear	33.340	-4.514
100	none	33.158	-5.686
100	offset	33.222	-4.793
100	retrain	31.375	-4.697

Polyesters

k	treatment	MAE	bias
5	linear	33.342	4.258
5	none	28.582	-3.354
5	offset	32.883	10.445
5	retrain	28.583	-2.998
10	linear	30.988	-2.170
10	none	28.603	-3.477
10	offset	29.956	-2.926
10	retrain	28.312	-3.203
25	linear	29.001	-2.987
25	none	28.627	-3.562
25	offset	29.000	-3.415
25	retrain	27.674	-3.609
50	linear	29.194	-2.244
50	none	28.617	-3.653
50	offset	28.800	-2.904
50	retrain	27.172	-3.592
100	linear	28.091	-0.136
100	none	28.264	-3.440
100	offset	28.576	-0.047
100	retrain	25.370	-2.309

Table S17 Prospective abstention results

Source: selective_prediction.csv. The threshold is selected in an inner scaffold holdout and applied unchanged to the outer test. The archived per-fold records are shown; high coverage and similar full/selective error document the negative result.

protocol	fold	threshold selected inner	coverage outer	full MAE	selective MAE
P1_random	fold0	0.200	1.000	24.952	24.952
P1_random	fold1	0.200	0.999	24.020	24.005
P1_random	fold2	0.300	0.994	23.288	23.210
P1_random	fold3	0.330	0.996	23.521	23.348
P1_random	fold4	0.270	0.998	23.971	23.916
P2_scaffold	fold0	0.340	0.973	29.352	28.760
P2_scaffold	fold1	0.310	0.982	31.466	30.512
P2_scaffold	fold2	0.260	0.990	26.331	26.302
P2_scaffold	fold3	0.200	1.000	27.799	27.799
P2_scaffold	fold4	0.250	0.995	27.693	27.276
P3_class_holdout	Polyimides	0.350	0.932	33.051	31.791
P3_class_holdout	Polyoxides	0.370	0.855	33.380	31.427
P3_class_holdout	Polyesters	0.340	0.988	28.622	28.136
P3_class_holdout	Polyacrylics	0.200	1.000	34.855	34.855
P3_class_holdout	Polyamides	0.280	0.992	40.062	40.003
P3_class_holdout	Polyimines	0.200	1.000	42.726	42.726

protocol	fold	threshold selected inner	coverage outer	full MAE	selective MAE
P3_class_holdout	Polyvinyls	0.200	1.000	48.623	48.623
P3_class_holdout	Polystyrenes	0.200	1.000	42.416	42.416
P3_class_holdout	Polysiloxanes	0.200	0.987	44.402	44.669
P3_class_holdout	Polysulfides	0.200	1.000	43.434	43.434
P3_class_holdout	Polycarbonates	0.230	1.000	29.820	29.820
P3_class_holdout	Polyurethanes	0.200	1.000	35.912	35.912
P3_class_holdout	Polyphosphazenes	0.200	0.935	91.667	93.327
P3_class_holdout	Polyphenylenes	0.200	1.000	41.269	41.269
P3_class_holdout	Polyolefins	0.280	0.982	46.092	46.579
P3_class_holdout	Polydienes	0.200	1.000	51.215	51.215
P3_class_holdout	Polyhalo-olefins	0.200	1.000	48.310	48.310
P4_highTg_extrap	high	0.200	1.000	79.520	79.520

Table S18 Fingerprint sensitivity

Source: similarity_fingerprint_sensitivity.csv. These Morgan-family checks reuse the same scaffold and class-holdout predictions. Confidence summaries do not remove cross-protocol reuse of individual polymers or training-set overlap.

fingerprint	slope K per unit similarity	ci lo	ci hi	r squared
morgan_r2_2048_bit_starred	-51.042	-58.735	-43.482	0.097
morgan_r3_2048_bit_starred	-48.627	-55.573	-41.929	0.095
morgan_r2_1024_bit_starred	-51.759	-59.644	-44.055	0.097
morgan_r2_2048_bit_methylcapped	-51.712	-59.485	-44.152	0.097
morgan_r1_2048_bit_starred	-57.972	-67.556	-47.936	0.092
morgan_r2_2048_count_starred	-58.052	-67.926	-48.801	0.098

All six variants use 14,487 predictions from the five scaffold and 17 class holdouts with protocol included as a fixed effect. The reported intervals are wild-cluster summaries; representation settings and full diagnostics remain in the source CSV.

Table S19 Band gap results by model

Source: external_scores.csv. Fold means are displayed separately by model, including the mean-predictor reference. The four-model means in the manuscript exclude that reference. Band-gap MAE is in eV.

protocol	model	R2	MAE	bias	enrichment top10pct
E1_random	Dummy_mean	-0.003	1.200	-0.000	0.568
E1_random	HistGBT	0.888	0.334	0.006	7.699
E1_random	RandomForest	0.865	0.361	0.038	7.431
E1_random	Ridge	0.810	0.447	-0.004	6.636
E1_random	kNN_k5	0.602	0.582	0.346	6.116
E2_scaffold	Dummy_mean	-0.014	1.201	0.000	0.509
E2_scaffold	HistGBT	0.837	0.397	-0.008	7.315
E2_scaffold	RandomForest	0.805	0.434	0.003	6.952
E2_scaffold	Ridge	0.739	0.511	0.009	5.933
E2_scaffold	kNN_k5	0.283	0.768	0.493	4.561
E3_cluster_holdout	Dummy_mean	-0.109	1.221	0.000	0.634
E3_cluster_holdout	HistGBT	0.832	0.393	0.007	6.501
E3_cluster_holdout	RandomForest	0.790	0.450	0.035	6.693
E3_cluster_holdout	Ridge	0.734	0.515	-0.027	5.214
E3_cluster_holdout	kNN_k5	0.336	0.777	0.438	3.851
E4_high_extrap	Dummy_mean	-23.413	2.401	-2.401	2.038
E4_high_extrap	HistGBT	-3.437	0.877	-0.876	1.559
E4_high_extrap	RandomForest	-5.071	1.069	-1.069	0.000
E4_high_extrap	Ridge	-5.448	1.101	-1.099	0.480
E4_high_extrap	kNN_k5	-8.533	1.329	-1.329	0.120

Table S20 Between source variability reference

Source: reproducibility_reference.csv. Only 279 multiply reported polymers contribute to this analysis; they are not the sample size of the primary model benchmark. The statistics describe between-report variation and do not estimate an attainable model-error floor.

Statistic	N polymers	Median K	Mean K	90th percentile K
mean_loso_abs_diff	279	7.150	14.870	33.760
mean_pairwise_abs_diff	279	6.975	15.539	34.670
sd_recomputed	279	5.594	12.064	27.927
sigma_corrected	279	6.312	14.085	30.841

n sources	Number of polymers
2	132
3	91
4	42
5	9
6	3
8	1
10	1

LOSO denotes leave-one-source-out discrepancy, computed per polymer before this summary. Complete per-polymer records and class labels are in reproducibility_reference.csv in the archive. No confidence interval is inferred from these descriptive summaries.
